# Responsive Trimodal Probes for In Vivo Imaging of Liver Inflammation by Coassembly and GSH-Driven Disassembly

**DOI:** 10.34133/2020/4087069

**Published:** 2020-08-28

**Authors:** Yuxuan Hu, Yuqi Wang, Xidan Wen, Yifan Pan, Xiaoyang Cheng, Ruibing An, Guandao Gao, Hong-Yuan Chen, Deju Ye

**Affiliations:** ^1^State Key Laboratory of Analytical Chemistry for Life Science, Chemistry and Biomedicine Innovation Center (ChemBIC), School of Chemistry and Chemical Engineering, Nanjing University, Nanjing 210023, China; ^2^State Key Laboratory of Pollution Control and Resource Reuse, School of Environment, Nanjing University, Nanjing 210023, China

## Abstract

Noninvasive in vivo imaging of hepatic glutathione (GSH) levels is essential to early diagnosis and prognosis of acute hepatitis. Although GSH-responsive fluorescence imaging probes have been reported for evaluation of hepatitis conditions, the low penetration depth of light in liver tissue has impeded reliable GSH visualization in the human liver. We present a liver-targeted and GSH-responsive trimodal probe (GdNPs-Gal) for rapid evaluation of lipopolysaccharide- (LPS-) induced acute liver inflammation via noninvasive, real-time in vivo imaging of hepatic GSH depletion. GdNPs-Gal are formed by molecular coassembly of a GSH-responsive Gd(III)-based MRI probe (1-Gd) and a liver-targeted probe (1-Gal) at a mole ratio of 5/1 (1-Gd/1-Gal), which shows high *r*_1_ relaxivity with low fluorescence and fluorine magnetic resonance spectroscopic (^19^F-MRS) signals. Upon interaction with GSH, 1-Gd and 1-Gal are cleaved and GdNPs-Gal rapidly disassemble into small molecules 2-Gd, 2-Gal, and 3, producing a substantial decline in *r*_1_ relaxivity with compensatory enhancements in fluorescence and ^19^F-MRS. By combining in vivo magnetic resonance imaging (^1^H-MRI) with ex vivo fluorescence imaging and ^19^F-MRS analysis, GdNPs-Gal efficiently detect hepatic GSH using three independent modalities. We noninvasively visualized LPS-induced liver inflammation and longitudinally monitored its remediation in mice after treatment with an anti-inflammatory drug, dexamethasone (DEX). Findings highlight the potential of GdNPs-Gal for in vivo imaging of liver inflammation by integrating molecular coassembly with GSH-driven disassembly, which can be applied to other responsive molecular probes for improved in vivo imaging.

## 1. Introduction

Acute hepatitis encompasses liver diseases that cause acute inflammation or damage to hepatocytes from various etiologies (e.g., viral or bacterial infection, toxins, drugs, alcohol, and immunologic response), which can lead to severe liver dysfunction and death [1, 2]. Early diagnosis of acute hepatitis and accurate evaluation of possible etiologies are essential to patient therapy. Blood tests of liver enzymes, such as aspartate aminotransferase (AST), alanine aminotransferase (ALT), alkaline phosphatase (ALP), and *γ*-glutamyl transferase (GGT) [3, 4], are generally used to evaluate liver hepatitis. However, blood tests cannot provide morphological information to pinpoint inflammation sites in the liver; a biopsy is required to determine the hepatitis stage [5]. Abdominal ultrasound [6] or computed tomography [7], which allows for visualization of abnormal liver tissues, can facilitate diagnosis of acute hepatitis but depends on changes in organ and tissue structure. Such changes are nonspecific and lack molecular information, hindering the accuracy of early liver hepatitis diagnosis and prognosis.

Within the past two decades, biomarker-specific molecular imaging probes have emerged as indispensable tools for noninvasive early disease diagnosis [8–11], as they can offer molecular information prior to morphological change. Certain inflammation-related biomolecules (e.g., reactive oxygen species, ALP, and GGT) are significantly upregulated in inflammatory liver tissues; thus, several imaging probes have been developed to visualize liver inflammation in vivo [12–16]. Glutathione (GSH) is the most abundant biothiol that controls the redox balance in a healthy liver [17, 18], and its concentration is generally reduced in cases of liver inflammation [19, 20]. GSH is recognized as another important biomarker of liver inflammation, and many GSH-activatable fluorescent probes have been examined for the evaluation of hepatitis conditions [21–24]. For instance, Yin and colleagues developed a cyanine-based near infrared fluorescent probe for sensitive GSH detection in acetaminophen-induced inflammatory mouse livers [25]. Lee and colleagues also reported a liver cell-targeting and GSH-activatable naphthalimide-based fluorescent probe to promote the detection of GSH fluctuations in liver cells [26]. Although these probes can provide sensitive imaging signals to detect GSH in research settings, they have low tissue penetration depth and a limited signal-to-background ratio due to the inevitable absorption and scattering of light by tissues. Such limitations complicate reliable detection of GSH in the human liver given its deep tissue location.

Different from fluorescence imaging, magnetic resonance imaging (MRI) has unlimited tissue penetration depth to provide noninvasive, high-spatial-resolution images of all liver tissues in the human body [27–29]. Therefore, MRI represents a powerful diagnostic tool for liver diseases in clinics. To enable molecular MRI of GSH in vivo, several GSH-responsive Gd(III)-based MR contrast agents have been reported for contrast-enhanced imaging of the blood pool [30, 31] and tumors [32, 33]. Moreover, GSH-activatable manganese-based MRI probes have been developed to detect GSH in tumor cells [34–38]. Though these “smart” MRI probes have achieved clear responses toward GSH, none appear applicable for GSH imaging in liver tissues, presumably due to an inability to target and enter liver cells after systemic administration. We recently reported on a redox-driven disassembly strategy to develop a GSH-activatable fluorescent and MRI probe [39]; however, this probe exhibited insufficient stability and poor uptake by liver cells, hampering noninvasive real-time monitoring of hepatic GSH levels to evaluate liver inflammation.

Herein, we present a liver-targeting and GSH-responsive ^1^H-MRI/^19^F-MRS/fluorescent trimodal probe (GdNPs-Gal) via coassembly of a GSH-responsive MRI probe (1-Gd) and liver-targeting probe (1-Gal). We show that GdNPs-Gal function as a uniform and stable nanoparticle in aqueous solution, demonstrating high *r*_1_ relaxivity but quenched fluorescence and ^19^F-MRS signals. After administration into living mice, GdNPs-Gal can be efficiently delivered into the liver and taken up by liver cells through recognition between *β*-galactose (*β*-Gal) and the asialoglycoprotein receptor (ASGPR). In healthy hepatocytes, disulfide reduction and disassembly of GdNPs-Gal are initiated by abundant endogenous GSH, leading to lower ^1^H-MRI contrast but compensatory signal enhancement in fluorescence and ^19^F-MRS in the normal liver. In hepatitis cells, reduced GSH levels can slow the disulfide reduction and disassembly of GdNPs-Gal. The high *r*_1_ relaxivity and prolonged retention of GdNPs-Gal compared to its reduced small-molecule products generate higher MR contrast in inflammatory liver cells, enabling noninvasive visualization of LPS-induced liver inflammation [40, 41] via high-resolution MRI. Responsive molecular probes for noninvasive MRI of GSH levels in liver can be further applied for real-time monitoring of the anti-inflammation efficacy of dexamethasone (DEX) [42, 43] in living mice.

## 2. Results and Discussion

### 2.1. Design of GdNPs-Gal

**Figure 1:**
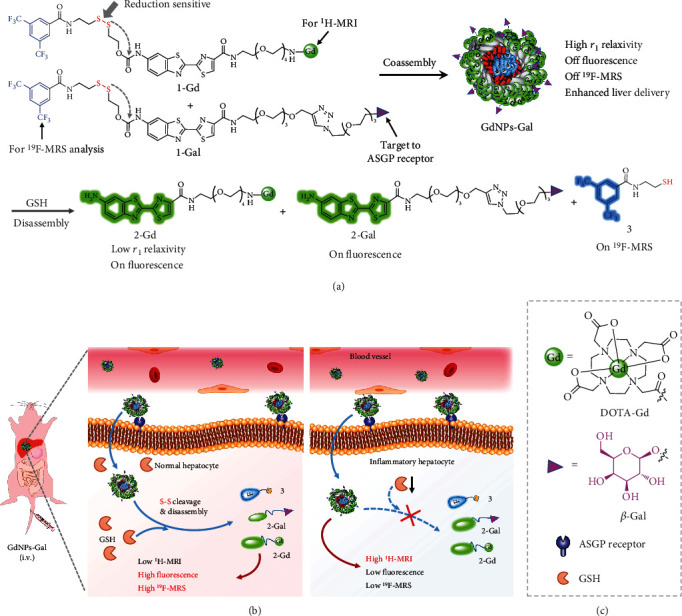
Schematic illustration shows GSH-responsive probe for in vivo imaging of liver inflammation. (a) General design of liver-targeted and GSH-responsive ^1^H-MRI/^19^F-MRS/fluorescent trimodal probe (GdNPs-Gal) via coassembly of 1-Ga and 1-Gal at a mole ratio of 5/1. (b) The proposed mechanism of GdNPs-Gal for in vivo imaging of liver inflammation. Following systemic administration into mice, GdNPs-Gal can be delivered into the liver and enter liver cells through the recognition between *β*-Gal and ASGPR. In healthy hepatocytes, disulfide reduction and disassembly of GdNPs-Gal are initiated by the abundant endogenous GSH, leading to reduction in ^1^H-MRI contrast but enhanced fluorescence and ^19^F-MRS signals in the normal liver; in hepatitis cells, the reduced GSH levels can slow down the disulfide reduction and disassembly of GdNPs-Gal, thereby producing a strong MR contrast but low fluorescence and ^19^F-MRS signals in the inflammatory liver. (c) Chemical structure of DOTA-Gd and *β*-Gal.

Figure 1(a) shows the general design of GdNPs-Gal, formed through coassembly of a GSH-sensitive MRI probe 1-Gd and a liver-targeting probe 1-Gal. These probes share a molecular template, consisting of a hydrophobic 3,5-bis(trifluoromethyl)benzene, a quenched amino oxyluciferin fluorophore, and a GSH-cleavable disulfide linker. Based on this template, 1-Gd is designed by covalently linking a paramagnetic Gd(III)-chelate for MRI; 1-Gal is designed by conjugating a PEGylated *β*-Gal for liver targeting. The molecular length of 1-Gal is longer than that of 1-Gd, enabling extension of *β*-Gal ligands to the outer layer of GdNPs-Gal for liver targeting. The 3,5-bis(trifluoromethyl)benzene and amino oxyluciferin are used because their hydrophobicity and rigidity can produce strong intermolecular interactions (e.g., *π*-*π* stacking and hydrophobic interactions) to promote efficient molecular self-assembly [44, 45]. Moreover, the six magnetically equivalent ^19^F nuclei can offer strong fluorine magnetic spectroscopic (^19^F-MRS) signals in solution, and the amino oxyluciferin can provide efficient fluorescence for cell imaging. We speculated that 1-Gd and 1-Gal might coassemble in aqueous solution to form liver-targeted and GSH-responsive fluorogenic and magnetic nanoparticles (GdNPs-Gal), which demonstrated great longitudinal *r*_1_ relaxivity but quenched fluorescence and ^19^F-MRS signals due to a paramagnetic relaxation effect (PRE) [46] along with a spin-spin relaxation quenching effect [42]. After systemic administration, we noted that GdNPs-Gal could specifically target and enter liver cells through efficient recognition between *β*-Gal ligands on the surface of GdNPs-Gal and ASGPR on cell membranes (Figure 1(b)). In healthy hepatocytes, abundant intracellular GSH could cleave disulfides within GdNPs-Gal, triggering their rapid disassembly into small molecules 2-Gd, 2-Gal, and 3. As the molecular size of 2-Gd is much smaller than that of GdNPs-Gal, *r*_1_ relaxivity declined; meanwhile, fluorescence and ^19^F-MRS signals could be switched on as the amino oxyluciferin fluorophore was uncaged in 2-Gd and 2-Gal and the PRE plus spin-spin relaxation quenching effects were abolished in 3, respectively. Therefore, reduced ^1^H-MRI contrast but compensatory signal enhancement in fluorescence and ^19^F-MRS was observed in the normal liver. GSH levels were downregulated in hepatitis cells due to inflammation-related oxidative stress, which could decelerate disulfide cleavage and delay GdNPs-Gal disassembly. The remaining GdNPs-Gal exhibited higher *r*_1_ relaxivity and prolonged retention relative to 2-Gd, producing higher MR contrast in the inflammatory liver than in the normal liver. Thus, GdNPs-Gal may be feasible for noninvasive detection of liver inflammation through high-resolution MR imaging of reduced hepatic GSH levels in vivo. The remarkably amplified fluorescence and ^19^F-MRS signals arising from GHS-driven disulfide cleavage and disassembly in healthy liver cells, but not in hepatitis cells, could also provide complementary information to report and verify hepatic GSH levels in vivo and ex vivo.

### 2.2. Preparation and Characterization of GdNPs-Gal In Vitro

We first optimized the chemical structure of the molecular template for 1-Gd and 1-Gal to propel molecular self-assembly and improve the stability of resulting nanoparticles (NPs) in aqueous solution. Three compounds, 1-a, 1-b, and 1-c, with linkers of varying lengths at the carboxylic end of amino oxyluciferin, were synthesized (Figure S1). Their self-assembly property in aqueous solution was then examined. Dynamic light scattering (DLS) and transmission electron tomography (TEM) analyses revealed that all three compounds could undergo molecular self-assembly to form nanostructures upon dispersion in aqueous solution (Figures S2a and S2b). Among them, only compound 1-c with a hydrophilic polyethylene glycol linker (PEG4) could form monodispersed and stable NPs, with an average diameter of ~50 nm. ^1^H NMR analysis of 1-c in DMSO-*d*_6_ containing different D_2_O content demonstrated that self-assembly was likely driven by *π*-*π* stacking interactions that occurred in both the 3,5-bis(trifluoromethyl)benzene ring and the amino oxyluciferin scaffold (Figure S3), which could produce a strong spin-spin relaxation effect to completely turn off the ^19^F-MRS signal (Figure S2c). We further demonstrated that 1-c within assembled NPs could be reduced by GSH, subsequently triggering NP disassembly to turn on fluorescence and ^19^F-MRS signals (Figure S4). These results suggest that 1-c is an effective molecular template for self-assembly and GSH-driven disassembly, which we then employed to synthesize GSH-responsive probes 1-Gd and 1-Gal according to the approaches outlined in Schemes S1 and S2. In addition, GSH-inert control probes 1-Gd-Ctrl and 1-Gal-Ctrl were synthesized by replacing the disulfide bond with a C-C bond (Schemes S3 and S4).

**Figure 2:**
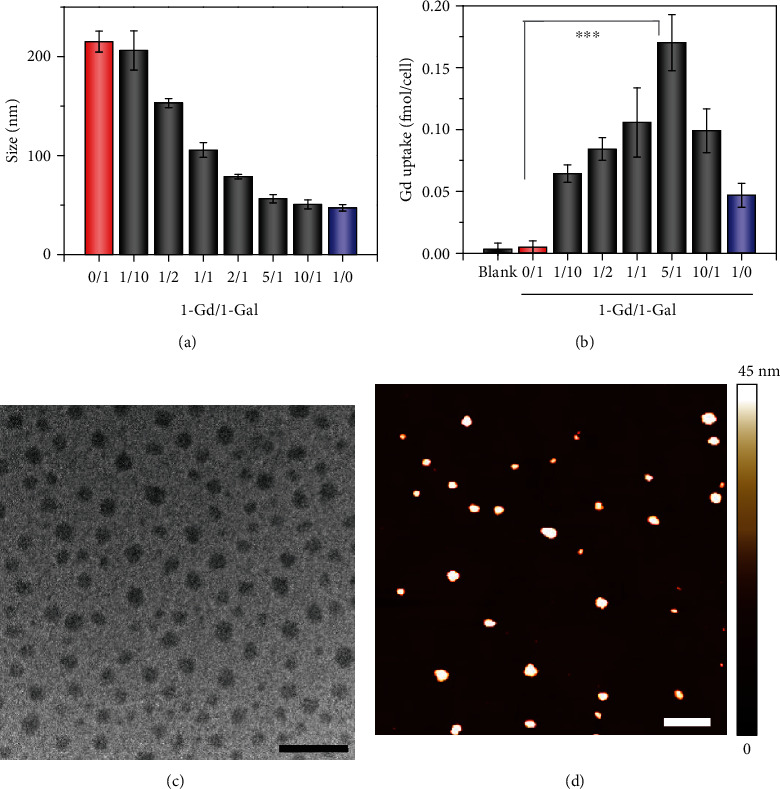
Characterization of GdNPs-Gal in vitro. (a) DLS analysis shows the mean size of nanoparticles coassembling from 1-Gd and 1-Gal at varying mole ratios. (b) ICP-MS analysis shows the uptake of Gd in HepG2 cells after incubation with different coassembled nanoparticles. Data denote mean ± standard deviation (SD, *n* = 3, ^∗∗∗^*P* < 0.001). (c) TEM and (d) AFM analysis of GdNPs-Gal coassembling from 1-Gd and 1-Gal at a mole ratio of 5. Scale bars: 200 nm in TEM and 500 nm in AFM.

As with 1-c, 1-Gd and 1-Gal could each self-assemble into monodispersed NPs, with a mean hydrodynamic size of ~50 nm and ~220 nm, respectively (Figure S5). The critical micellar concentration (CMC) of probe 1-Gd was ~12.0 *μ*M, larger than that of 1-Gal (CMC ≈ 5.5 *μ*M) (Figure S6). The smaller size but larger CMC observed in NPs of 1-Gd (1-GdNPs) relative to 1-Gal-based NPs (1-GalNPs) was presumably due to the greater hydrophilicity of DOTA-Gd-chelate in 1-Gd compared to the *β*-Gal ligand in 1-Gal. To prepare GdNPs-Gal with a small size and sufficient *β*-Gal ligands to maximize cellular uptake of Gd(III) for MRI, coassembly of 1-Gd and 1-Gal at varying molecular ratios was optimized. DLS analysis showed that 1-Gd and 1-Gal could coassemble well into monodispersed NPs, and the hydrodynamic size of NPs decreased as the ratio of 1-Gd and 1-Gal (1-Gd/1-Gal) increased (Figures 2(a) and S7). When the 1-Gd/1-Gal ratio increased to 5 or higher, the mean hydrodynamic size of NPs declined to be as small as that of 1-GdNPs. Subsequent ICP-MS analysis indicated that Gd(III) uptake in ASGPR-positive HepG2 cells increased as the 1-Gd/1-Gal ratio increased and peaked at ~0.18 fmol/cell when the ratio was roughly 5 (Figure 2(b)), substantially higher than that of 1-GdNPs (~0.05 fmol/cell). These findings imply that coassembly of 1-Gd and 1-Gal at a ratio of 5 was optimal for building GdNPs-Gal, which ensured a small hydrodynamic size and large uptake of Gd(III). DLS analysis revealed a fast coassembly process to form GdNPs-Gal in a PBS buffer (Figure S8). TEM and atomic-force microscopy analyses confirmed the formation of uniform and spherical NPs (Figures 2(c) and 2(d)). When preformed 1-GdNPs and 1-GalNPs at a ratio of 5 were mixed in aqueous solution, DLS analysis revealed distinct peaks at ~50 nm and ~220 nm (Figure S9), countering that of GdNPs-Gal and verifying that GdNPs-Gal formed via efficient coassembly of 1-Gd and 1-Gal probes (5/1). Given the coexistence of DOTA-Gd chelates and *β*-Gal ligands in GdNPs-Gal, the CMC (~7.5 *μ*M) and zeta potential (−40.0 ± 2.7 mV) were each smaller than those of 1-Gd but larger than those of 1-Gal (Figure S10). The small CMC and highly negative zeta potential helped GdNPs-Gal maintain high stability under physiological conditions. After incubation in a PBS buffer or cell culture medium (DMEM) containing 10% serum for 1 week, GdNPs-Gal maintained their monodispersed size, and their fluorescence and *T*_1_ relaxation time exhibited negligible changes (Figure S11).

### 2.3. GSH-Mediated Disulfide Reduction and Disassembly In Vitro

**Figure 3:**
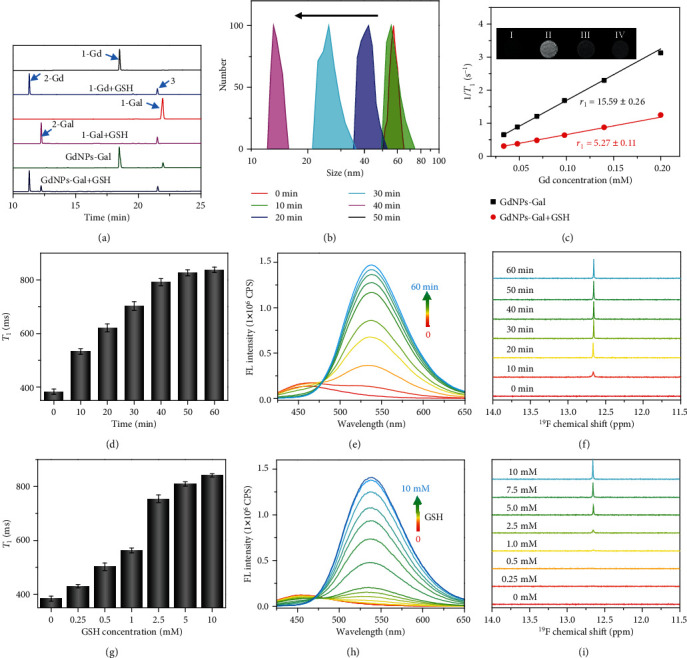
Response of GdNPs-Gal toward GSH. (a) HPLC analysis of 1-Ga, 1-Gal, and GdNPs-Gal before and after incubation with GSH. (b) DLS analysis of GdNPs-Gal (200 *μ*M) following incubation with GSH (10 mM) for 0–50 min. (c) Plots of 1/*T*_1_ versus Gd concentration determine the *r*_1_ relaxivities of GdNPs-Gal before (black) and after (red) incubation with GSH (10 mM, 60 min). Inset: *T*_1_-weighted MR images of the following: I: PBS; II: GdNPs-Gal (200 *μ*M); III: GdNPs − Gal (200 *μ*M) + GSH (10 mM, 60 min); IV: Dotarem (200 *μ*M). (d) *T*_1_ values, (e) fluorescence spectra, and (f) ^19^F NMR spectra of GdNPs-Gal (200 *μ*M) upon incubation with GSH (10 mM) for 0–60 min. (g) *T*_1_ values, (h) fluorescence spectra, and (i) ^19^F NMR spectra of GdNPs-Gal (200 *μ*M) upon incubation with varying concentrations of GSH for 60 min. The fluorescence spectra of GdNPs-Gal were measured after 1 to 10 dilutions with PBS buffer. Data denote mean ± SD (*n* = 3).

GSH-mediated disulfide reduction and disassembly of GdNPs-Gal were investigated upon incubation with GSH (10 mM, PBS, pH 7.4). High-performance liquid chromatography analysis showed that 1-Gd (*t*_R_ = 18.48 min) and 1-Gal (*t*_R_ = 21.98 min) in GdNPs-Gal could be gradually reduced and ultimately converted into cleaved products 2-Gd (*t*_R_ = 11.28 min), 2-Gal (*t*_R_ = 12.22 min), and 3 (*t*_R_ = 21.51 min) after 1 h (Figures 3(a) and S12). Along with disulfide reduction, a continuous decline in hydrodynamic size was observed: GdNPs-Gal decreased from ~58 nm initially to ~12 nm after 40 min, and negligible NPs were detected by DLS after 50 min (Figures 3(b) and S13). Conversely, the incubation of GSH-inert GdNPs-Ctrl formed by coassembly of 1-Gd-Ctrl and 1-Gal-Ctrl (mole ratio = 5/1) with GSH did not elicit marked changes in retention times or average hydrodynamic size (Figures S14 and S15); hence, GSH-mediated disulfide cleavage appeared pivotal in triggering disassembly of GdNPs-Gal into small molecules 2-Gd, 2-Gal, and 3.

We next monitored changes in the *r*_1_ relaxivity of GdNPs-Gal after incubation with GSH. As shown in Figure 3(c), the initial *r*_1_ relaxivity of GdNPs-Gal was 15.6 ± 0.3 mM^−1^ s^−1^ (per Gd unit) at 0.5 T, ~2.9-fold higher than that of Dotarem (5.4 ± 0.3 mM^−1^ s^−1^) and presumably attributable to the prolonged molecule tumbling time (*τ*_R_) caused by the increased molecular size of assembled NPs. After reacting with GSH, GdNPs-Gal disassembled completely and the *r*_1_ relaxivity declined to 5.3 ± 0.1 mM^−1^ s^−1^, near that of Dotarem (Figure 3(c)). Given lower *r*_1_ relaxivity, the *T*_1_ relaxation time of GdNPs-Gal (200 *μ*M) increased progressively from 382 ms to 838 ms upon incubation with GSH (10 mM, pH 7.4) for 1 h (Figures 3(d) and S16a), which significantly reduced ^1^H-MRI contrast as revealed by *T*_1_-weighted MR imaging of the incubation solutions (Figure 3(c), inset). Subsequent measurement of fluorescence spectra indicated that GdNPs-Gal initially displayed a weak fluorescence emission at 450 nm. Upon incubation with GSH, the fluorescence could be gradually switched on and shifted to 535 nm as the amino oxyluciferin fluorophore was uncaged (Figures 3(e) and S16b). After 1 h, a maximum ~42-fold turn-on ratio in fluorescence intensity was achieved. We also acquired ^19^F-NMR spectra of GdNPs-Gal (400 MHz). As shown in Figure 3(f), the ^19^F-NMR peak of GdNPs-Gal at *δ*_F_ = 12.56 ppm (respective to sodium trifluoroacetate, at *δ*_F_ = 0 ppm) was hardly observed initially, indicating that the ^19^F-MRS signal of 3,5-bis(trifluoromethyl)benzene was completely quenched through PRE and self-assembly-induced spin-spin relaxations. Upon incubation with GSH, a sharp ^19^F-NMR peak at *δ*_F_ = 12.56 ppm appeared and the intensity increased over time. The ^19^F-MRS signal peaked after 1 h, with a signal enhancement factor of ~30 toward GSH (Figure S16c). By contrast, the ^1^H-MRI, fluorescence, and ^19^F-MRS signals of GdNPs-Ctrl changed little with exposure to GSH (Figure S17), aligning with the maintained compounds and NPs analyzed by HPLC and DLS (Figures S14 and S15). These data confirm that GSH could efficiently activate GdNPs-Gal via rapid disulfide reduction and disassembly, leading to greatly reduced ^1^H-MRI contrast and compensatory enhancement in fluorescence and ^19^F-MRS.

After confirming the fast response of GdNPs-Gal toward GSH, the sensitivity of GdNPs-Gal in detecting GSH was examined. GdNPs-Gal (200 *μ*M) was incubated with varying concentrations of GSH in the PBS buffer for 1 h, and the *T*_1_ relaxation time in each solution was measured.  Figure 3(g) illustrates that the *T*_1_ relaxation time of GdNPs-Gal (200 *μ*M, PBS buffer) extended as the GSH concentration increased in the solutions. The plot of the *T*_1_ relaxation time versus GSH concentration depicts a linear correlation within 0.25–2.5 mM, and the limit of detection (LOD) was ~0.18 mM (Figure S18). As with *T*_1_ values, a concentration-dependent increment in the fluorescence intensity and ^19^F-MRS signal was observed in the solutions (Figures 3(h) and 3(i)). The LOD for GSH was ~0.7 *μ*M using fluorescence and ~0.25 mM using ^19^F-MRS, respectively (Figure S18). The much lower LOD for fluorescence was attributed to the higher sensitivity of fluorescence relative to that of ^1^H-MRI and ^19^F-MRS. Moreover, the selectivity of GdNPs-Gal toward GSH over other reductive substances and biologically relevant metal ions was investigated. As with GSH, GdNPs-Gal showed a response to cysteine and homocysteine, two other important endogenous free biothiols that contribute to the intracellular redox environment (Figure S19). No apparent changes in *T*_1_ relaxation time and fluorescence intensity were observed upon exposure to other nonthiol-reducing agents (e.g., VC and NADPH), metal ions (e.g., Mg^2+^, Zn^2+^, and Cu^2+^) or oxidized GSH. Therefore, GdNPs-Gal demonstrated good specificity to biothiols compared to other endogenous agents.

### 2.4. Imaging of GSH in Cells

To further substantiate the capacity of GdNPs-Gal to detect endogenous GSH levels in cells, the cytotoxicity against HepG2 cells was first evaluated using a standard 3-(4,5-dimethylthiazol-2-yl)-2,5-diphenyltetrazolium bromide assay. Results showed that GdNPs-Gal had little effect on cell viability, suggesting good biocompatibility for cell studies (Figure S20). We then applied GdNPs-Gal (200 *μ*M) to detect endogenous GSH in lysed HepG2 cells by acquiring *T*_1_ relaxation time, fluorescence, and ^19^F NMR spectra. As indicated in Figure S21, HepG2 cell lysates after being incubated with GdNPs-Gal displayed a long *T*_1_ relaxation time, strong fluorescence at 535 nm, and a distinct ^19^F NMR signal at *δ*_F_ = 12.56 ppm in contrast to that of GdNPs-Gal-Ctrl. These results suggest that GdNPs-Gal could be activated by endogenous GSH in cell lysates.

**Figure 4:**
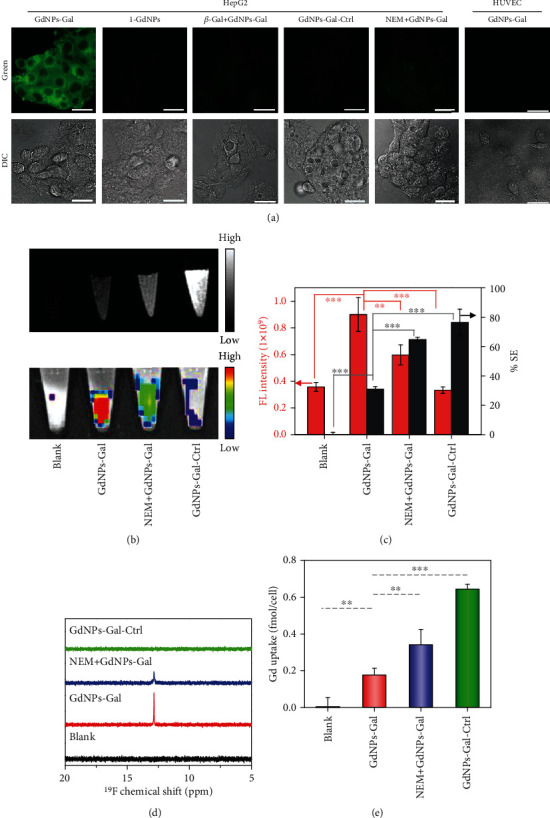
Imaging of GSH in cells. (a) Fluorescence imaging of HepG2 or HUVEC cells following incubation with 200 *μ*M GdNPs-Gal, 1-GdNPs, GdNPs-Gal-Ctrl, GdNPs-Gal plus *β*-Gal (20 mM), or GdNPs-Gal plus NEM (100 *μ*M, 30 min) for 4 h. Scale bars: 20 *μ*M. (b) *T*_1_-weighted MR (up) and fluorescence (down) images of blank HepG2 cell pellets or HepG2 cell pellets after incubation with 200 *μ*M GdNPs-Gal, 1-GdNPs, GdNPs-Gal-Ctrl, or GdNPs-Gal plus NEM (100 *μ*M, 30 min) for 4 h. (c) Quantification of the average fluorescence intensity (red) and percentage signal enhancement (% SE, black) of the HepG2 cell pellets in (b). (d) ^19^F NMR spectra of HepG2 cell lysates from cell pellets in (b). (e) Quantification of the uptake of Gd of the indicated cell pellets in (b). Data denote mean ± SD (*n* = 3). ^∗∗^*P* < 0.01, ^∗∗∗^*P* < 0.001.

Next, GdNPs-Gal were used to detect GSH levels in living HepG2 cells. Incubation conditions were optimized by flow cytometry and fluorescence imaging assays (Figures S22 and S23). Intracellular fluorescence became brighter as the incubation time prolonged or the concentration of GdNPs-Gal increased. When HepG2 cells were incubated with GdNPs-Gal (200 *μ*M) for 4 h, strong green fluorescence was observed inside cells (Figure 4(a)). Colocalization studies revealed that activated green fluorescence was mainly distributed in lysosomes at the first 0.5 h, and after 4 h, some fluorescence could escape from lysosomes and then diffuse into the cytosol (Figure S24). By contrast, negligible fluorescence was found in HepG2 cells incubated with *β*-Gal free 1-GdNPs, GdNPs-Gal plus free *β*-Gal, or ASGPR-deficient HUVEC cells incubated with GdNPs-Gal, conveying an important role of *β*-Gal in enhancing cellular uptake of GdNPs-Gal (Figure 3(a)). When *N*-ethylmaleimide (NEM) was added into the culture medium to scavenge endogenous GSH, the strong green fluorescence was greatly suppressed. In addition, HepG2 cells incubated with GdNPs-Gal-Ctrl showed significantly weaker intracellular fluorescence. These results demonstrate that GdNPs-Gal could efficiently enter HepG2 cells via ASGPR-mediated uptake, followed by activation by intracellular GSH, resulting in strong green fluorescence.

Encouraged by our fluorescence imaging results, we investigated the detection of GSH levels in HepG2 cell pellets via complementary ^1^H-MRI, fluorescence imaging, and ^19^F-MRS multiplex analysis. As displayed in Figures 4(b)–4(d), HepG2 cells incubated with GdNPs-Gal exhibited brighter *T*_1_-weighted MRI contrast, stronger fluorescence intensity, and a higher ^19^F-NMR peak relative to blank cells due to intracellular uptake and activation of GdNPs-Gal by GSH. When HepG2 cells were pretreated with NEM to downregulate the intracellular GSH concentration or incubated with GSH-inert GdNPs-Gal-Ctrl, the MRI contrast in pellets was further enhanced, whereas the fluorescence and ^19^F-MRS signals were parallelly reduced. These results were consistent with those of fluorescence live cell imaging, implying that intracellular GSH is important for controlling ^1^H-MRI, fluorescence, and ^19^F-MRS signals of GdNPs-Gal in living cells. Subsequent ICP-MS analysis revealed that intracellular Gd(III) uptake in HepG2 cells incubated with GdNPs-Gal was ~0.18 fmol/cell, which increased to ~0.38 fmol/cell in NEM-pretreated HepG2 cells and ∼0.64 fmol/cell in HepG2 cells incubated with GdNPs-Gal-Ctrl (Figure 4(e)). These differences in Gd(III) uptake matched well with the MRI contrast observed in cell pellets, which were probably correlated to the varying degrees of intracellular NP disassembly caused by GSH. In healthy HepG2 cells, the hydrophilic small-molecule 2-Gd resulting from efficient GSH-driven disassembly of GdNPs-Gal might be easily expelled from cells, while the remaining GdNPs-Gal and GdNPs-Gal-Ctrl could be trapped inside cells. In NEM-pretreated HepG2 cells, the reduced intracellular GSH concentration could slow the disassembly and prolong retention of GdNPs-Gal in cells, leading to enhanced Gd(III) uptake. Such an increased intracellular Gd(III) concentration plus a higher *r*_1_ relaxivity of GdNPs-Gal compared to 2-Gd could produce significantly higher MRI contrast in GSH-deficient liver cells. GSH-activated fluorescence and ^19^F-MRS could also provide additional sensitive and specific signals to differentiate GSH-rich and GSH-deficient cells. Therefore, GdNPs-Gal appear capable of reporting on endogenous GSH levels using multiplex signals offered by GdNPs-Gal.

### 2.5. Imaging of Hepatic GSH in LPS-Induced Inflammatory Mice

**Figure 5:**
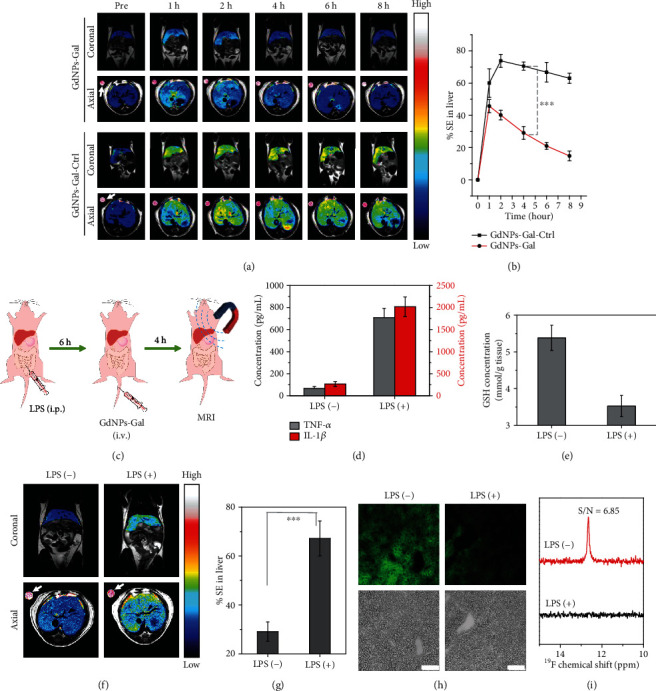
Detection of GSH in living mice with liver inflammation using GdNPs-Gal. (a) Longitudinal, coronal, and axial *T*_1_-weighted MR images of livers in healthy mice receiving i.v. injection of 0.1 mmol kg^−1^ of GdNPs-Gal (up) or GdNPs-Gal-Ctrl (down). Images were acquired before (pre), 1, 2, 4, 6, and 8 h after injection. The MR contrast in livers is shown in pseudo rainbow color for comparison; white arrows in axial images indicate the Dotarem (1 mM) solution as the internal standard. (b) Average % SE in livers of healthy mice after i.v. injection of GdNPs-Gal or GdNPs-Gal-Ctrl. (c) Schematic illustration of MRI of hepatic GSH in LPS-induced inflammatory mice. (d) Measurement of TNF-*α* (grey) and IL-1*β* (red) in mouse serums before and 6 h after i.p. injection of LPS (20 mg kg^−1^). (e) Quantification of hepatic GSH level before and 6 h after i.p. injection of LPS. (f) Coronal and axial *T*_1_-weighted MR images and (g) averaged % SE of livers in healthy mice (LPS (-)) or inflammatory mice (LPS (+)) at 4 h post i.v. injection of GdNPs-Gal (0.1 mmol kg^−1^). The MR contrast in livers is shown in pseudo rainbow color for comparison; white arrows in axial images indicate the Dotarem (1 mM) solution as the internal standard. (h) Fluorescence images of liver tissue slices and (i) ^19^F NMR spectra of liver tissue homogenates dissected from healthy and inflammatory mice at 4 h post i.v. injection of GdNPs-Gal (0.1 mmol kg^−1^). Scale bars: 100 *μ*M. Data denote mean ± SD (*n* = 3, ^∗∗∗^*P* < 0.001).

The ability of GdNPs-Gal to enter liver tissue and noninvasively detect liver GSH in living mice was investigated next. We first examined the blood half-life (*t*_1/2_) of GdNPs-Gal in mice, which was ~1.4 h (Figure S25). *T*_1_-weighted MR images were then acquired prior to (pre), 1, 2, 4, 6, and 8 h following intravenous (i.v.) injection of GdNPs-Gal or GdNPs-Gal-Ctrl (0.1 mmol kg^−1^) into heathy mice. As illustrated in Figure 5(a), bright *T*_1_-weighted MR contrast in the liver was observed at 1 h postinjection of GdNPs-Gal, indicating that GdNPs-Gal could be delivered into the liver. The enhanced MRI contrast rapidly declined thereafter, presumably due to the reduced *r*_1_ relaxivity and fast washout of cleaved products resulting from GSH-driven disassembly. Conversely, the *T*_1_-weighted MR contrast in the liver of mice receiving an i.v. injection of GdNPs-Gal-Ctrl increased continuously within the first 2 h and maintained a high contrast for more than 8 h. The signal enhancement (% SE) in livers treated with GdNPs-Gal-Ctrl was ~70% at 4 h, ~2.4-folds higher than that treated with GdNPs-Gal (~29%) (Figure 5(b)). These results suggest that GdNPs-Gal can enter the liver and facilitate rapid disassembly triggered by abundant hepatic GSH in healthy mice, leading to a lower MRI contrast relative to that of GSH-inert GdNPs-Gal-Ctrl.

We next examined the biodistribution of GdNPs-Gal and GdNPs-Gal-Ctrl in healthy mice (Figure S26). ICP-MS analysis showed that GdNPs-Gal-Ctrl was mainly distributed in the liver and spleen. The ID % g^−1^ was found to be ~37.8% (liver) and ~23.4% (spleen), respectively, significantly higher than that in GdNPs-Gal-treated mice (~16.3% in the liver and ~8.4% in the spleen). In contrast, the ID % g^−1^ of Gd(III) in the kidneys of GdNPs-Gal-Ctrl-treated mice was only ~1.6%, significantly lower than that in GdNPs-Gal-treated mice (~7.9%). These results corroborated the significant difference in MR contrast between GdNPs-Gal- and GdNPs-Gal-Ctrl-treated mice (Figure 5(a)), supporting that the GSH-driven disassembly of GdNPs-Gal could enhance renal clearance and facilitate fast washout of GdNPs-Gal from the normal liver.

GdNPs-Gal was then applied to detect hepatic GSH in living mice with liver inflammation established by intraperitoneal (i.p.) injection of lipopolysaccharides (LPS, 20 mg kg^−1^) (Figure 5(c)). Blood tests and hematoxylin and eosin (H&E) staining (Figures 5(d) and S27) showed that proinflammatory cytokines such as IL-1*β* and TNF-*α* were significantly upregulated in the blood, and a clear inflammatory lesion was observed in liver tissues, confirming an inflammatory response. We subsequently measured the GSH concentration in excised liver tissues.  Figure 5(e) reveals only ~3.5 mmol g^−1^ GSH in the LPS-treated liver, ~35% lower than that in the normal liver (~5.4 mmol g^−1^). The ability of GdNPs-Gal to visualize liver inflammation in living mice was then investigated through MR imaging of liver GSH levels. Mice were untreated or treated with LPS for 6 h, followed by i.v. injection of GdNPs-Gal (0.1 mmol kg^−1^). *T*_1_-weight MR images showed that the contrast in inflammatory livers rapidly increased and peaked at 2 h postinjection, different from those in untreated mice (peaked at ~1 h) (Figure S28). The maximum MRI signal in inflammatory livers was higher and declined more slowly than that in normal livers. At 4 h, the % SE in inflammatory livers reached ~67%, ~2.2-folds higher than that in normal livers (~29%) (Figures 5(f) and 5(g)). These findings demonstrate that GdNPs-Gal could produce a higher MRI contrast in the liver of inflammatory mice compared to in healthy mice due to a reduced hepatic GSH level that caused less disassembly of GdNPs-Gal. Subsequent fluorescence imaging of liver tissue slices and ^19^F-MRS analysis of liver tissue homogenates showed that fluorescence and ^19^F-MRS signals were much weaker in LPS-treated mice relative to nontreated healthy mice (Figures 5(h) and 5(i)), substantiating greatly suppressed GSH-triggered disulfide reduction and disassembly of GdNPs-Gal in inflammatory livers. Furthermore, coronal *T*_1_-weighted MR images showed that the bladder was much darker while the gallbladder was brighter in LPS-treated mice than in healthy mice at 4 h postinjection of GdNPs-Gal. These images imply that, in healthy mice, GdNPs-Gal were excreted via the renal system due to efficient disassembly caused by a high level of hepatic GSH. In inflammatory mice, GdNPs-Gal were mainly excreted via the hepatobiliary systems as the lower level of hepatic GSH was insufficient to trigger GdNPs-Gal disassembly (Figure S29). The subsequent ICP-MS analysis of Gd(III) in the urine and feces confirmed the efficient renal clearance of GdNPs-Gal after i.v. injection into healthy mice (Figure S30), supporting that GSH-driven disassembly of GdNPs-Gal in the liver of healthy mice could promote in vivo clearance via the renal system.

### 2.6. Monitoring GSH Recovery in Inflammatory Mice Receiving DEX Therapy

**Figure 6:**
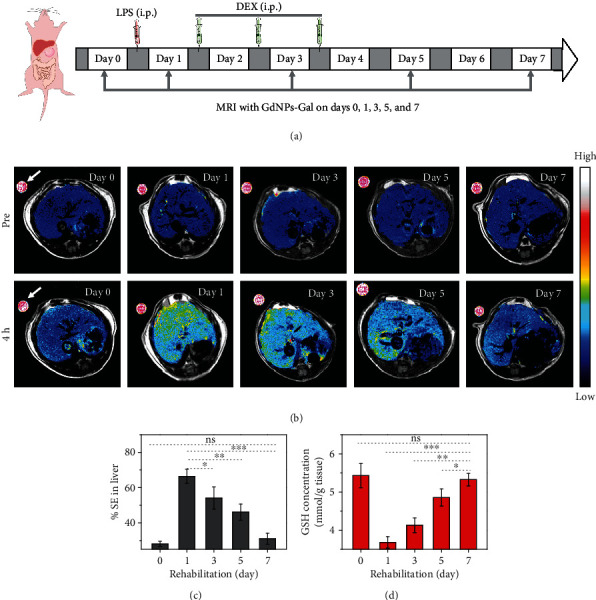
Monitoring of hepatic GSH recovery in inflammatory mice receiving DEX therapy. (a) Schematic illustration of GdNPs-Gal-assisted MRI of hepatic GSH levels in inflammatory mice receiving DEX therapy. (b) Axial *T*_1_-weighted MR images and (c) average % SE of inflammatory livers in LPS-treated mice on days 0, 1, 3, 5, and 7. The MR images were acquired before (pre) and at 4 h post i.v. injection of GdNPs-Gal (0.1 mmol kg^−1^). LPS (20 mg kg^−1^) was i.p. injected into mice on day 0, followed by i.p. injection of DEX (4 mg kg^−1^) to treat inflammation on the 1st, 2nd, and 3rd days. The MR contrast in livers is shown in pseudo rainbow color for comparison; white arrows in the images indicate the Dotarem (1 mM) solution as the internal standard. (d) Quantification of hepatic GSH concentration before and on days 1, 3, 5, and 7 after LPS treatment, followed by DEX therapy. Data denote mean ± SD (*n* = 3, ∗*P* < 0.05, ^∗∗^*P* < 0.01, ^∗∗∗^*P* < 0.001).

Given the high feasibility to detect reduced hepatic GSH levels in inflammatory mice, GdNPs-Gal were applied to noninvasively monitor GSH recovery in LPS-treated mice that received anti-inflammatory DEX therapy [47]. Mice were i.p injected with LPS to induce liver inflammation, followed by i.p. injection of DEX on the 1^st^, 2^nd^, and 3^rd^ days to alleviate inflammation. GdNPs-Gal were i.v. injected into mice prior to (day 0) and on the 1^st^, 3^rd^, 5^th^, and 7^th^ days after LPS treatment, and *T*_1_-weighted MR images were acquired at 4 h (Figure 6(a)). As shown in Figure 6(b), on the 1^st^ day after LPS treatment, MR images in livers of inflammatory mice were much brighter than those of mice prior to LPS treatment. The enhanced MRI contrast gradually decreased when inflammatory mice were treated with DEX. The % SE in the livers of mice was ~66% on day 1 after LPS treatment and declined gradually to ~30% on the 7^th^ day, similar to mice prior to LPS treatment (~28%, Figure 6(c)). The lower MRI contrast in inflammatory mice could be attributable to the recovering level of hepatic GSH after DEX therapy, which was further confirmed by quantifying the GSH concentration in excised liver tissues (Figure 6(d)). Therefore, these findings indicate that GdNPs-Gal are appropriate for detecting hepatic GSH fluctuation in vivo and could act as an efficient MRI contrast agent for noninvasive imaging of liver inflammation and monitoring of anti-inflammatory efficiency.

## 3. Conclusion

In summary, we developed a liver-targeted and GSH-responsive ^1^H-MRI/^19^F-MRS/fluorescence trimodal probe, GdNPs-Gal, through coassembly of a GSH-cleavable Gd(III)-based MRI probe (1-Gd) and a *β*-Gal-containing liver-targeted probe (1-Gal) and demonstrated its capacity for noninvasive imaging of liver inflammation in vivo. GdNPs-Gal had high *r*_1_ relaxivity (~15.6 ± 0.3 mM^−1^ s^−1^, 0.5 T) but low fluorescence and ^19^F-MRS signals; efficient disassembly of GdNPs-Gal into small molecules 2-Gd, 2-Gal, and 3 was achieved upon reduction by GSH, eliciting a clear decrease in *r*_1_ relaxivity (5.3 ± 0.1 mM^−1^ s^−1^) but parallel large enhancements in fluorescence (~42-folds) and ^19^F-MRS (~30-folds). Our in vivo studies showed that GdNPs-Gal could preferentially accumulate in the liver following i.v. injection into living mice, exhibiting significantly higher ^1^H-MRI contrast but lower fluorescence and ^19^F-MRS signals in the LPS-induced inflammatory liver relative to the nontreated normal liver. Such distinctions in ^1^H-MRI/^19^F-MRS/fluorescence could allow GdNPs-Gal to differentiate GSH-rich normal liver cells from GSH-deficient inflammatory liver cells in vivo and ex vivo. Using GdNPs-Gal, we further monitored the therapeutic efficiency of DEX against LPS-treated inflammatory mice via noninvasive MR imaging of hepatic GSH levels, revealing efficient recovery of hepatic GSH concentration after anti-inflammation therapy with DEX. In light of molecular coassembly with precisely controlled composition, preferential liver accumulation, high biocompatibility, and GSH-driven disassembly with complementary changes in MRI contrast, fluorescence emission, and ^19^F-MRS intensity, GdNPs-Gal hold great promise for liver inflammation imaging. Our reported co-self-assembly and disassembly approach to designing a liver-targeted and GSH-responsive trimodal probe could inform the design of other biomarker-responsive probes for improved disease diagnosis.

## Supplementary Materials

Supplementary Materials Experimental materials and methods, compounds synthesis, supplementary figures, NMR spectra, and MS spectra are shown as Supplementary Figure S31-74, which are available in Supplementary Materials.
