# Supplementary material for: Responsive Trimodal Probes for In Vivo Imaging of Liver Inflammation by Coassembly and GSH-Driven Disassembly
Source: Research (Wash D C). 2020 Aug 28;2020:4087069. doi: 10.34133/2020/4087069 (PMC7520820; doi:10.34133/2020/4087069)

Title

Responsive Trimodal Probes for in Vivo Imaging of Liver Inflammation by Co-assembly and GSH-driven Disassembly

**Authors**

Yuxuan Hu, ^1,†^ Yuqi Wang, ^1,†^ Xidan Wen, ^1^ Yifan Pan, ^2^ Xiaoyang Chen, ^1^ Ruibing An, ^1^ Guandao Gao, ^2^ Hong-Yuan Chen, ^1^ and Deju Ye^1,^*

**Affiliations**

*^1^ State Key Laboratory of Analytical Chemistry for Life Science, Chemistry and Biomedicine Innovation Center (ChemBIC), School of Chemistry and Chemical Engineering, Nanjing University, Nanjing, 210023, China*

*^2^State Key Laboratory of Pollution Control and Resource Reuse, School of Environment, Nanjing University, Nanjing 210023, China*

Correspondence should be addressed to Deju Ye; e-mail: dejuye@nju.edu.cn

**Table of contents**

Materials and Instruments……………………………………………………………S3

Experimental methods……………………………………………………………...S4

Chemical Synthesis of **1-Gd**, **1-Gal**, **1-Gd-ctrl** and **1-Gal-ctrl**……………………S15

Supplementary Figures S1-30………………………………………………………S26

Supplementary Figures S31-74: NMR and HMRS Spectra………………………...S43

**Materials**

All chemicals were purchased from commercial sources (such as Aldrich, Adamas). High glucose Dulbecco’s Modified Eagle’s Medium (DMEM), fetal bovine serum (FBS), penicillin/streptomycin were purchased from Thermo (Shanghai, China). 3-(4,5-Dimethylthiazol-2-yl)-2,5-diphenyltetrazolium bromide (MTT) kit was obtained from KeyGen Biotech. Co. Ltd. (Nanjing, China). GSSG/GSH Quantification Kit was obtained from Dojindo, Co., Ltd. (Shanghai China)

**Instruments**

The ^1^H and ^13^C NMR spectra were acquired on a 400 MHz Bruker Avance III 400 spectrometer. High-performance liquid chromatography (HPLC) was carried out on Thermo Scientific Dionex Ultimate 3000 with CH_3_CN/H_2_O (1‰ CF_3_COOH) as the eluents. The UV-Vis spectra were carried out on an Ocean Optics Maya 2000 Pro spectrometer. The fluorescence spectra were measured with a HORIBA Jobin Yvon Fluoromax-4 fluorometer. Dynamic light scattering (DLS) analysis was measured using a 90 Plus/BI-MAS equipment (Brookhaven, USA). TEM images were obtained on a JEM-1011 transmission electron microscope (JEOL, Ltd., Japan) with an accelerating voltage of 100 kV. Atomic force microscope (AFM) images were acquired in tapping mode on a Bruker ICON AFM, and the diameter analysis was performed with NanoScope Analysis 1.5. MR *r*_1_ relaxivities were acquired on a 0.5 T MR scanner (NMI20−015 V−I, NIUMAG) and *in vitro* and *in vivo* MR imaging experiments were performed on a 1 T MR scanner (Bruker ICON™). Fluorescent images of cells and tissue slices were acquired on an Olympus IX73 fluorescent inverted microscope or a Leica TCS SP8 confocal laser scanning microscope. MTT assay was performed on a microplate reader (Tcan). Inductively coupled plasma mass spectrometry (ICP-MS) analysis were acquired with a Optima 5300DV plasma spectrum mass spectrometer (PE, America).

**Experimental Methods**

**Characterization of the self-assembly property of the template probe**

Probe **1-a**, **1-b**, and **1-c** were dissolved in DMSO to prepare stock solutions. The concentrations were determined based on the UV absorption of amino oxyluciferin fluorophore. Probe **1-a**, **1-b**, and **1-c** were diluted to 1 mL PBS buffer (pH 7.4) under sonication with final concentration of 200 μM. DLS analysis of each solution was performed using a 90 Plus/BI-MAS analyzer (Brookhaven, USA). For TEM analysis, the solutions were dropped onto a carbon-coated copper grid, followed by freeze-drying immediately. The samples were then examined on the JEM-1011 TEM. For ^19^F-MRS measurement, probe **1-a**, **1-b** or **1-c** (200 μM) in 0.5 mL PBS buffer was added with 10% D_2_O and 1mM NaTFA as internal standard. The solutions were transferred to NMR tubes, and the ^19^F NMR spectra for each solution was acquired on a 400 MHz Bruker Avance III 400 spectrometer.

**Co-assembly of probe 1-Gd and 1-Gal at different mole ratios**

Probe **1-Gd** or **1-Gal** were dissolved in DMSO to prepare stock solutions. The concentrations were determined based on the UV absorption of amino oxyluciferin fluorophore. Probes **1-Gd** and **1-Gal** at different mole ratios were dissolved into DMSO and mixed at a concentration of 20 mM, respectively. Then, the above DMSO solutions were slowly added into a PBS buffer (pH 7.4) under ultrasonication with a frequency of 40 kHz. After being kept under sonication for another 10 min, a series of uniform NPs were prepared with the final concentration of 200 μM (based on amino oxyluciferin fluorophore). DLS analysis of each solution was performed using a 90 Plus/BI-MAS analyzer (Brookhaven, USA). For TEM analysis of **GdNPs-Gal** at a mole radio of 5/1 (**1-Gd**/**1-Gal**), a drop of the solution was pipetted onto a carbon-coated copper grid, followed by freeze-drying immediately. The sample was then examined on the JEM-1011 TEM.

**Characterization of disassembly process in solutions**

A solution of **GdNPs-Gal** (200 μM) in 1mL PBS buffer was incubated with GSH (10 mM) at 37 ºC. DLS analysis was performed at 0, 10, 20, 30, 40, 50 and 60 min, using a 90 Plus/BI-MAS equipment (Brookhaven, USA). For comparison, a solution of **GdNPs-Gal-ctrl** (200 μM) in 1mL PBS buffer was incubated with GSH (10 mM) at 37 ºC. DLS analysis was performed at 0, 10, 20, 30, 40, 50 and 60 min, using a 90 Plus/BI-MAS equipment (Brookhaven, USA).

For HPLC analysis, a solution of **GdNPs-Gal** (200 μM) in PBS buffer was incubated with GSH (10 mM) at 37 ºC. At 0, 10, 20, 30, 40, 50, and 60 minutes, 100 μL of the solution was taken out and added into 1 mL of HCl (1 mM) immediately to stop the reaction. The solutions were injected into an HPLC system for analysis, respectively. **GdNPs-Gal-ctrl** (200 μM) in PBS buffer was incubated with GSH (10 mM) at 37 ºC for 1 h. 100 μL of the solution was taken out and injected into an HPLC system for analysis.

For fluorescence measurement, **GdNPs-Gal** (200 μM) in PBS buffer was incubated with GSH (10 mM) at 37 ºC. At 0, 2, 5, 10, 15, 20, 30, 40, 50, and 60 minutes, 100 μL of the solution was taken out and added into 900 μL PBS buffer. The fluorescence spectra were then recorded on a HORIBA Jobin Yvon Fluoromax-4 fluorometer, with an excitation at 405 nm. **GdNPs-Gal-ctrl** (200 μM) in PBS buffer was incubated with GSH (10 mM) at 37 ºC for 1 h. 100 μL of the solution was taken out and added into 900 μL PBS buffer and the fluorescence spectra were then recorded.

For *T*_1_ value measurement, **GdNPs-Gal** (200 μM) in PBS buffer was incubated with GSH (10 mM) at 37 ºC. *T*_1_ values were measured at 0, 10, 20, 30, 40, 50, and 60 minutes on a 0.5 T MR scanner (NMI20-015 V-I, NIUMAG), using a series of inversion-prepared fast spin-echo scans.

For ^19^F MRS, **GdNPs-Gal** (200 μM) in PBS buffer (containing 10% D_2_O and 1 mM NaTFA) was incubated with GSH (10 mM) at 37 ºC. At 0, 2, 5, 10, 15, 20, 30, 40, 50, and 60 minutes, 500 μL solutions were taken out and then added with 10 μL concentrated HCl. The solutions transferred to NMR tubes, and the ^19^F NMR spectra were acquired on a 400 MHz Bruker Avance III 400 spectrometer with the number of accumulation (NA) of 256.

**Evaluation of the Stability of GdNPs-Gal.**

To evaluate the stability of **GdNPs-Gal** under physiological conditions, a solution of **GdNPs-Gal** (200 μM, 1 mL) in PBS buffer or PBS buffer containing 10% FBS was kept at room temperature for over one week. The sizes, fluorescence spectra and *T*_1_ values of these two solutions were monitored every day. To evaluated the effect of pH on the stability, **GdNPs-Gal** (200 μM) were incubated in PBS buffer at different pH (4.0, 5.0, 6.0, 7.0, 8.0 and 9.0) for one week. The hydrodynamic size of **GdNPs-Gal** in each solution was monitored by DLS.

**Measurement of the longitudinal *r*_1_ relaxivity**

**GdNPs-Gal** (200 μM) or **GdNPs-Gal-ctrl** (200 μM) in PBS buffer (pH 7.4) was incubated with or without GSH (10 mM) at 37 ºC for 1 h. Then, the solutions were diluted in PBS buffer to prepare solutions with different concentrations (0.048, 0.069, 0.098, 0.14 and 0.2 mM of Gd^3+^). The T_1_ value (0.5 T) in each solution was acquired on a 0.5 T MR scanner (NMI20-015 V-I, NIUMAG), using a series of inversion-prepared fast spin-echo scans. Relaxation rates (R_1_) were determined as 1/T_1_, and the exact concentrations of Gd^3+^ were determined by ICP-MS. The longitudinal molar relaxivities (*r*_1_, unit of mM^−1^ s^−1^) were calculated based on the slope of R_1_ vs [Gd]. The T_1_-FLASH images (repetition time ms/echo time ms, 100/5; slice thickness 1.500 mm) of Dotarem (200 μM), **GdNPs-Gal** (200 μM) upon incubation with or without GSH (10 mM) in PBS buffer at 37 ºC for 1 h were acquired on a 1.0 T small animal MR scanner (Bruker I-CON).

Determination of the sensitivity of GdNPs-Gal towards GSH by MRI

To evaluate to sensitivity of GdNPs-Gal towards GSH, GdNPs-Gal (200 μM) was incubated with varying concentration of GSH (0, 0.25, 0.5, 1, 2.5, 5 and 10 mM) in PBS buffer at 37 ºC for 1 h, and the T_1_ value (0.5 T) of each solution was acquired on a 0.5 T MR scanner (NMI20-015 V-I, NIUMAG). The resulting T_1_ values were plotted to the concentration of GSH, and a linear regression fitted from 0.25-2.5 mM GSH was obtained, affording the slope *k*. The detection limit was calculated from 3σ/k, where σ represents the standard deviation of 11 blank measurements.

Determination of the sensitivity of GdNPs-Gal towards GSH by FL

To evaluate to sensitivity of GdNPs-Gal towards GSH by FL, GdNPs-Gal (200 μM) was incubated with varying concentration of GSH (0, 0.001, 0.0025, 0.005, 0.0075, 0.01, 0.025, 0.05, 0.075, 0.1, 0.25, 0.5, 0.75, 1, 2.5, 5 and 10 mM) in PBS buffer at 37 ºC for 1 h. 100 μL of the solution was taken out and added into 900 μL PBS buffer. The fluorescence spectra were then recorded on a HORIBA Jobin Yvon Fluoromax-4 fluorometer, with an excitation at 405 nm. The resulting fluorescence intensities (λ_em_ = 535 nm) were plotted to the concentration of GSH, and a linear regression fitted from 0 to 500 μM GSH was obtained, affording the slope k. The detection limit was calculated from 3σ/k, where σ represents the standard deviation of 11 blank measurements.

Determination of the sensitivity of GdNPs-Gal towards GSH by ^19^F MRS

To evaluate to sensitivity of GdNPs-Gal towards GSH by ^19^F MRS, GdNPs-Gal (200 μM) was incubated with varying concentration of GSH (0, 0.25, 0.5, 1, 2.5, 5, 7.5 and 10 mM) in PBS buffer (containing 10% D_2_O and 1 mM NaTFA) at 37 ºC for 1 h. The solutions were added with 10 μL concentrated HCl and transferred to NMR tubes immediately. The ^19^F NMR spectra were acquired on a 400 MHz Bruker Avance III 400 spectrometer with the number of accumulation (NA) of 256. The resulting S/N of ^19^F MRS signals were plotted to the concentration of GSH, and a linear regression fitted from 1 to 10 mM GSH was obtained, affording the slope k. The detection limit was calculated from 3σ/k, where σ represents the standard deviation of 11 blank measurements.

Determination of the Specificity of GdNPs-Gal towards GSH

The specificity towards GSH in vitro was evaluated based on two modalities of MRI and fluorescence, respectively. First, GdNPs-Gal (200 μM) was incubated with GSH (10 mM), Cys (10 mM), Hcy (10 mM), VC (10 mM), β-NADPH (10 mM), GSSG (10 mM), BSA (10 mg/mL), Cu^2+^, Na^+^, Mg^2+^, Zn^2+^. The solutions were kept at 37 ºC for 1 h. The T_1_ value (0.5 T) of each solution was acquired by a series of inversion-prepared fast spin-echo scans on a 0.5 T MR scanner (NMI20-015 V-I, NIUMAG), and the *T*_1_-weighted spin-echo images of solutions were acquired at a 1 T MR scanner (Bruker ICON™), using a *T*_1_-FLASH sequence (repetition time ms/echo time ms, 100/5; slice thickness 1.500 mm). Second, GdNPs-Gal (200 μM) was incubated with GSH (10 mM), Cys (10 mM), Hcy (10 mM), VC (10 mM), β-NADPH (10 mM), GSSG (10 mM), BSA (10 mg/mL), Cu^2+^, Na^+^, Mg^2+^, Zn^2+^. The solutions were kept at 37 ºC for 1 h and diluted to 20 μM in PBS buffer. The fluorescence spectra were then acquired with an excitation at 405 nm.

Cell culture

Human cervical cancer HeLa cells, liver hepatocellular carcinoma HepG2 cells and human umbilical vein endothelial HUVEC cells were cultured in DMEM (Dulbecco’s Modified Eagle Medium) medium. The mediums were supplemented with 10% (v/v) fetal bovine serum (FBS), 100 units per mL penicillin, and 100 units per mL streptomycin. All cells were cultured at 37 ºC in a humidified atmosphere (5% CO_2_).

Cytotoxicity assay (MTT)

The HepG2 cells were seeded in flat-bottomed 96-well plates (5000 cells per well) and incubated at 37 ºC overnight. Varying concentration of 1-GdNPs, 1-GalNPs and GdNPs-Gal (0, 1, 5, 10, 25, 50, 75, 100, 150, and 200 μM) in DMEM medium (100 μL) were added. After incubation for 24 h, 50 μL MTT solution (1 mg /mL in PBS) was then added into each well. The cells were kept at 37 ºC for 4 h, and the solution in each well was then removed carefully. The resulting purple formazan crystals in the wells were dissolved by addition of 150 μL DMSO. The absorbance (OD) at 490 nm in each well was acquired on a microplate reader (Tcan). The absorbance of blank cells (OD control) were used as the control, and the percentage of cell viability in each treatment was calculated by dividing OD to OD control.

Trimodal detection of GSH in cell lysates.

Approximately 1×10^7^ HepG2 cells were seeded onto 10 cm cell culture dishes and allowed to grow at 37 ºC overnight. The trypsin (1 mL/well) was added into each well, maintained at 37 ºC for 3 min to detach the cells. The cells were then collected in a 1.5 mL tube after centrifugation at 1000 rpm for 4 min. The cell pellets were washed with PBS twice and the supernatants were removed after centrifugation. The cell pellets were then added with 200 μL PBS buffer and sonicated for 10 minutes in an ice bath. After sonication, the solutions were centrifuged at 8000g at 4 ºC for 10 min. The supernatant was collected and kept on ice. GdNPs-Gal or GdNPs-Gal-ctrl at a final concentration of 200 μM was added to respective 100 μL cell lysates, and the mixtures were incubated at 37 ºC for 4 h. The fluorescence spectra were recorded on a HORIBA Jobin Yvon Fluoromax-4 fluorometer, with an excitation at 405 nm. The *T*_1_ values of the solutions were acquired on a 0.5 T MR scanner (NMI20-015 V-I, NIUMAG), and the ^19^F NMR spectra were acquired on a 400 MHz Bruker Avance III 400 spectrometer with the number of accumulation (NA) of 256.

General procedure for fluorescence imaging of cells

Cells (~ 5×10^4^) were seeded onto a glass-bottom dish (In Vitro Scientific, D35-20-1-N) and allowed to grow overnight. GdNPs-Gal, 1-GdNPs or GdNPs-Gal-ctrl (200 μM) in DMEM was added into dishes and incubated at 37 ºC for 4 h. To scavenge the concentration of endogenous GSH in cells, HepG2 cells were pretreated with NEM (100 μM) for 30 min, and then incubated with GdNPs-Gal (200 μM) for another 4 h. The medium was removed, washed with PBS buffer three times. After adding fresh medium, fluorescence images were acquired on an Olympus IX73 fluorescent inverted microscope with the excitation filter of 350-370 nm and an emission filter of 535 ± 25 nm. Each experiment was repeated for three times. The cell images were then analyzed using the ImageJ software (NIH).

Colocalization Study

To examine the intracellular location, HepG2 cells were incubated with GdNPs-Gal (200 μM) for 0.5 h and 4 h. After being washed with PBS for three times, the cells were then incubated with 200 nM Lyso Traker Red DND-99 for 20 min. The medium was removed, washed with PBS buffer three times. After adding fresh medium, the fluorescence images of cells were captured on a Leica TCS SP8 confocal laser scanning microscope. For GdNPs-Gal, the excitation wavelength was 405 nm, and the emission wavelength was from 525 nm to 550 nm. For Lyso Traker Red, the excitation wavelength was 577 nm, and the emission wavelength was from 580 nm to 600 nm.

Detection of GSH in cell pellets using ^1^H-MRI, FL and ^19^F-MRS

HepG2 cells were seeded on 10 cm cell culture dishes at a density of 1×10^7^ cells and allowed to grow overnight. GdNPs-Gal or GdNPs-Gal-ctrl (200 μM) in DMEM was added into dishes and incubated at 37 ºC for 4 h. To decrease the concentration of GSH in cells, cells were pretreated with NEM (100 μM) for 30 min, and then incubated with GdNPs-Gal (200 μM) for another 4 h. After being washed with PBS for three times, trypsin (1 mL) was added into each dish and maintained at 37 ºC for 3 min to detach the cells. The cell pellets were then collected into 200 μL tubes and centrifuged at 1000 rpm for 4 min. The MR imaging and fluorescence imaging of cell pellets were then acquired on a 1 T MR scanner (repetition time ms/echo time ms, 446/15; slice thickness 1.000 mm) and an IVIS Lumina XR III system (Ex/Em = 420/540 nm). For ^19^F-MRS measurement, 450 μL PBS buffer was added, and the cell pellets were sonicated for 10 minutes in an ice bath, the solution was added with 10%(v/v) D_2_O and 1 mM NaTFA as internal standard. The ^19^F NMR spectra were acquired on a 400 MHz Bruker Avance III 400 spectrometer with the number of accumulation (NA) of 256. After that, the solutions were dried and digested with concentrated HNO_3_ solution under heating at 120 ºC for 24 h, respectively. The residues were then diluted with 4 mL 2% HNO_3_ solution and the concentration of Gd^3+^ were determined by ICP-MS, which were further applied to calculate the contents of intracellular Gd, respectively.

**Quantitative analysis of the content of Gd(III) in cells**

Approximately 1×10^7^ HepG2 cells were seeded onto 10 cm cell culture dishes. After being incubation at 37 ºC overnight. The medium was replaced with 3 mL fresh DMEM containing probe 1-Gd or 1-Gal at different mole ratios with final concentration of 200 μM (based on amino oxyluciferin fluorophore) and the cells were incubated at 37 ºC for another 4 h. The culture DMEM medium in each dish was removed, and cells were washed with PBS buffer three times. Then, the cells were trypsinized, collected, and the cell numbers were counted. The collected cell pellets were digested with concentrated HNO_3_ solution under heating at 120 ºC for 24 h, respectively. The residues were then diluted with 4 mL 2% HNO_3_ solution and the concentration of Gd(III) were determined by ICP-MS, which were further applied to calculate the contents of intracellular Gd, respectively.

**Animal Models**

BALB/c female mice at 6-8 weeks’ old were purchased from the Model Animal Research Center (MARC) of Nanjing University (Nanjing, China) and used according to the regulations of the Institutional Animal Care and Use Committee (IACUC) of Nanjing University.

To establish liver inflammation model, a solution of LPS (20 mg kg^-1^) was i.p. injected into mice. The inflammatory response was further confirmed by blood tests and H&E staining.

**Measurement of blood circulation time**

GdNPs-Gal (500 μM in 200 μL saline) was i.v. injected into heathy female mice (6-8 weeks old). At 0, 15 min, 0.5, 1, 2, 4, 8, 12, and 24 h, bloods were collected from the venous sinus of mice (three for each time point), and preserved into 1.5-mL EDTA coated Eppendorf tubes that are chilled on ice. The collected bloods were digested with concentrated HNO_3_ under 120 ºC for 24 h. The residues were then diluted with 6 mL 2% HNO_3_ solution, and the concentrations of Gd(III) were determined by ICP-MS, which were further applied to calculate the contents of Gd(III) in blood. Plot of the percentage of Gd(III) remained in the blood versus injection time to determine the blood circulation half-time (*t*_1/2_).

**Biodistribution studies**

Heathy female mice were i.v. injected with GdNPs-Gal or GdNPs-Gal-ctrl (0.1 mmol kg^-1^ Gd) in 200 μL saline (n = 3 per group). After 4 h, the mice were sacrificed, and major organs including liver, kidneys, intestines, heart, lung, stomach and spleen were resected and weighed. The tissues were cut into small pieces, and digested with concentrated HNO_3_ under 120 ºC for 24 h. The residue in each organ was then diluted with 6 mL 2% HNO_3_ solution, and the concentration of Gd(III) was determined by ICP-MS. The %ID/g was also calculated for comparison.

**Immunohistochemistry studies and GSH** quantification

To evaluate the establishment of liver inflammation, mice with and without treatment of LPS were sacrificed. Blood was collected and the cytokines (TNF-α and IL-1β) were analysis using a standard protocol with ELASA kits. Parts of liver tissues were fixed in 4% formalin and then embedded in paraffin before 10-µm sectioning. Histology samples were stained by H&E using a standard protocol. White light images were acquired using an IX73 optical microscope equipped with a color camera. The remaining liver tissues were weighed, ground and sonicated in an ice bath after addition of cold PBS buffer. The mixtures were centrifuged at 15000g at 0 ºC for 15 min and the supernatant was collected and kept on ice. GSH quantification in the liver tissue lysate was performed using GSSG/GSH Quantification Kit (Dojindo, Co., Ltd.).

MR imaging of liver tissue in mice

For non-invasive MR imaging of GSH concentration in liver tissue of normal mice, mice were i.v. injected with GdNPs-Gal or GdNPs-Gal-ctrl (0.1 mmol kg^-1^) in 200 μL saline. The *T*_1_-weighted MR images were acquired before injection, 1 h, 2 h, 4 h, 6 h and 8 h post injection.

For *T*_1_-weighted MR imaging of GSH concentration in liver tissue of hepatitis mice, mice were i.p. injected with LPS (20 mg kg^-1^) to establish the liver inflammation model. After 6 h, mice were i.v. injected with GdNPs-Gal (0.1 mmol kg^-1^) in 200 μL saline. The MR images were acquired before injection, 1 h, 2 h, 4 h, 6 h and 8 h post injection.

All the MR imaging experiments were conducted on a 1.0 T small animal MR scanner (Bruker I-CON). The mice were scanned in the tail-first prone position. The images were acquired using a *T*_1_-RARE imaging sequence. The detailed MR imaging parameters were as follows: repetition time ms/echo time ms, 446/15.0; image size, 256 × 256; slice thickness 1.000 mm. The acquisition time for each MR imaging was 11 min 25 s.

Each experiment was conducted in three mice. Acquired MRI data were then transferred as DICOM images to a RadiAnt DICOM Viewer for quantitative image analysis. This consisted of manual segmentation of the liver ROI for each slice, slice-wise normalization of mean liver signal intensity with the 1 mM Dotarem reference standard to account for inter-session variability, followed by combining these normalized, slice-wise values to generate mean volumetric liver signal intensities (SI) for each time point. Percentage signal enhancement (% SE) was calculated at each time point as the % difference between the liver SI in the precontrast data set:

% SE (t) = (SI (t) - SI (t=0))/SI (t=0); for each mouse, for every session

Fluorescence Imaging of Liver Slices

For fluorescence imaging of liver tissue slices, mice with and without pretreated of LPS were i.v. injected with GdNPs-Gal (0.1 mmol kg^-1^) in 200 μL saline. After 4 h, the mice were sacrificed and the livers were resected. The isolated liver tissues were cut using a vibrating-blade microtome to obtain 10 µm-thickness slices. The images of tumor tissue slices were acquired with the Olympus IX73 fluorescent inverted microscope.

^19^F MRS Measurement of Liver tissue lysates

For ^19^F MRS measurement of liver tissue slices, mice with and without pretreated of LPS were i.v. injected with GdNPs-Gal (0.1 mmol kg^-1^) in 200 μL saline. After 4 h, the mice were sacrificed and the livers were resected. The isolated liver tissues were weighed, ground and sonicated in an ice bath after addition of cold PBS buffer. The mixtures were added with 10% (v/v) D_2_O and 1mM NaTFA as internal standard. The ^19^F NMR spectra were acquired on a 400 MHz Bruker Avance III 400 spectrometer with the number of accumulation (NA) of 256.

**MR imaging of GSH concentration in hepatitis recovery**

Female mice were randomly divided into groups with three mice per group. Before establishing liver inflammation model, healthy mice were i.v. injected with GdNPs-Gal (0.1 mmol kg^-1^) in 200 μL saline and Axial *T*_1_ -weight MR images of livers were acquired before and 4 h post injection. For hepatitis recovery models, mice were treated with LPS (20 mg kg^-1^, 100 μL saline) via i.p. injection at 1^st^ day. After 10 h on the 1^st^, 2^nd^ and 3^rd^ day, mice were administered with DEX (5 mg kg^-1^, 100 μL saline) via i.p. injection. For MR imaging, mice were i.v. injected with GdNPs-Gal (0.1 mmol kg^-1^) in 200 μL saline 6 h after LPS treatment on the 1^st^, 3^rd^, 5^th^, and 7^th^ day. Axial *T*_1_ -weight MR images of livers were acquired before and 4 h post injection. For GSH quantification, mice were treated with LPS (20 mg kg^-1^, 100 μL saline) via i.p. injection. After 10 h on the 1^st^, 2^nd^ and 3^rd^ day, mice were administered with DEX (5 mg kg^-1^, 100 μL saline) via i.p. injection. Mice were sacrificed 6 h after LPS treatment on the 1^st^, 3^rd^, 5^th^, and 7^th^ day. The liver tissues were taken out, weighed, and sonicated in an ice bath after addition of cold PBS buffer. The mixtures were centrifuged at 15000g at 0 ºC for 15 min and the supernatant was collected and kept on ice. GSH quantification in the liver lysate was performed using GSSG/GSH Quantification Kit (Dojindo, Co., Ltd.).

Evaluation of in vivo clearance

Three healthy female mice were i.v. injected of GdNPs-Gal (0.1 mmol kg^-1^) in 200 μL saline. Feces and urine were collected at the period of 0-4 h, 4-8 h, 8-12 h, 12-24 h, 24-48 h and 48-72 h after injection. The collected Feces and urine were digested with concentrated HNO_3_ solution under 120 ºC for 24 h, and the residues were then diluted with 6 mL 2% HNO_3_ solution. The concentration of Gd(III) was determined by ICP-MS and further applied to calculate the clearance of **GdNPs-Gal**.

S**tatistical Analysis**

Results are expressed as the mean ± standard deviation unless otherwise stated. Statistical comparison between two groups were determined by Student’s t test. P < 0.05 was considered statistically significant. All statistical calculations were performed using GraphPad Prism 6 (GraphPad Software Inc., CA, USA).

**Chemical synthesis and characterization of probes**

**Scheme S1.** Synthesis of probe **1-Gd**. Reaction conditions: (a) (i) Triphosgene, DMAP, 120 ºC toluene, 3 h; (ii) DCM, overnight, 65%; (b) (i) Cysteine, DCM, DIPEA, 2 h; (ii) MnO_2_, overnight, 60%; (c) TFA, DCM, 3 h, 90%; (d) MeOH, 1 h, 58%; (e) (i) HBTU, BocNH-PEG_4_-CH_2_CH_2_NH_2_, 3 h; (ii) TFA, 1 h, 76%; (f) (i) DOTA(OtBu), HBTU, DIPEA,3 h; (ii) TFA, 3 h, 80%; (g) GdCl_3_, NaHCO_3_, pH 7.0, overnight, 52%.

*Synthesis of compound* **2**: CBT-NH_2_ (220 mg, 1.25 mmol) and DMAP (152 mg, 1.25 mmol) were dissolved in 50 mL dry toluene at 120 ºC under N_2_. The reaction mixture was then cooled to room temperature and stirred at 0 ºC for 20 min. Triphosgene (370 mg, 1.25 mmol) was dissolved in 5 mL dry toluene, and the solution was added dropwise to the reaction mixture at 0 ºC under N_2_. The solution was then kept stirring at 120 ºC for another 3 h. The toluene was rapidly removed under vacuum and the residues were dissolved in 15 mL dry DCM. Compound **1** (320 mg, 1.0 mmol) was dissolved in 5 mL dry DCM and added dropwise to the reaction mixture at 0 ºC. The reaction was continued at room temperature overnight. The solvent was removed and purified by flash chromatography on silica gel to give the desired product as a white solid. Yield: 338.6 mg (65%). ^1^H NMR (400 MHz, DMSO-*d*_6_) *δ* 10.28 (s, 1H), 8.49 (d, *J* = 2.0 Hz, 1H), 8.16 (d, *J* = 9.0 Hz, 1H), 7.66 (d, *J* = 11.2 Hz, 1H), 7.35 (d, *J* = 4.2 Hz, 12H), 7.26 (p, *J* = 4.3 Hz, 3H), 3.95 (t, *J* = 6.5 Hz, 2H), 2.48 (t, *J* = 6.5 Hz, 2H). ^13^C NMR (101 MHz, DMSO-*d*_6_) *δ* 152.93, 147.15, 144.19, 139.79, 136.85, 134.43, 129.04, 128.82, 128.09, 126.82, 124.78, 119.98, 113.57, 109.79, 66.25, 62.75, 40.16, 40.11, 39.95, 39.90, 39.70, 39.49, 39.28, 39.07, 38.86, 30.71. MS: calcd. For C_30_H_23_N_3_O_2_S_2_^+^ [M+Na]^+^: 544.1129; MALDI-MS found: m/z 543.8540.

*Synthesis of compound* **3**: Compound **2** (260.5 mg, 0.5 mmol), Cysteine (66 mg, 0.55 mmol) and DIPEA (129 mg, 1.0 mmol) were dissolved in DCM/MeOH (1:1, 10 mL). The solution was then kept stirring at room temperature for 2 h. MnO_2_ (530 mg, 5.0 mmol) was added into the reaction mixture and the reaction was continued at room temperature overnight. The solvent was removed and purified by flash chromatography on silica gel to give the desired product as a light yellow solid. Yield: 187 mg (60%). ^1^H NMR (400 MHz, DMSO-*d*_6_) *δ* 10.05 (s, 1H), 8.62 (s, 1H), 8.29 (d, *J* = 2.1 Hz, 1H), 7.97 (d, *J* = 8.9 Hz, 1H), 7.50 (dd, *J* = 8.9, 2.2 Hz, 1H), 7.28 (d, *J* = 4.2 Hz, 12H), 7.20 (q, *J* = 4.3 Hz, 3H), 3.87 (t, *J* = 6.5 Hz, 2H), 2.40 (t, *J* = 6.5 Hz, 2H). ^13^C NMR (101 MHz, DMSO-*d*_6_) *δ* 161.55, 161.05, 158.36, 153.00, 148.36, 148.30, 144.20, 138.03, 136.03, 131.46, 129.04, 128.09, 126.82, 123.56, 118.87, 110.24, 66.24, 62.61, 30.76. MS: calcd. For C_33_H_25_N_3_O_2_S_3_^+^ [M+H]^+^: 624.1007; HRMS found: m/z 624.1073.

*Synthesis of compound* **6**: Compound **3** (155.7 mg, 0.25 mmol) was dissolved in a solution consisting of DCM, TFA and TIPSH (v/v/v = 50/46/4, 10mL). The reaction mixture was then stirred at room temperature for 3 h. The solvent was rapidly removed under vacuum and the residues were dissolved in 10 mL MeOH. Compound **5** (127 mg, 0.3 mmol) was dissolved in 5 mL MeOH and the solution was rapidly added into the reaction mixture. The reaction was then continued at room temperature for 1 h, MeOH was removed and the residues were purified by flash chromatography on silica gel to afford compound **6** as a light yellow solid. Yield: 101 mg (58%). ^1^H NMR (400 MHz, DMSO-*d*_6_) *δ* 10.18 (s, 1H), 9.19 (t, *J* = 5.4 Hz, 1H), 8.70 (s, 1H), 8.49 (s, 2H), 8.38 (s, 1H), 8.31 (s, 1H), 8.02 (d, *J* = 8.9 Hz, 1H), 7.58 (d, *J* = 10.8 Hz, 1H), 4.40 (t, *J* = 6.3 Hz, 2H), 3.64 (q, *J* = 6.4 Hz, 2H), 3.10 (t, *J* = 6.2 Hz, 2H), 2.99 (t, *J* = 6.7 Hz, 2H). ^13^C NMR (101 MHz, DMSO-*d*_6_) *δ* 163.81, 162.04, 161.53, 158.82, 153.71, 148.87, 148.79, 138.55, 136.77, 136.49, 131.94, 131.10 (q, *J* = 33 Hz), 128.45, 128.43, 125.38, 124.91, 124.02, 122.20, 119.39, 110.76, 62.81, 37.21, 37.15. MS: calcd. For C_25_H_18_F_6_N_4_O_5_S_4_^+^ [M+H]^+^: 697.0064; HRMS found: m/z 697.0157.

*Synthesis of probe* **1-c**: Compound **6** (70 mg, 0.1 mmol), 5,8,11,14-Tetraoxa-2-azahexadecanoic acid,16-amino-,1,1-dimethyl ester (50 mg, 0.15 mmol), HBTU (57 mg, 0.15 mmol) and DIPEA (32 mg, 0.25mmol) were dissolved in 5 mL dry THF. The solution was then kept stirring at room temperature for 3 h. The solvent was removed and purified by flash chromatography on silica gel to give a light yellow solid. The solid was then dissolved in a solution consisting of DCM and TFA (v/v = 70/30, 5mL). The reaction mixture was then stirred at room temperature for 1 h. The solvent was removed under vacuum to give desired product as a light yellow solid. Yield: 69 mg (76%). ^1^H NMR (400 MHz, DMSO-*d*_6_) *δ* 10.19 (s, 1H), 9.23 (t, *J* = 5.4 Hz, 1H), 8.52 (s, 1H), 8.49 (s, 2H), 8.46 (t, *J* = 5.7 Hz, 1H), 8.41 (s, 1H), 8.31 (s, 1H), 8.03 (d, *J* = 8.9 Hz, 1H), 7.56 (dd, *J* = 9.0, 1.6 Hz, 1H), 4.40 (t, *J* = 6.2 Hz, 2H), 3.65 (q, *J* = 6.4 Hz, 2H), 3.54 (ddd, *J* = 21.3, 12.6, 5.5 Hz, 18H), 3.10 (t, *J* = 6.2 Hz, 2H), 2.98 (dt, *J* = 10.8, 6.1 Hz, 4H). ^13^C NMR (101 MHz, DMSO-*d*_6_) *δ* 163.82, 161.32, 160.43, 158.56, 153.73, 151.54, 148.76, 138.57, 136.78, 136.54, 131.11 (q, *J* = 33 Hz), 128.46, 127.60, 125.40, 124.91, 124.04, 122.20, 119.48, 110.64, 70.24, 70.21, 70.11, 70.07, 70.02, 69.24, 67.12, 62.82, 39.13, 39.06, 37.21, 37.14. MS: calcd. For C_35_H_40_F_6_N_6_O_8_S_4_^+^ [M+H]^+^: 915.1695; HRMS found: m/z 915.1884.

*Synthesis of compound* **7**: **1-c** (50 mg, 0.05mmol), 1,4,7,10-Tetraazacyclododecane-1,4,7-tris-tert-butyl acetate-10-acetic acid (28 mg, 0.05 mmol), HBTU (19 mg, 0.05 mmol) and DIPEA (20 mg, 0.15mmol) were dissolved in 2 mL dry THF. The solution was then kept stirring at room temperature for another 3 h. The solvent was rapidly removed under vacuum and the residues were dissolved in a solution consisting of DCM and TFA (v/v = 5/95, 5mL). The reaction mixture was then stirred at room temperature for another 3 h. The solvent was removed under vacuum to give compound **7** as light yellow solid, which was directly used for next step without further purification. ^1^H NMR (400 MHz, DMSO-*d*_6_) *δ* 10.18 (s, 1H), 9.25 (t, *J* = 5.4 Hz, 1H), 8.60 (s, 1H), 8.51 (s, 1H), 8.49 (s, 2H), 8.46 (t, *J* = 5.9 Hz, 1H), 8.42 – 8.37 (m, 1H), 8.29 (s, 1H), 8.02 (d, *J* = 8.9 Hz, 1H), 7.57 (dd, *J* = 9.0, 2.0 Hz, 1H), 4.39 (t, *J* = 6.2 Hz, 2H), 4.00 (m, 8H), 3.66 – 3.62 (m, 2H), 3.61 – 3.47 (m, 18H), 3.46 – 3.34 (m, 8H), 3.31 – 3.21 (m, 4H), 3.16 – 3.04 (m, 8H), 3.00 (t, *J* = 6.7 Hz, 2H). ^13^C NMR (101 MHz, DMSO-*d*_6_) *δ* 172.17, 163.83, 161.32, 160.44, 158.80 (q, *J* = 34 Hz, CF_3_COOH), 158.56, 153.75, 151.53, 148.77, 138.57, 136.80, 136.54, 131.11 (q, *J* = 33 Hz), 128.46, 127.56, 125.34, 124.90, 124.03, 122.19, 119.50, 118.43 (q, *J* = 294 Hz, CF_3_COOH), 110.67, 70.26, 70.22, 70.20, 70.15, 70.01, 69.21, 69.16, 62.81, 55.18, 54.36, 53.01, 50.98, 48.84, 48.46, 39.12, 37.21, 37.16. MS: calcd. For C_51_H_66_F_6_N_10_O_15_S_4_^+^ [M+H]^+^: 1301.3496; MALDI-MS found: m/z 1301.5131.

*Synthesis of probe* **1-Gd**: To a solution of compound **7** (65 mg, 0.05 mmol) in MeOH (2 ml) was carefully added a solution of 1 M NaHCO_3_ to adjust pH value to 7.0. Then, a solution of GdCl_3_ (185 mg, 0.5 mmol) in 1 mL water was added. After stirring at r.t. for 10 min, the pH value of the reaction solution was further adjusted to 7.0 using 1 M NaHCO_3_, and the reaction mixture was stirred at r.t. overnight. After reaction, the reaction mixture was centrifuged (4000 rpm, 5 min), and the supernatant was purified by HPLC to give probe **1-Gd** as a light yellow solid. Yield: 38 mg (52%). MS: calcd. For C_51_H_63_F_6_N_10_O_15_S_4_Gd^+^ [M+H]^+^: 1456.2502; HRMS found: m/z 1456.2682.

**Scheme S2.** Synthesis of probe **1-Gal**. Reaction conditions: (a) TMSOTf, 0 ºC overnight, 40%; (b) HBTU, Propyne-PEG_4_-NH_2_, 3 h, 90%; (c) (i) CH_3_ONa, CH_3_OH, 6 h; (ii) CuSO_4_, Sodium L-Ascorbate, 30 min, 60%;

*Synthesis of compound* **8**: 2,​ 3,​ 4,​ 6-​Tetra-​*O*-​acetyl-​D-​glucopyranosyl trichloroacetimidate (98.2mg, 0.2mmol) and 2-​[2-​[2-​(2-​Azidoethoxy)​ethoxy]​ethoxy]​ethanol (43.8 mg, 0.2mmol) were dissolved in 2 mL dry DCM under N_2_. The reaction mixture was then stirred at 0 ºC for 20 min. TMSOTf (22 mg, 0.1mmol) was dissolved in 0.5 mL dry DCM and the solution was added dropwise to the reaction mixture at 0 ºC under N_2_. The reaction was continued at room temperature overnight. The solvent was removed and purified by flash chromatography on silica gel to give the desired product as a colorless oil. Yield: 44 mg (40%). ^1^H NMR (400 MHz, DMSO-*d*_6_) *δ* 5.26 (d, *J* = 3.4 Hz, 1H), 5.15 (dd, *J* = 10.4, 3.5 Hz, 1H), 4.97 – 4.90 (m, 1H), 4.73 (d, *J* = 8.0 Hz, 1H), 4.19 (t, *J* = 6.3 Hz, 1H), 4.05 (d, *J* = 7.8 Hz, 2H), 3.85 – 3.74 (m, 1H), 3.65 – 3.58 (m, 3H), 3.59 – 3.45 (m, 10H), 3.45 – 3.30 (m, 2H), 2.12 (s, 3H), 2.02 (d, *J* = 4.5 Hz, 6H), 1.92 (s, 3H). ^13^C NMR (101 MHz, DMSO-*d*_6_) *δ* 170.39, 170.33, 169.95, 169.60, 100.48, 70.72, 70.33, 70.29, 70.27, 70.16, 69.87, 69.70, 69.10, 69.01, 67.82, 61.76, 50.47, 20.94, 20.92, 20.83, 20.79. MS: calcd. For C_22_H_35_N_3_O_13_^+^ [M+Na]^+^: 572.2068; MALDI-MS found: m/z 572.1011.

*Synthesis of compound* **10**: Compound **6** (70 mg, 0.1 mmol), Triethylene glycol 2-aminoethyl propargyl ether (35 mg, 0.15 mmol), HBTU (57 mg, 0.15 mmol) and DIPEA (32 mg, 0.25mmol) were dissolved in 5 mL dry THF. The solution was then kept stirring at room temperature for 3 h. The solvent was removed and purified by flash chromatography on silica gel to give a light yellow solid. Yield: 82 mg (90%). ^1^H NMR (400 MHz, DMSO-*d*_6_) *δ* 10.18 (s, 1H), 9.20 (t, *J* = 5.4 Hz, 1H), 8.52 (s, 1H), 8.49 (s, 2H), 8.45 (t, *J* = 5.8 Hz, 1H), 8.40 (s, 1H), 8.32 (s, 1H), 8.03 (d, *J* = 8.9 Hz, 1H), 7.56 (dd, *J* = 9.0, 1.8 Hz, 1H), 4.40 (t, *J* = 6.2 Hz, 2H), 4.12 (d, *J* = 2.3 Hz, 2H), 3.65 (q, *J* = 6.4 Hz, 2H), 3.64 – 3.42 (m, 16H), 3.41 (t, *J* = 2.3 Hz, 1H), 3.10 (t, *J* = 6.2 Hz, 2H), 2.99 (t, *J* = 6.7 Hz, 2H). ^13^C NMR (101 MHz, DMSO-*d*_6_) *δ* 163.82, 161.29, 160.39, 158.61, 153.73, 151.56, 148.78, 138.54, 136.79, 136.55, 131.11 (q, *J* = 33 Hz), 128.46, 127.56, 125.38, 124.92, 124.04, 122.20, 119.47, 80.76, 77.53, 70.23, 70.04, 69.94, 69.24, 68.94, 62.82, 57.92, 39.15, 37.22, 37.13. MS: calcd. For C_36_H_37_F_6_N_5_O_8_S_4_^+^ [M+H]^+^: 910.1429; HRMS found: m/z 910.1631.

*Synthesis of probe* **1-Gal**: Compound **8** (22 mg, 0.04 mmol) and CH_3_ONa (27 mg, 0.5 mmol) were dissolved in 1 mL CH_3_OH. The solution was then kept stirring at room temperature for 6 h. Dowex® 50WX8-400 ion-exchange resin was slowly added into the reaction mixture to adjust the pH to ~5-6. The mixture was filtered and the filtrate was collected. The solvent was removed and the residues were dissolved in 0.5 mL tert-Butanol. Compound **10** (18.8 mg, 0.02 mmol) was dissolved in the solution. CuSO_4_ (1.5 mg, 0.01 mmol) and Sodium L-Ascorbate (3.96 mg, 0.02 mmol) was dissolved in 0.2 mL H_2_O and added to the reaction mixture. The solution was kept stirring at r.t. for 30 min. After the reaction, the mixture was purified by HPLC to afford probe **1-Gal** after lyophilization. Yield: 15.5 mg (60%). ^1^H NMR (400 MHz, DMSO-*d*_6_) *δ* 10.17 (s, 1H), 9.20 (t, *J* = 5.5 Hz, 1H), 8.51 (s, 1H), 8.49 (s, 2H), 8.44 (t, *J* = 5.8 Hz, 1H), 8.40 (s, 1H), 8.30 (s, 1H), 8.04 (s, 1H), 8.02 (d, *J* = 9.0 Hz, 1H), 7.57 (dd, *J* = 9.0, 2.1 Hz, 1H), 4.54 – 4.47 (m, 4H), 4.40 (t, *J* = 6.3 Hz, 2H), 4.10 (d, *J* = 7.0 Hz, 1H), 3.81 (t, *J* = 5.3 Hz, 2H), 3.68 – 3.61 (m, 3H), 3.60 – 3.43 (m, 29H), 3.30 (dt, *J* = 19.2, 4.7 Hz, 3H), 3.10 (d, *J* = 12.5 Hz, 2H), 3.00 (t, *J* = 6.8 Hz, 2H). ^13^C NMR (101 MHz, DMSO-*d*_6_) *δ* 163.83, 161.29, 160.41, 158.88 (q, *J* = 35 Hz, CF_3_COOH), 158.59, 153.73, 151.56, 148.78, 144.23, 138.54, 136.80, 136.55, 131.11 (q, *J* = 33 Hz), 128.45, 127.53, 125.38, 124.91, 124.67, 124.02, 122.19, 119.47, 110.69, 110.68, 104.04, 99.98, 75.66, 73.93, 70.94, 70.26, 70.21, 70.13, 70.06, 70.03, 69.98, 69.40, 69.24, 69.15, 68.61, 68.16, 63.96, 62.83, 60.89, 49.76, 39.15, 37.24, 37.17. MS: calcd. For C_50_H_64_F_6_N_8_O_17_S_4_^+^ [M+H]^+^: 1291.3176; HRMS found: m/z 1291.396.

**Scheme S3.** Synthesis of probe **1-Gd-ctrl**. Reaction conditions: (a) (i) Triphosgene, DMAP, 120 ºC toluene, 3 h; (ii) DCM, overnight, 70%; (b) (i) Cysteine, DCM, DIPEA, 2 h; (ii) MnO_2_, overnight, 80%; (c) (i) HBTU, BocNH-PEG_4_-CH_2_CH_2_NH_2_,3 h; (ii) TFA, 1 h, 75%; (d) (i) DOTA(OtBu), HBTU, DIPEA,3 h; (ii) TFA, 3 h, 90%; (e) GdCl_3_, NaHCO_3_, pH 7.0, overnight, 56%.

*Synthesis of compound* **12**: CBT-NH_2_ (220 mg, 1.25 mmol) and DMAP (152 mg, 1.25 mmol) were dissolved in 50 mL dry toluene at 120 ºC under N_2_. The reaction mixture was then stirred at 0 ºC for 20 min. Triphosgene (370 mg, 1.25 mmol) was dissolved in 5 mL dry toluene, and the solution was added dropwise to the reaction mixture at 0 ºC under N_2_. The solution was then kept stirring at 120 ºC for another 3 h. The toluene was rapidly removed under vacuum and the residues were dissolved in 15 mL dry DCM. compound **11** (357 mg, 1.0 mmol) was dissolved in 5 mL dry DCM and added dropwise to the reaction mixture at 0 ºC. The reaction was continued at room temperature overnight. The solvent was removed and purified by flash chromatography on silica gel to give the desired product as a white solid. Yield: 390 mg (70%). ^1^H NMR (400 MHz, DMSO-*d*_6_) *δ* 10.21 (s, 1H), 8.97 (s, 1H), 8.50 (s, 3H), 8.30 (s, 1H), 8.15 (d, *J* = 9.0 Hz, 1H), 7.68 (dd, *J* = 9.1, 2.1 Hz, 1H), 4.15 (t, *J* = 6.6 Hz, 2H), 3.35 (s, 2H), 1.74 – 1.64 (m, 2H), 1.64 – 1.55 (m, 2H), 1.41 (m, 4H). ^13^C NMR (101 MHz, DMSO-*d*_6_) *δ* 163.49, 154.01, 147.53, 140.56, 137.33, 137.13, 134.70, 131.05 (q, *J* = 33 Hz), 128.43, 128.39, 125.23, 122.24, 120.43, 114.05, 110.07, 65.05, 29.25, 28.85, 26.60, 25.57. MS: calcd. For C_24_H_20_F_6_N_4_O_3_S^+^ [M+H]^+^: 559.1160; HRMS found: m/z 559.1326.

*Synthesis of compound* **13**: Compound **12** (279 mg, 0.5 mmol), Cysteine (66 mg, 0.55 mmol) and DIPEA (129 mg, 1.0 mmol) were dissolved in DCM/MeOH (1:1, 10 mL). The solution was then kept stirring at room temperature for 2 h. MnO_2_ (530 mg, 5.0 mmol) was added into the reaction mixture and the reaction was continued at room temperature overnight. The solvent was removed and purified by flash chromatography on silica gel to give the desired product as a light yellow solid. Yield: 297mg (80%). ^1^H NMR (400 MHz, DMSO-*d*_6_) *δ* 10.06 (s, 1H), 8.97 (d, *J* = 10.6 Hz, 1H), 8.69 (s, 1H), 8.50 (s, 2H), 8.38 (s, 1H), 8.30 (s, 1H), 8.03 (d, *J* = 8.9 Hz, 1H), 7.57 (d, *J* = 10.8 Hz, 1H), 4.13 (t, *J* = 6.5 Hz, 2H), 3.32 (q, *J* = 6.6 Hz, 2H), 1.72 – 1.63 (m, 2H), 1.62 – 1.53 (m, 2H), 1.41 (m, 4H). ^13^C NMR (101 MHz, DMSO-*d*_6_) *δ* 163.50, 162.04, 161.55, 158.69, 154.09, 148.87, 148.69, 138.80, 137.14, 136.52, 131.90, 131.05 (q, *J* = 33 Hz), 128.44, 128.40, 125.17, 124.95, 124.02, 122.23, 119.32, 110.56, 64.89, 29.24, 28.88, 26.61, 25.58. MS: calcd. For C_27_H_22_F_6_N_4_O_5_S_2_^+^ [M+H]^+^: 661.0936; HRMS found: m/z 661.1047.

*Synthesis of compound* **14**: Compound **13** (66 mg, 0.1 mmol), 5,8,11,14-Tetraoxa-2-azahexadecanoic acid,16-amino-,1,1-dimethyl ester (50 mg, 0.15 mmol), HBTU (57 mg, 0.15 mmol) and DIPEA (32 mg, 0.25mmol) were dissolved in 5 mL dry THF. The solution was then kept stirring at room temperature for 3 h. The solvent was removed and purified by flash chromatography on silica gel to give a light yellow solid. The solid was then dissolved in a solution consisting of DCM and TFA (v/v = 70/30, 5mL). The reaction mixture was then stirred at room temperature for 1 h. The solvent was removed under vacuum to give desired product as a light yellow solid. Yield: 65 mg (75%). ^1^H NMR (400 MHz, DMSO-*d*_6_) *δ* 10.08 (s, 1H), 9.01 (t, *J* = 5.3 Hz, 1H), 8.52 (s, 1H), 8.50 (s, 2H), 8.47 (t, *J* = 5.7 Hz, 1H), 8.42 (s, 1H), 8.30 (s, 1H), 8.04 (d, *J* = 8.9 Hz, 1H), 7.56 (dd, *J* = 9.0, 1.6 Hz, 1H), 4.14 (t, *J* = 6.5 Hz, 2H), 3.55 (dq, *J* = 15.6, 10.2, 7.1 Hz, 18H), 3.36 – 3.28 (m, 2H), 2.97 (t, *J* = 8.0 Hz, 2H), 1.72 – 1.63 (m, 2H), 1.62 – 1.52 (m, 2H), 1.41 (s, 4H). ^13^C NMR (101 MHz, DMSO-*d*_6_) *δ* 163.52, 161.34, 160.43, 158.44, 154.12, 151.53, 148.67, 138.82, 137.15, 136.57, 131.06 (q, *J* = 33 Hz), 128.44, 127.55, 125.17, 124.95, 124.05, 122.24, 119.41, 110.46, 70.24, 70.21, 70.11, 70.06, 70.02, 69.24, 67.12, 64.91, 39.12, 39.06, 29.25, 28.89, 26.61, 25.58. MS: calcd. For C_37_H_44_F_6_N_6_O_8_S_2_^+^ [M+H]^+^: 879.2566; HRMS found: m/z 879.2764.

*Synthesis of compound* **15**: Compound **14** (43 mg, 0.05mmol), 1,4,7,10-Tetraazacyclododecane-1,4,7-tris-tert-butyl acetate-10-acetic acid (28 mg, 0.05 mmol), HBTU (19 mg, 0.05 mmol) and DIPEA (20 mg, 0.15mmol) were dissolved in 2 mL dry THF. The solution was then kept stirring at room temperature for another 3 h. The solvent was rapidly removed under vacuum and the residues were dissolved in a solution consisting of DCM and TFA (v/v = 5/95, 5mL). The reaction mixture was then stirred at room temperature for another 3 h. The solvent was removed under vacuum to give compound **15** as light yellow solid, which was directly used for next step without further purification. ^1^H NMR (400 MHz, DMSO-*d*_6_) *δ* 10.08 (s, 1H), 9.03 (t, *J* = 5.4 Hz, 1H), 8.60 (s, 1H), 8.51 (s, 3H), 8.46 (t, *J* = 5.8 Hz, 1H), 8.43 – 8.37 (m, 1H), 8.29 (s, 1H), 8.03 (d, *J* = 8.9 Hz, 1H), 7.56 (dd, *J* = 9.0, 2.0 Hz, 1H), 4.13 (t, *J* = 6.5 Hz, 2H), 4.00 (m, 8H), 3.61 – 3.46 (m, 18H), 3.43 (d, *J* = 5.5 Hz, 2H), 3.38 (s, 4H), 3.33 (dd, *J* = 12.9, 6.8 Hz, 4H), 3.29 – 3.25 (m, 2H), 3.12 (s, 8H), 1.63 (dt, *J* = 33.3, 6.7 Hz, 4H), 1.41 (s, 4H). ^13^C NMR (101 MHz, DMSO-*d*_6_) *δ* 163.55, 161.34, 160.45, 158.75 (q, *J* = 33 Hz, CF_3_COOH), 158.45, 154.13, 151.53, 148.68, 138.82, 137.17, 136.57, 131.07 (q, *J* = 33 Hz), 128.44, 127.52, 125.16, 125.14, 124.95, 124.04, 122.23, 119.43, 118.67(q, *J* = 295 Hz, CF_3_COOH), 110.49, 70.26, 70.22, 70.20, 70.15, 70.02, 69.22, 69.17, 64.92, 55.18, 54.38, 53.06, 51.01, 48.88, 48.53, 39.12, 29.23, 28.88, 26.60, 25.57. MS: calcd. For C_53_H_70_F_6_N_10_O_15_S_2_^+^ [M+H]^+^: 1265.4368; MALDI-MS found: m/z 1265.6271.

*Synthesis of probe* **1-Gd-ctrl**: To a solution of compound **15** (60 mg, 0.05 mmol) in MeOH (2 ml) was carefully added a solution of 1 M NaHCO_3_ to adjust pH value to 7.0. Then, a solution of GdCl_3_ (185 mg, 0.5 mmol) in 1 mL water was added. After stirring at r.t. for 10 min, the pH value of the reaction solution was further adjusted to 7.0 using 1 M NaHCO_3_, and the reaction mixture was stirred at r.t. overnight. After reaction, the reaction mixture was centrifuged (4000 rpm, 3 min), and the supernatant was purified by HPLC to give probe **1-Gd-ctrl** as a light yellow solid. Yield: 39 mg (56%). MS: calcd. For C_53_H_67_F_6_N_10_O_15_S_2_Gd^+^ [M+H]^+^: 1420.3374; HRMS found: m/z 1420.3654.

**Scheme S4.** Synthesis of probe **1-Gal-ctrl**. Reaction conditions: (a) HBTU, Alkyne-PEG_4_-NH_2_, 3 h, 87%; (b) CuSO_4_, VC, 1 h, 54%;

*Synthesis of compound* **16**: Compound **13** (66 mg, 0.1 mmol), Triethylene glycol 2-aminoethyl propargyl ether (35 mg, 0.15 mmol), HBTU (57 mg, 0.15 mmol) and DIPEA (32 mg, 0.25mmol) were dissolved in 5 mL dry THF. The solution was then kept stirring at room temperature for 3 h. The solvent was removed and purified by flash chromatography on silica gel to give a light yellow solid. Yield: 76 mg (87%). ^1^H NMR (400 MHz, DMSO-*d*_6_) *δ* 10.06 (s, 1H), 8.97 (t, *J* = 5.4 Hz, 1H), 8.51 (s, 1H), 8.50 (s, 2H), 8.44 (t, *J* = 5.7 Hz, 1H), 8.40 (s, 1H), 8.30 (s, 1H), 8.03 (d, *J* = 8.9 Hz, 1H), 7.56 (dd, *J* = 9.0, 1.8 Hz, 1H), 4.17 – 4.08 (m, 4H), 3.54 (dt, *J* = 24.0, 8.3 Hz, 16H), 3.40 (t, *J* = 2.3 Hz, 1H), 3.33 (q, *J* = 6.6 Hz, 2H), 1.74 – 1.63 (m, 2H), 1.63 – 1.51 (m, 2H), 1.41 (s, 4H). ^13^C NMR (101 MHz, DMSO-*d*_6_) *δ* 163.50, 161.30, 160.38, 158.45, 154.10, 151.55, 148.67, 138.79, 137.14, 136.58, 131.05 (q, *J* = 33 Hz), 128.44, 128.40, 127.50, 125.16, 124.95, 124.02, 122.24, 119.36, 110.46, 80.74, 77.50, 70.25, 70.23, 70.04, 69.94, 69.25, 68.94, 64.90, 57.92, 39.15, 29.25, 28.89, 26.62, 25.59. MS: calcd. For C_38_H_41_F_6_N_5_O_8_S_2_^+^[M+H]^+^: 874.2301; HRMS found: m/z 874.2541.

*Synthesis of probe* **1-Gal-ctrl**: Compound **8** (22 mg, 0.04 mmol) and CH_3_ONa (27 mg, 0.5 mmol) were dissolved in 1 mL CH_3_OH. The solution was then kept stirring at room temperature for 6 h. Dowex® 50WX8-400 ion-exchange resin was slowly added into the reaction mixture to adjust the pH to ~5-6. The mixture was filtered and the filtrate was collected. The solvent was removed and the residues were dissolved in 0.5 mL tert-Butanol. Compound **16** (17.4 mg, 0.02 mmol) was dissolved in the solution. CuSO_4_ (1.5 mg, 0.01 mmol) and Sodium L-Ascorbate (3.96 mg, 0.02 mmol) was dissolved in 0.2 mL H_2_O and added to the reaction mixture. The solution was kept stirring at r.t. for 30 min. After the reaction, the mixture was purified by HPLC to afford probe **1-Gal-ctrl** after lyophilization. Yield: 13.5 mg (54%). ^1^H NMR (400 MHz, DMSO-*d*_6_) *δ* 10.05 (s, 1H), 8.97 (t, *J* = 5.4 Hz, 1H), 8.51 (s, 1H), 8.50 (s, 2H), 8.44 (t, *J* = 5.8 Hz, 1H), 8.41 – 8.37 (m, 1H), 8.30 (s, 1H), 8.09 – 7.99 (m, 2H), 7.56 (dd, *J* = 9.0, 2.1 Hz, 1H), 4.50 (d, *J* = 3.6 Hz, 4H), 4.14 (t, *J* = 6.6 Hz, 2H), 4.09 (d, *J* = 7.0 Hz, 1H), 3.81 (t, *J* = 5.3 Hz, 2H), 3.64 – 3.43 (m, 30H), 3.39 – 3.22 (m, 6H), 1.68 (p, *J* = 6.7 Hz, 2H), 1.59 (p, *J* = 7.0 Hz, 2H), 1.41 (s, 4H). ^13^C NMR (101 MHz, DMSO-*d*_6_) *δ* 168.28, 166.06, 165.16, 163.23, 158.86, 156.31, 153.44, 149.01, 143.54, 141.93, 141.33, 135.82 (q, *J* = 33 Hz), 133.19, 129.89, 129.70, 129.44, 128.79, 126.99, 124.14, 115.28, 108.80, 80.42, 78.69, 75.69, 75.01, 74.97, 74.89, 74.82, 74.78, 74.73, 74.15, 73.99, 73.91, 73.36, 72.91, 69.67, 68.72, 65.65, 54.51, 43.90, 34.00, 33.64, 31.36, 30.33. MS: calcd. For C_52_H_68_F_6_N_8_O_17_S_2_^+^ [M+Na]^+^: 1277.3964; MALDI-MS found: m/z 1277.6602.


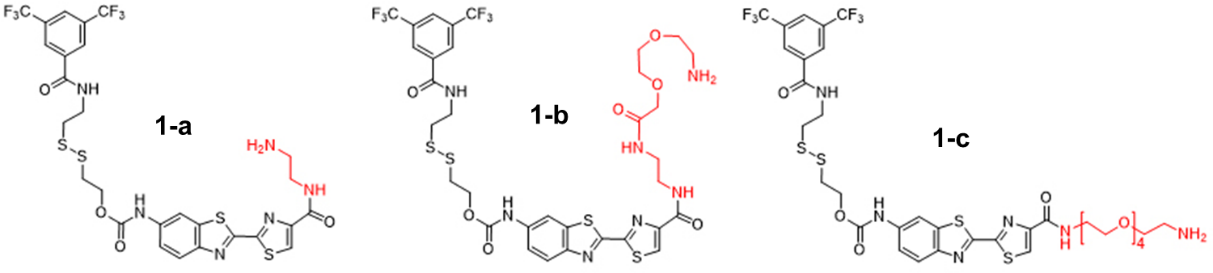


**Figure S1.** Chemical structures of compounds **1-a**, **1-b**, and **1-c**.


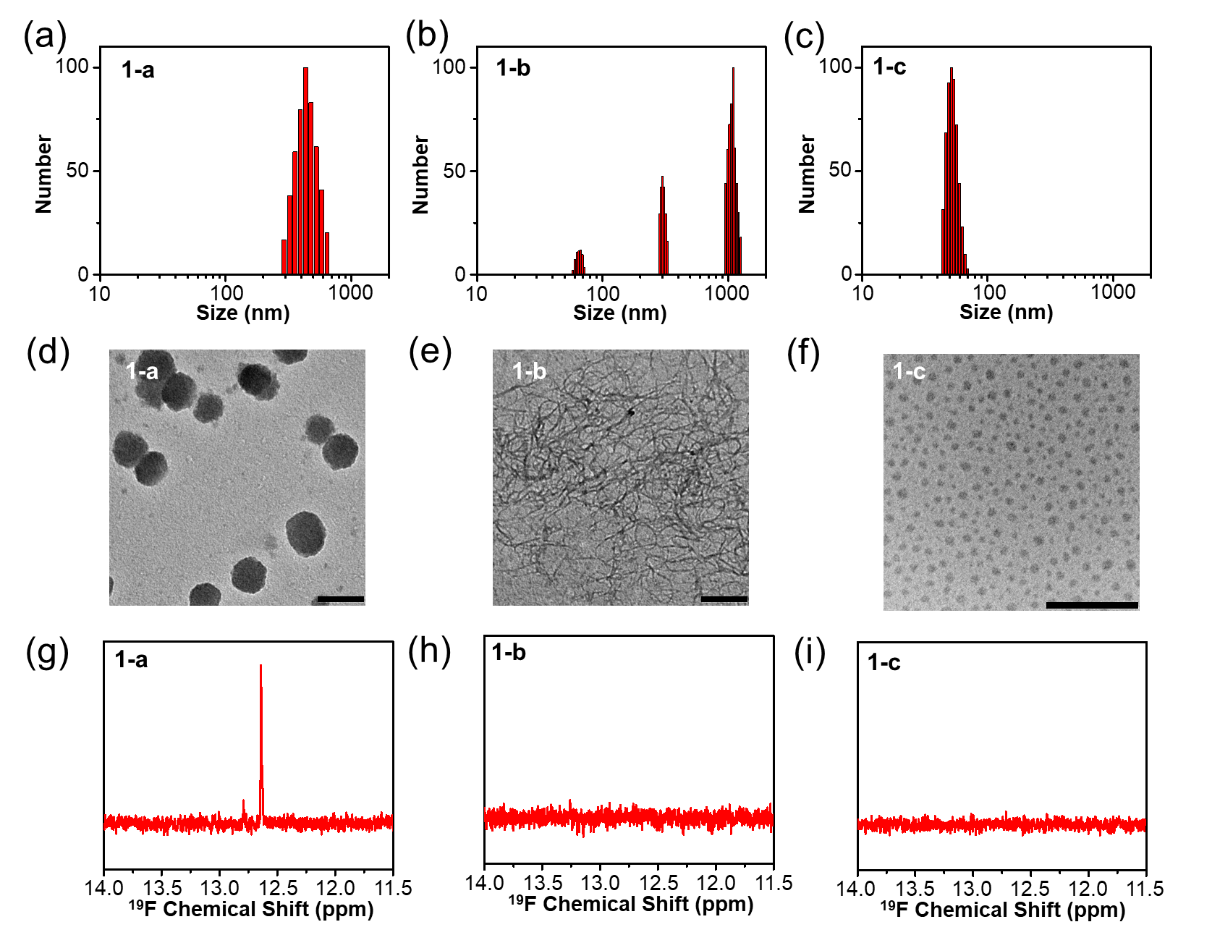


**Figure S2.** DLS results of (a) **1-a**, (b) **1-b**, and (c) **1-c** in PBS buffer (200 μM). TEM images of (d) **1-a**, (e) **1-b**, and (f) **1-c** (200 μM). Scale bar: 500 nm. ^19^F NMR spectra of (g) **1-a**, (h) **1-b**, and (i) **1-c** (200 μM). The results show that compound **1a** with a short ethylenediamine end self-assembles into large aggregates, and the ^19^F MRS signal is not completely quenched; compound **1-b** self-assembles into nanofibers, which can precipitate out in a few hours; compound 1-c with a hydrophilic PEG_4_-NH_2_ end self-assembles into monodisperse nanoparticles, where the ^19^F MRS signal is completely quenched.


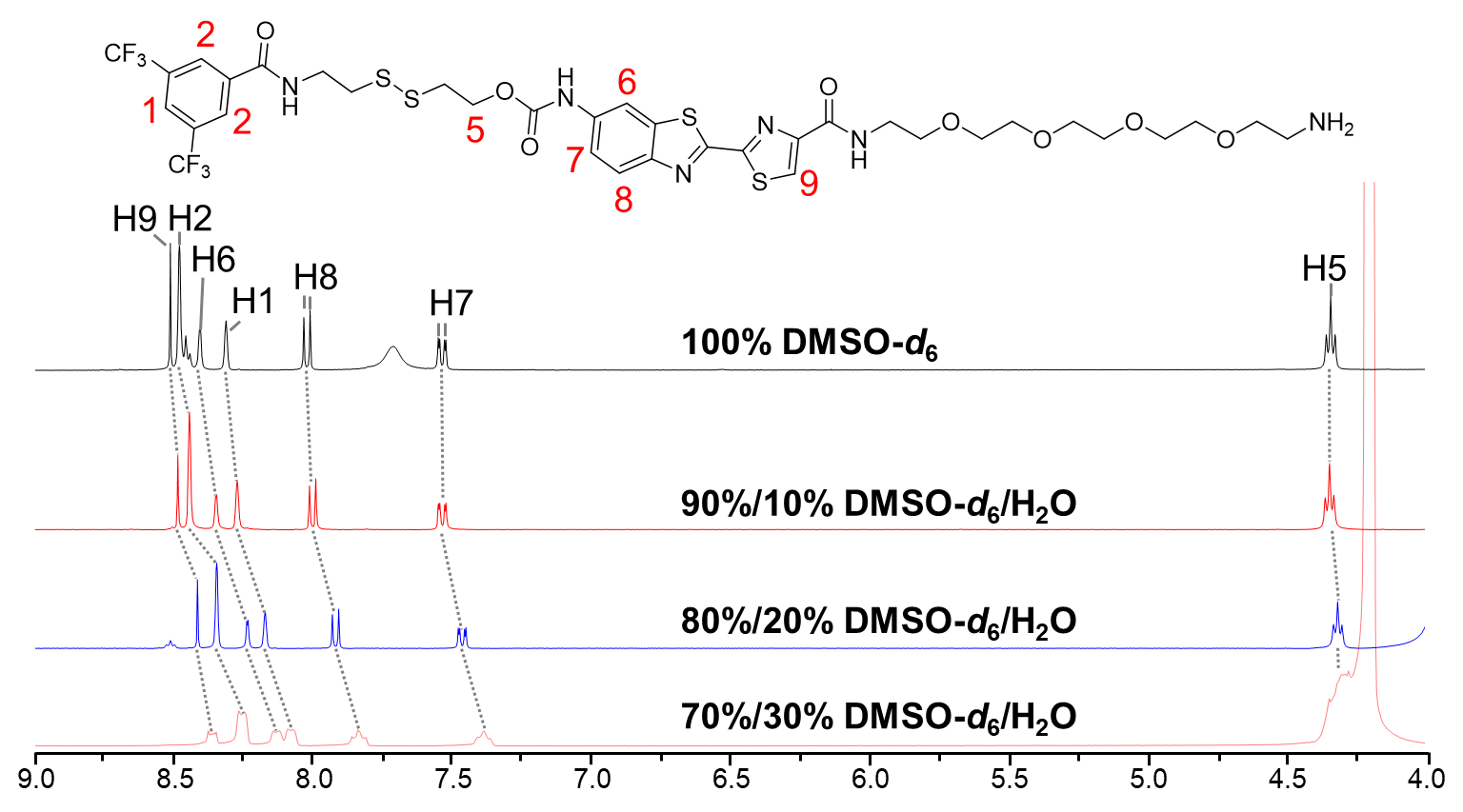


**Figure S3.** ^1^H NMR spectra of **1-c** (3 mM)in in DMSO-*d*_6_ (100%), DMSO-*d*_6_/D_2_O (90%/10%), DMSO-*d*_6_/D_2_O (80%/20%) and DMSO-*d*_6_/D_2_O (70%/30%). **1-c** is present as free molecule when dissovled in 100% DMSO-d_6_, and can gradually assemble into nanoaggregates as the amount of D_2_O increases. The ^1^H NMR spectra show that the chemical shifts of the aromatic hydrogens in both the fluorinated motifs (H1 and H2) and oxyluciferin moieties (H6-H9) become broader and shift to high field as the D_2_O contents increase, suggesting the π-π stacking effects occurred between both the 3,5-bis(trifluoromethyl)benzene ring and amino oxyluciferin scaffold within self-assembled nanoparticles from **1-c**.


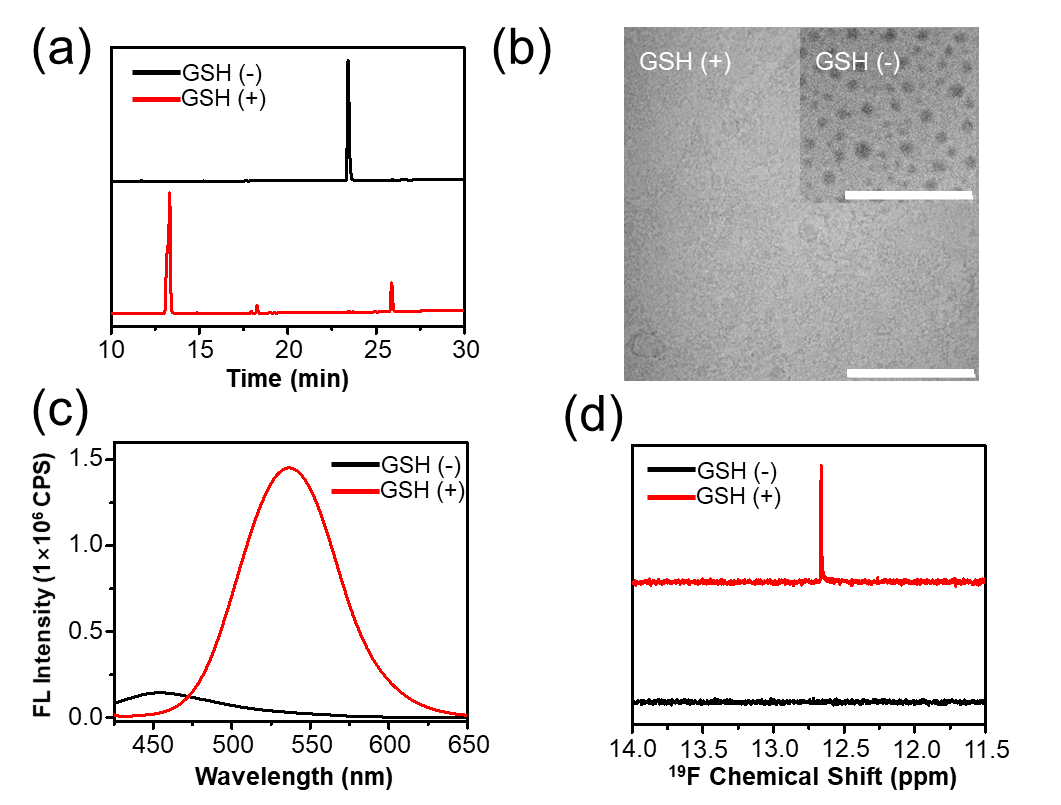


**Figure S4.** GSH-driven disulfide reduction and disassembly of **1-c**-based nanoparticles. (a) HPLC of **1-c**-based nanoparticles (200 μM) with and without incubation with GSH (10 mM, PBS, pH 7.4) at 37 °C for 60 min. (b) TEM of **1-c**-based nanoparticles (200 μM) with and without (inset) incubation with GSH (10 mM, PBS, pH 7.4). Scale bar: 500 nm. (c) Fluorescence spectra of **1-c**-based nanoparticles (200 μM) with and without incubation with GSH (10 mM, PBS, pH 7.4). The fluorescence was measured after 1 to 10 dilution with the PBS buffer. (d) ^19^F NMR spectra of **1-c**-based nanoparticles (200 μM) with and without incubation with GSH (10 mM, PBS, pH 7.4). These results show that **1-c**-based nanoparticles can be reduced by GSH and undergo disassembly into cleaved products, switching on fluorescence and ^19^F-MRS signals.


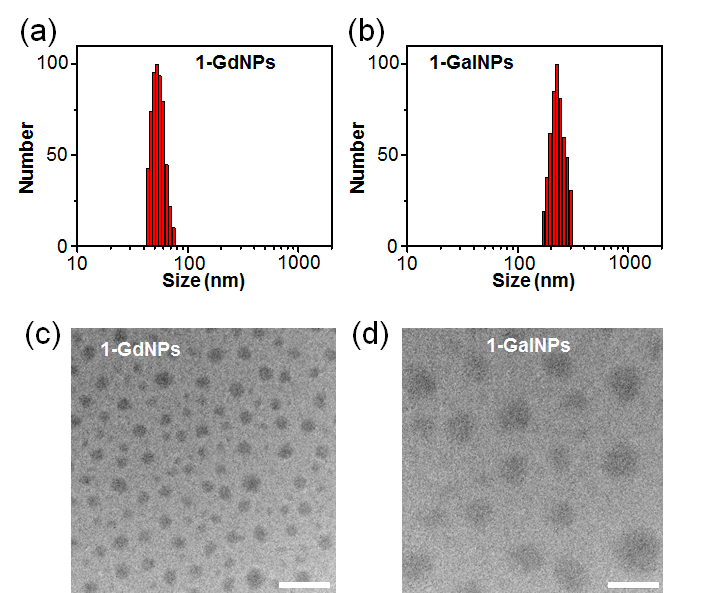


**Figure S5.** DLS analysis of **1-GdNPs** (a) and**1-GalNPs** (b) (200 μM) in PBS buffer. TEM images of **1-GdNPs** (c) and **1-GalNPs** (d). Scale bar: 200 nm.


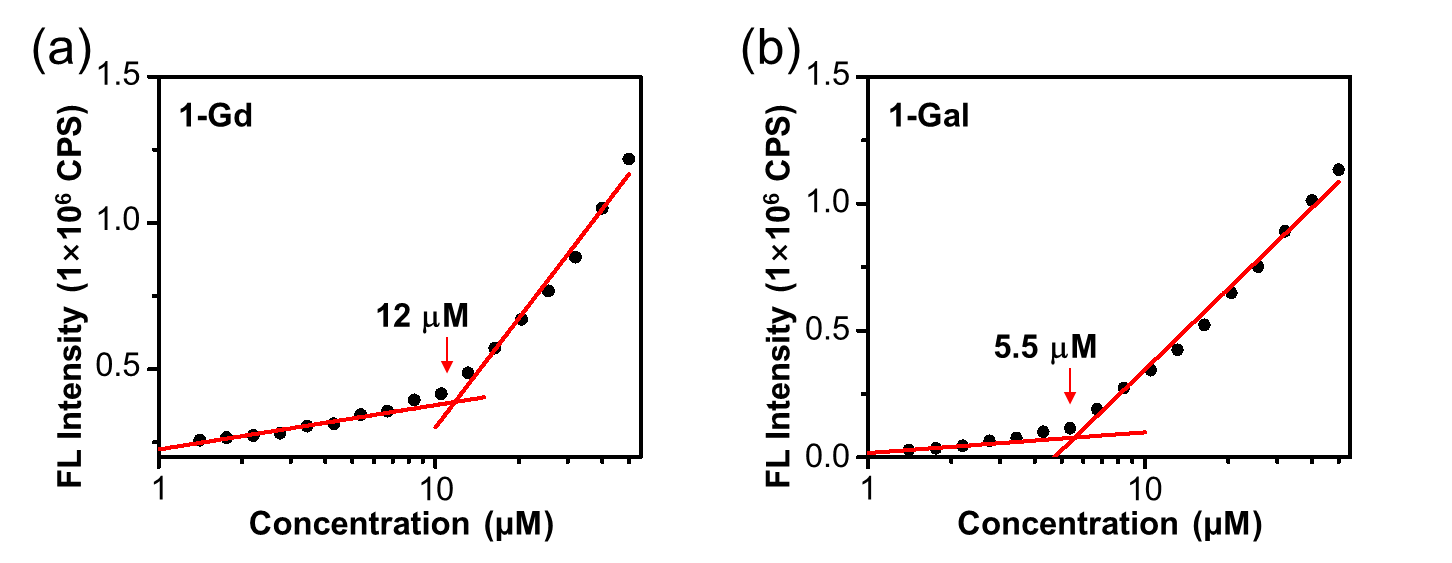


**Figure S6.** (a) Plots of fluorescence intensity versus concentration of **1-Gd** or **1-Gal** (b) in PBS buffer determine the CMC of **1-Gd**. The CMC value of **1-Gd** and **1-Gal** was obtained from the intersection of the two linear fit lines, which was found to be ~12 μM and ~5.5 μM, respectively.


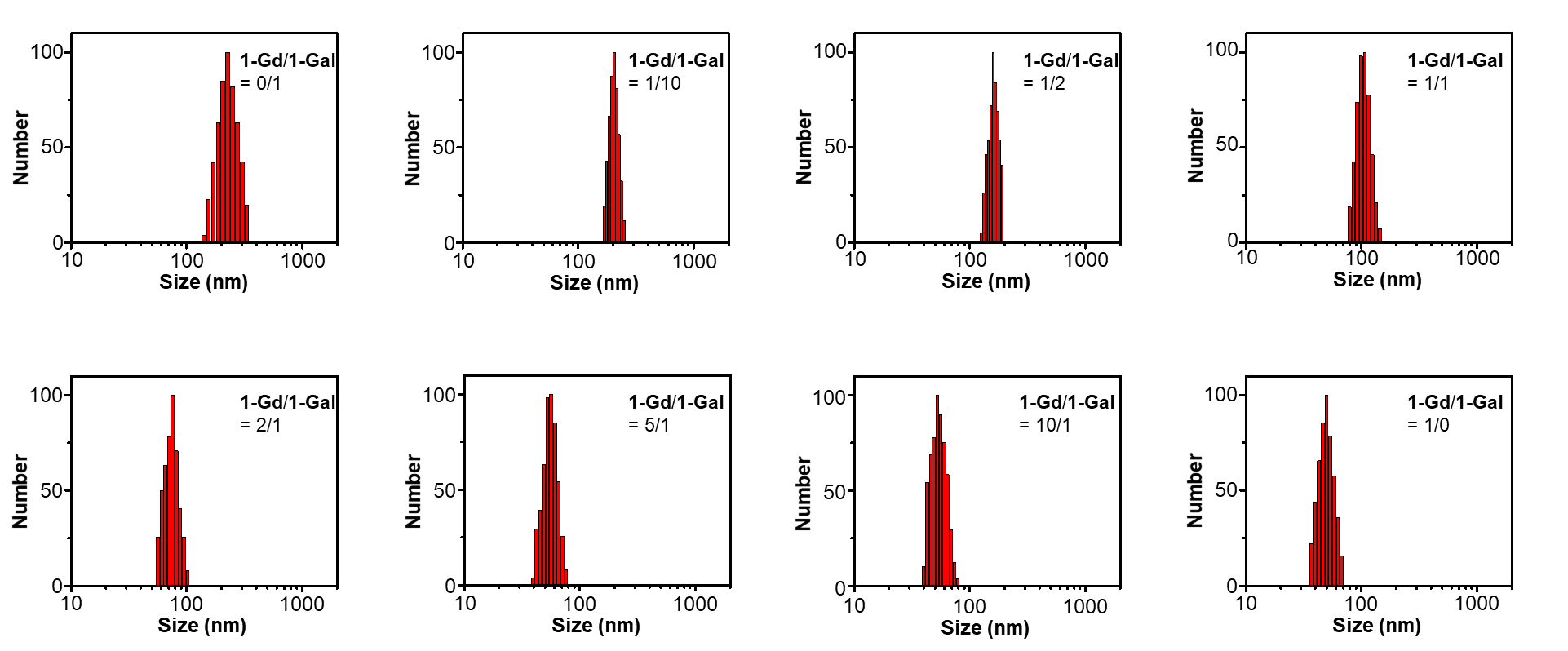


**Figure S7.** DLS analysis of nanoparticles co-assembly from probes **1-Gd** and **1-Gal** at different mole ratios. The results show that probes **1-Gd** and **1-Gal** at different mole ratios can well assemble into monodispersed nanoparticles. The hydrodynamic size of NPs decreased as the ratio of **1-Gd**/**1-Gal** increased, and when the **1-Gd**/**1-Gal** ratio increases to 5 or more, the hydrodynamic size was stabilized to be as small as that of **1-GdNPs**.


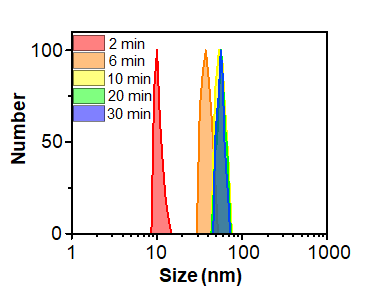


**Figure S8.** DLS size profiles show the time-dependent formation of nanoparticles via co-assembly of probes **1-Gal** and **1-Gd** (1/5, 200 μM) in a PBS buffer (pH 7.4).


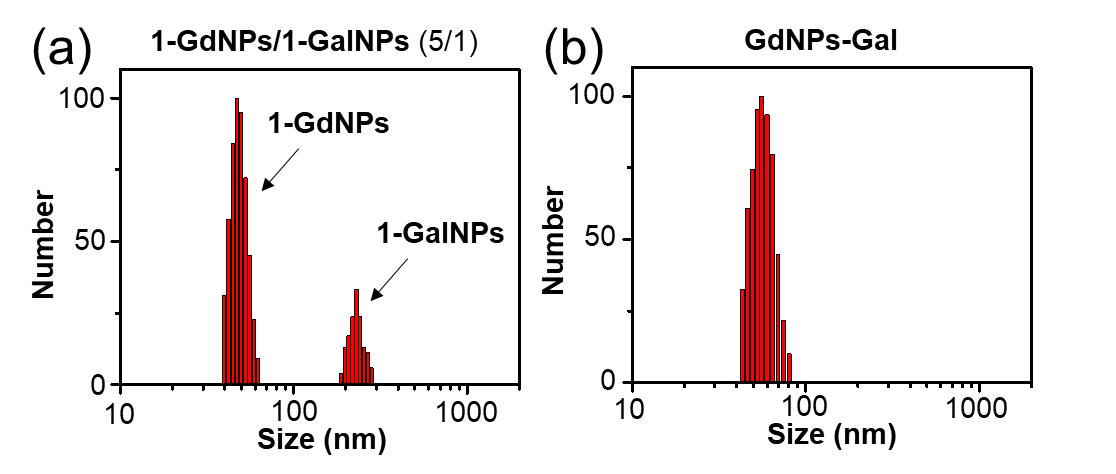


**Figure S9.** (a) DLS analysis of a mixture consisting of pre-formed **1-GdNPs** and **1-GalNPs** at a ratio of 5 in a PBS buffer show two distinct sizes corresponding to **1-GdNPs** and **1-GalNPs**. (b) DLS analysis of **GdNPs-Gal** formed by co-assembly of **1-Gd** and **1-Gal** at a mole ratio of 5/1 in PBS buffer show a monodisperse size. These results indicate that **GdNPs-Gal** was formed via efficient co-assembly of probes **1-Gd** and **1-Gal**.


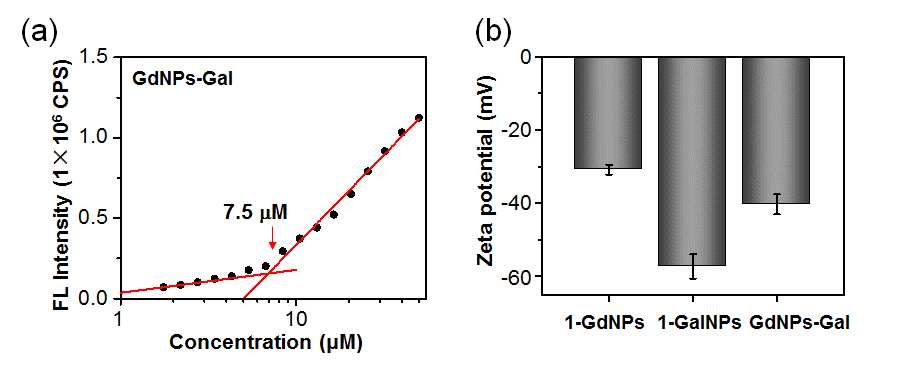


**Figure S10.** (a) Plots of the fluorescence intensity versus concentrations of **GdNPs-Gal** in PBS buffer determine the CMC of **GdNPs-Gal**, which was found to be ~7.5 μM according to the intersection of the two linear fit lines. (b) Zeta potential of probe **1-GdNPs**, **1-GalNPs** and **GdNPs-Gal**. Data denote mean ± SD (n = 3).


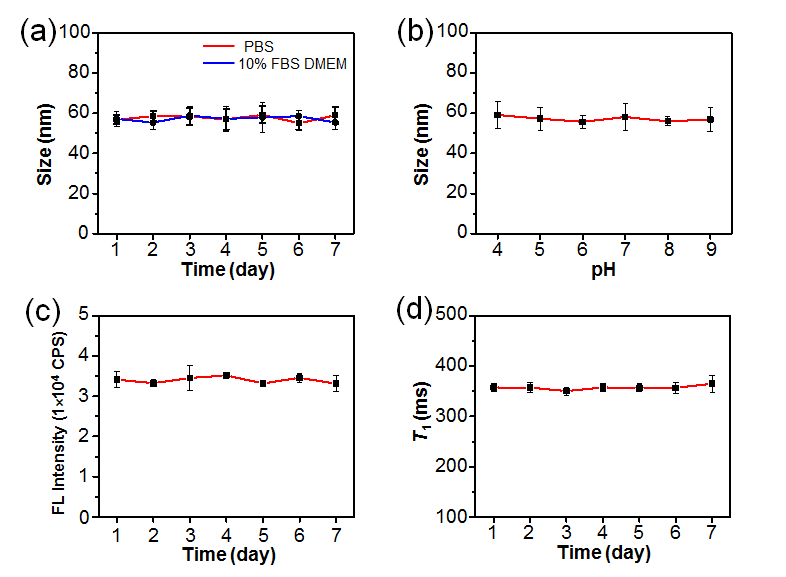


**Figure S11.** Evaluation of the stability of **GdNPs-Gal**. (a) Comparison of the mean hydrodynamic size of **GdNPs-Gal** (200 μM) following incubation in PBS buffer (pH 7.4) or DMEM containing 10% FBS for 7 days. (b) Comparison of the mean hydrodynamic size of **GdNPs-Gal** (200 μM) following incubation in aqueous buffer under pH range of 4.0-9.0. (c) Comparison of the fluorescence intensity of **GdNPs-Gal** (200 μM) following incubation in PBS buffer (pH 7.4) for 7 days. λ_ex/em_ =405/450 nm. (d) Comparison of the *T*_1_ values of **GdNPs-Gal** (200 μM) following incubation in PBS buffer (pH 7.4) for 7 days. These results suggest that **GdNPs-Gal** is stable under physiological conditions. Data denote mean ± SD (n = 3).


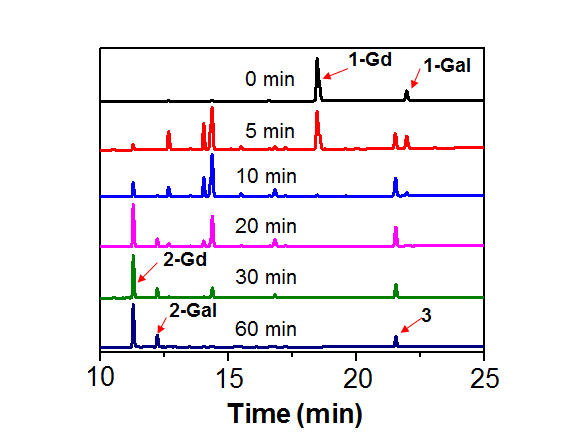


**Figure S12.** HPLC analysis of **GdNPs-Gal** (200 μM) incubated with GSH (10 mM) in PBS buffer at 37 °C for indicated time. The results showed that probe **1-Gd** and **1-Gal** within **GdNPs-Gal** could be rapidly reduced by GSH and ultimately converted into the cleaved products **2-Gd**, **2-Gal** and **3** after 60 min.


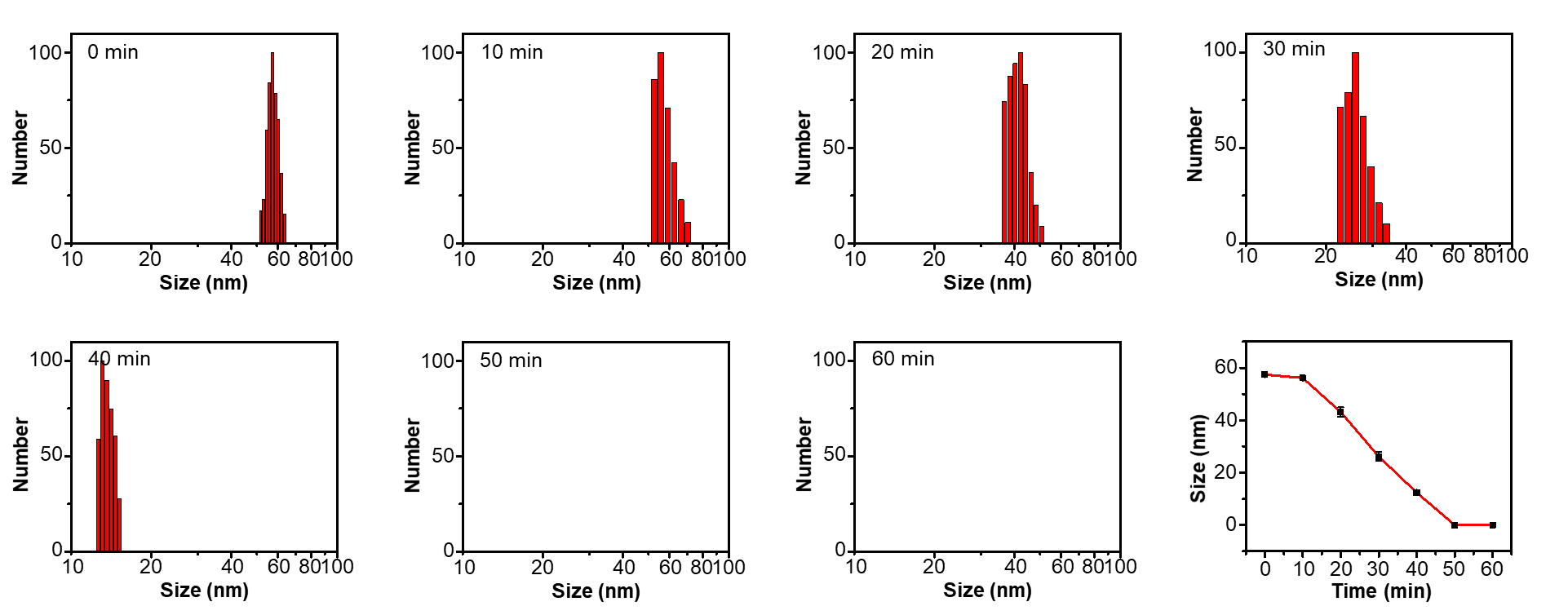


**Figure S13.** DLS analysis of **GdNPs-Gal** (200 μM) following incubation with GSH (10 mM) at 37 °C for the indicated time. The results show that **GdNPs-Gal** can be reduced by GSH and the size is gradually reduced in the solution as a result of disassembly. Data denote mean ± SD (n = 3).


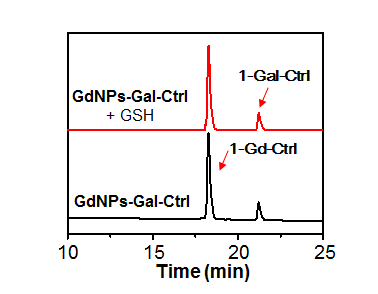


**Figure S14.** HPLC analysis of **GdNPs-Ctrl** (200 μM) with and without incubation with GSH (10 mM, PBS, pH 7.4) at 37 °C for 60 min. **GdNPs-Ctrl** is formed by co-assembly of **1-Gd-ctrl** and **1-Gal-ctrl** (mole ratio **=** 5/1). The results show that **1-Gd-ctrl** and **1-Gal-ctrl** within **GdNPs-Ctrl** cannot be reduced by GSH, due to lack of a disulfide bond.


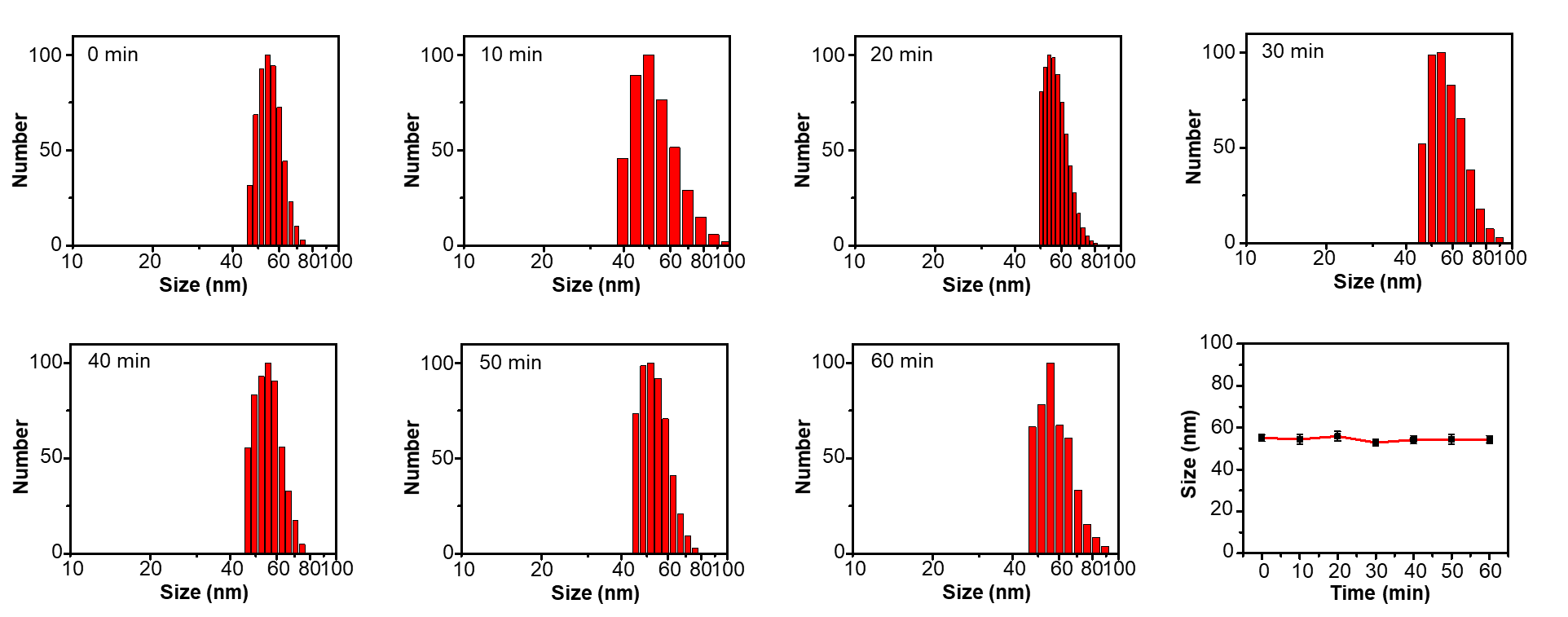


**Figure S15.** DLS results of **GdNPs-Gal-Ctrl** (200 μM) incubated with GSH (10 mM) at 37 °C for the indicated time. The results show that the size of **GdNPs-Gal-Ctrl** was not obviously changed after incubation with GSH, which is in contrast to that of **GdNPs-Gal**, indicating that **GdNPs-Gal-Ctrl** cannot proceed disassembly toward GSH. Data denote mean ± SD (n = 3).


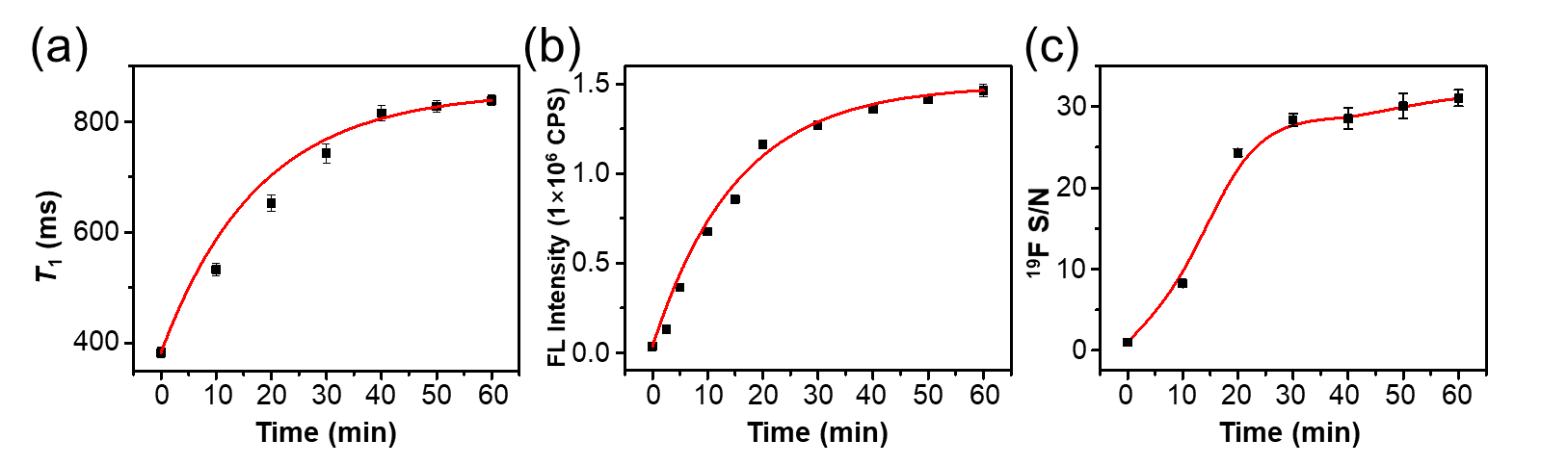


**Figure S16.** (a) Plots of the *T*_1_ values, (b) fluorescence intensity at 535 nm, and (c) S/N of ^19^F MRS signals of **GdNPs-Gal** (200 μM) following incubation with GSH (10 mM) for 0-60 min. The representative fluorescence and ^19^F NMR spectra were shown in Figures 3e and 3f. Data denote mean ± SD (n = 3).


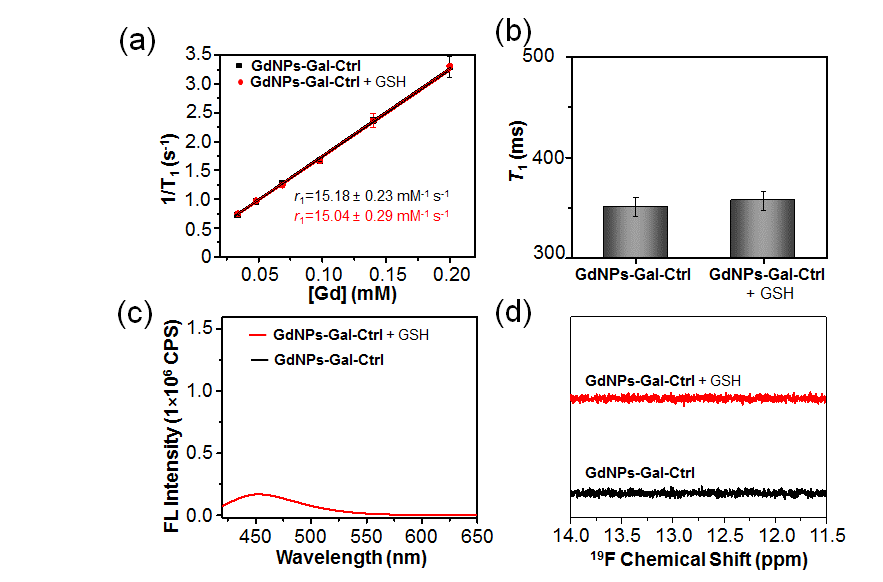


**Figure S17.** (a) Plots of 1/ *T*_1_ versus Gd concentration determine the *r*_1_ relaxivities of **GdNPs-Gal-Ctrl** before (black) and after (red) incubation with GSH (10 mM) for 60 min. (b) *T*_1_ values of **GdNPs-Gal-Ctrl** with and without incubation with GSH at 37 °C for 60 min. (c) Fluorescence spectra of **GdNPs-Gal-Ctrl** before and after incubation with GSH at 37 °C in PBS buffer for 60 min. (d) ^19^F NMR spectra of **GdNPs-Gal-Ctrl** with and without incubation with GSH at 37 °C for 60 min. These results show that the assembled **GdNPs-Gal-Ctrl** cannot react with GSH and stay stable in the solution. Data denote mean ± SD (n = 3).


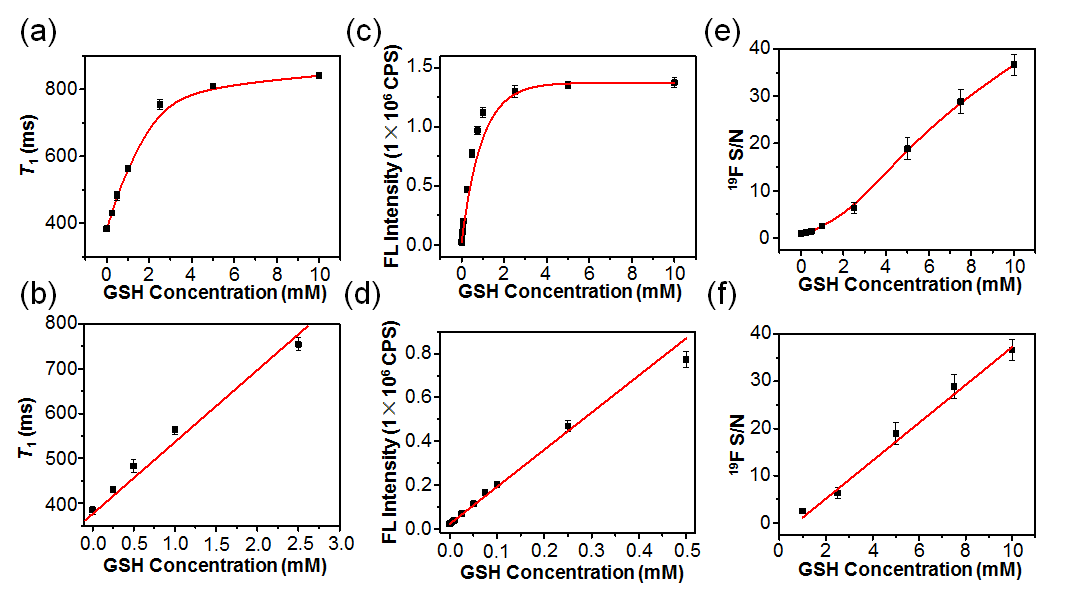


**Figure S18.** (a) Plots of *T*_1_ values, (c) FL intensity at 535 nm, and (e) S/N of ^19^F MRS signals of **GdNPs-Gal** (200 μM) following incubation with 0-10 mM GSH in PBS buffer for 60 min. (b) Linear proportion of the *T*_1_ values versus the concentration of GSH from 0.25 to 2.5 mM, with a detection limit of 0.18 mM (signal-to-noise, S/N = 3). (d) Linear proportion of the FL intensity at 535 nm versus the concentration of GSH from 0 to 500 μM, with a detection limit of 0.70 μM (signal-to-noise, S/N = 3). (f) Plots of the S/N values of ^19^F MRS signals versus the concentration of GSH from 1-10 mM, with a detection limit of 0.25 mM (signal-to-noise, S/N = 3). Data denote mean ± SD (n = 3).


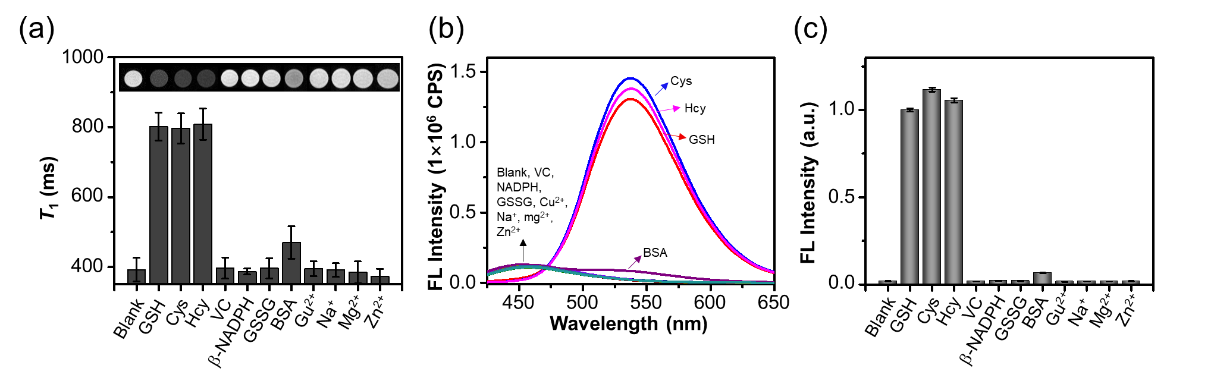


**Figure S19.** Selectivity of **GdNPs-Gal**. (a) *T*_1_ values and (Inset) *T*_1_ weight MR images of **GdNPs-Gal** (200 μM) following incubation with different analyses (10 mM) in PBS buffer for 1 h. (b) Fluorescence spectra and (c) intensity (λ_ex/em_ = 405/535 nm) of **GdNPs-Gal** (200 μM) following incubation with various analyses (10 mM) in PBS buffer for 1 h. The result show that **GdNPs-Gal** can react with GSH, Cys, and Hcy, while no apparent changes in fluorescence and *T*_1_ value were found for BSA, other reductive compounds and biologically relevant metal ions. Data denote mean ± SD (n = 3).


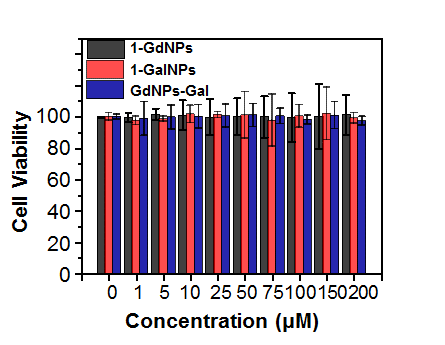


**Figure S20.** The cell viability test. HepG2 cells were incubated with **1-GdNPs**, **1-GalNPs** or **GdNPs-Gal** at 0, 1, 5, 10, 25, 50, 75, 100, 150, and 200 μM for 24 h, and the cell viability was determined by MTT assay. The results show that **1-Gd**, **1-Gal** and **GdNPs-Gal** had little effect on cell viability, suggesting high biocompatibility for cell studies. Data denote mean ± SD (n = 3).


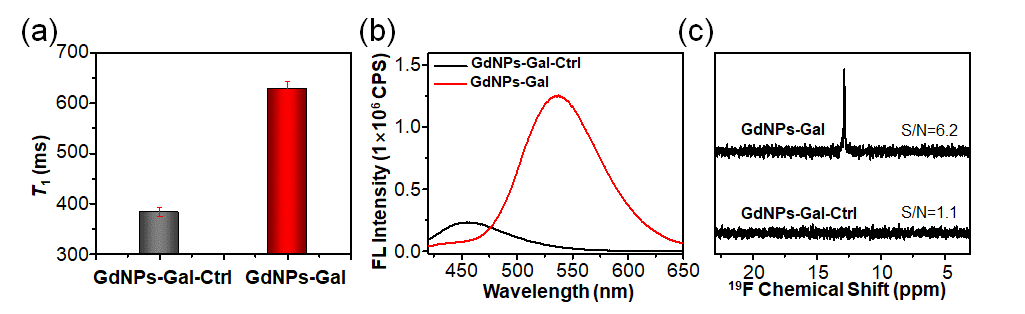


**Figure S21.** Detection of endogenous GSH in cell lysates. (a) *T*_1_ values, (b) fluorescence spectra and (c) ^19^F NMR spectra of HepG2 cell lysates upon incubation with **GdNPs-Gal** or **GdNPs-Gal-Ctrl** (200 μM) at 37 °C for 4 h. These results show that distinct changes in *T*_1_ value, FL intensity and ^19^F MRS signal occur in the lysates after incubation with **GdNPs-Gal**, not **GdNPs-Gal-Ctrl**, suggesting that **GdNPs-Gal** is able to detect endogenous GSH in complex biological environment. Data denote mean ± SD (n = 3).


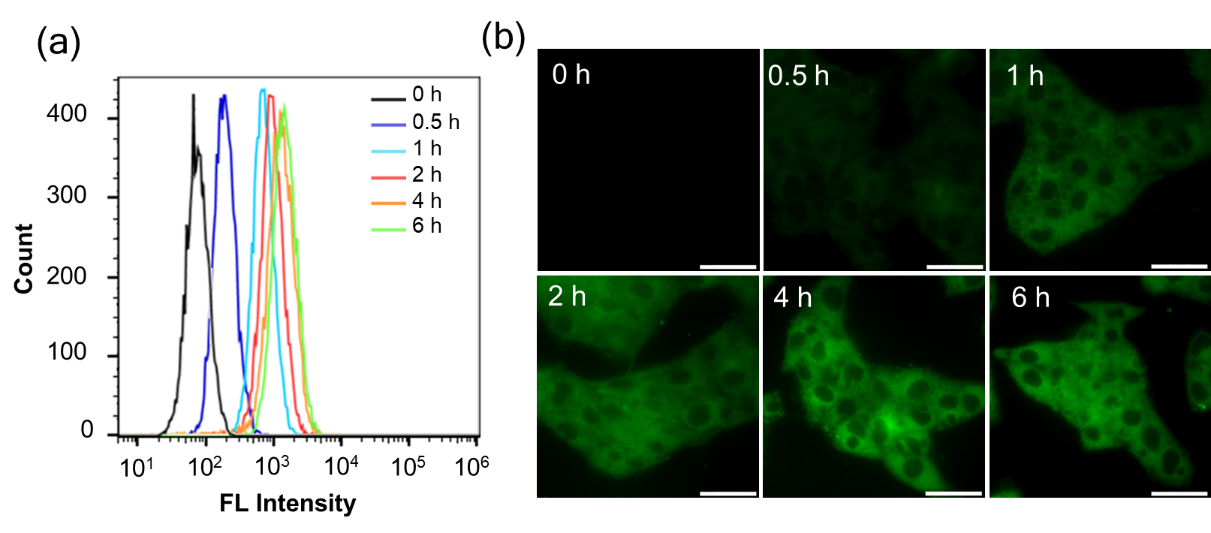


**Figure S22.** (a) Flow cytometric assay of HepG2 cells incubated with **GdNPs-Gal** (50 μM) at 37 °C for different time (0, 0.5, 1, 2, 4 and 6 h). (b) Fluorescence imaging of HepG2 cells incubated with **GdNPs-Gal** (50 μM) for 0-6 h. Scale bar: 20 μm.


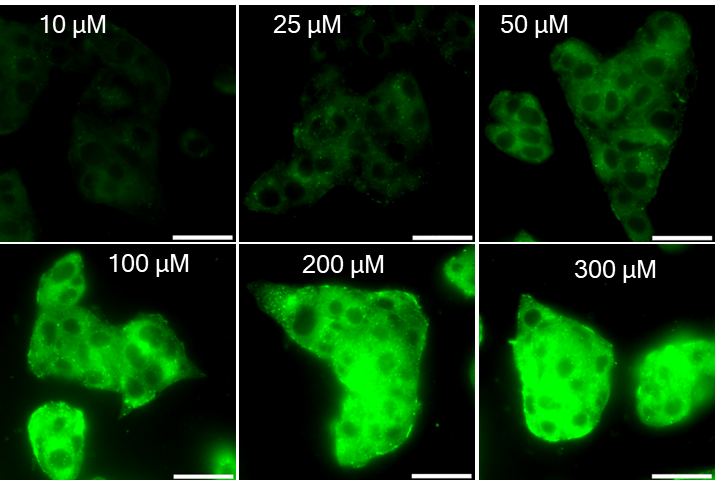


**Figure S23.** Fluorescence images of HepG2 cells incubated with different concentrations of **GdNPs-Gal** (10, 25, 50, 100, 200, and 300 μM) at 37 °C for 4 h. Scale bars: 20 μm.


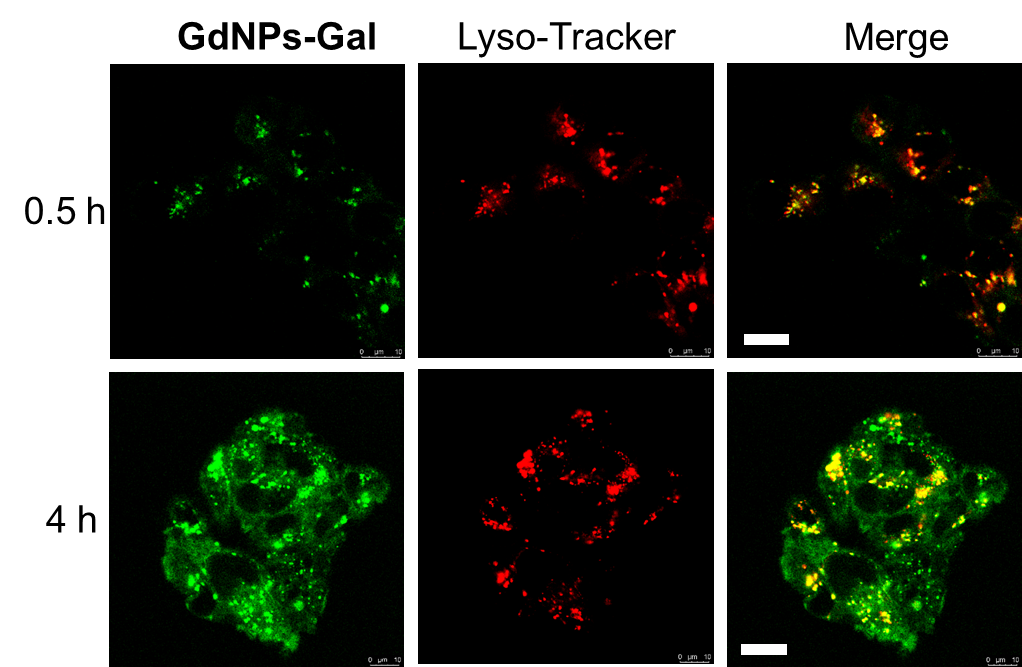


**Figure S24.** Co-localization study of HepG2 cells incubated with the **GdNPs-Gal** (200 μM, green) for 0.5 h and 4 h, following incubation with Lyso-tracker (red) for another 20 min. Scale bars: 10 μm. The result show that **GdNPs-Gal** is mainly distributed in the lysosomes at 0.5 h; after 4 h, the activated **GdNPs-Gal** can escape from the lysosomes and enter cytosol.


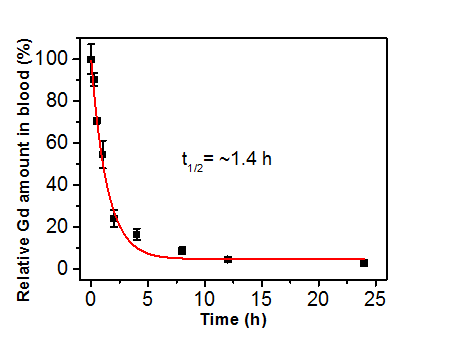


**Figure S25.** Blood circulation curve of **GdNPs-Gal** (0.025 mmol kg^-1^ Gd) after i.v. injection into healthy mice. Values denote mean ± SD (n = 3).


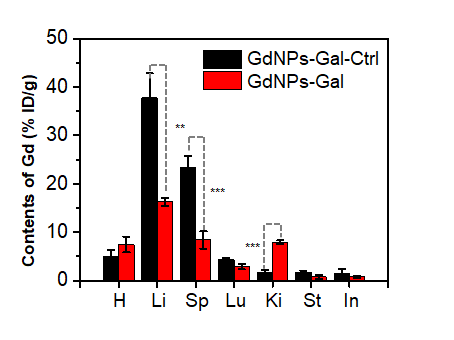


**Figure S26.** Biodistribution evaluation of **GdNPs-Gal** (red) or **GdNPs-Gal-Ctrl** (black) in mice. Healthy nude mice were i.v. injected with **GdNPs-Gal** or **GdNPs-Gal-Ctrl** (0.1 mmol kg^−1^). After 4 h, the mice were sacrificed, and the main organs (e.g., H: heart, Li: liver including gallbladder, Lu: lung, Sp: spleen, Ki: kidneys, St: stomach, In: intestines) were resected and weighted. The amount of Gd^3+^ (% ID/g) in the main organs were determined by ICP-MS. Values denote mean ± SD (n = 3). ** p < 0.01, *** p < 0.001.


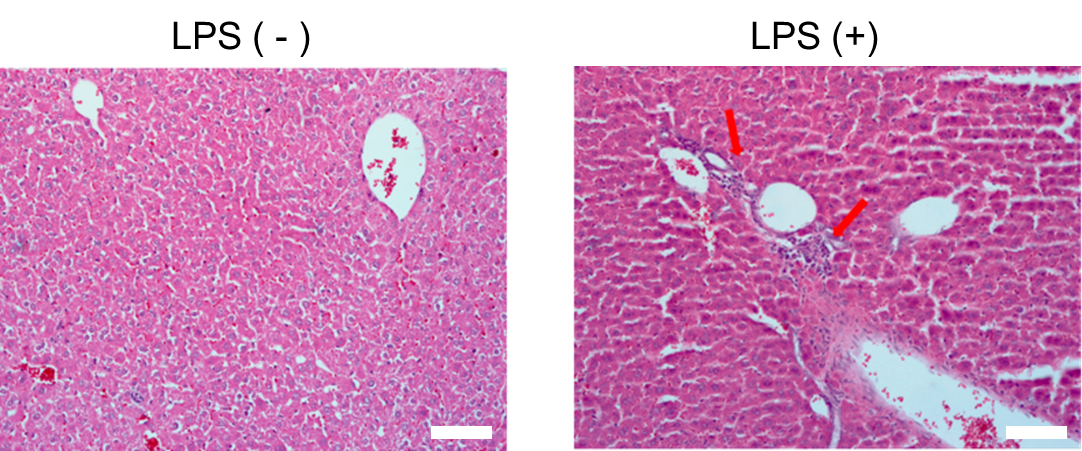


**Figure S27.** Histological examination of liver tissues of mice with and without treatment with LPS. Inflammatory mice were sacrificed 6 h after i.p. injection with LPS (20 mg kg^-1^). Non-treated healthy mice were used as control. Red arrows show the locations of inflammatory cell infiltration. The results validate that i.p. injection of LPS can cause liver inflammation. Scale bars: 50 μm.


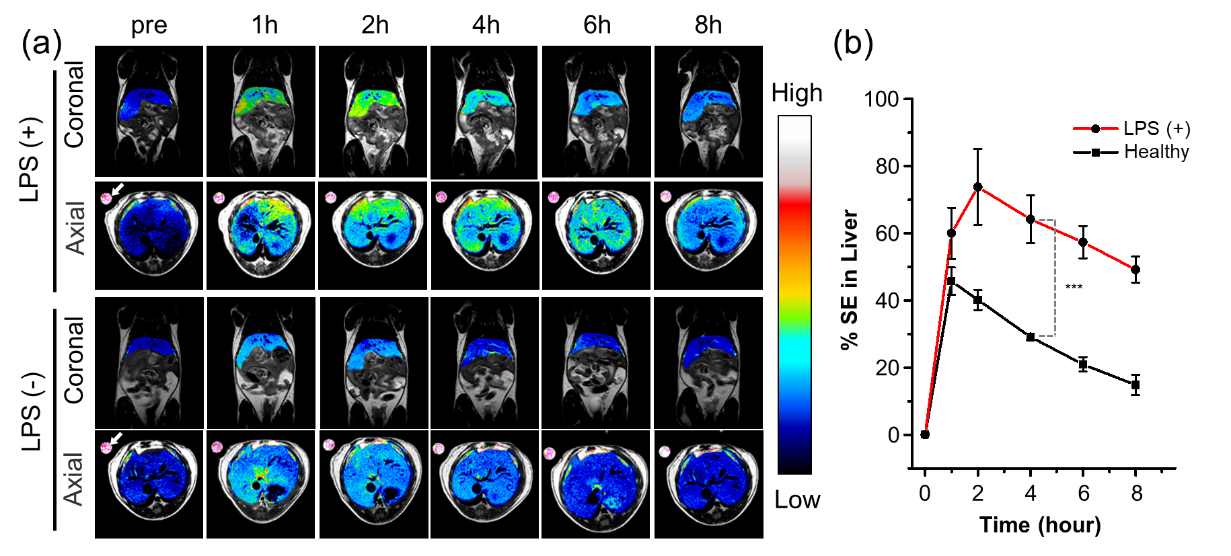


**Figure S28.** In vivo MR imaging of hepatitis. (a) Coronal and Axial *T*_1_ -weight MR images of livers in mice with (up) and without (down) LPS treatment (20 mg kg^-1^). Mice were i.v. injected 0.1 mmol kg^-1^ of **GdNPs-Gal** 6 h after i.p. injection of LPS, and the images were taken before, 1, 2, 4, 6, and 8 h after injection of **GdNPs-Gal**. The MR contrast in livers is shown in pseudo rainbow color for comparison; white arrows in Axial images indicate the Dotarem (1 mM) solution as the internal standard. (b) The average longitudinal % signal enhancement (% SE) in livers after i.v. injection of **GdNPs-Gal** into mice. The results show that the contrast in LPS-treated inflammatory mice is significantly higher than that in healthy mice, suggesting that **GdNPs-Gal** can distinguish hepatitis from healthy livers in living mice. Data denote mean ± SD (n = 3, ***P < 0.001).


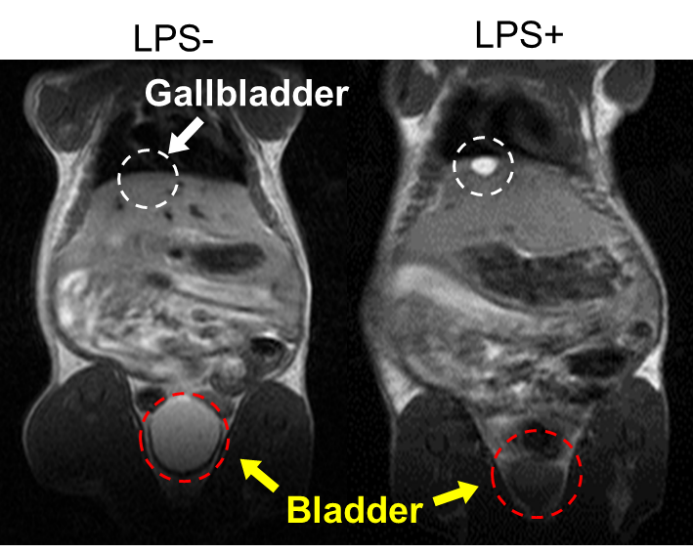


**Figure S29.** Coronal *T*_1_-weighted MR images showed different elimination pathway of **GdNPs-Gal** between healthy mice and hepatitis mice. The results show that **GdNPs-Gal** can be eliminated via renal systems of healthy mice due to the high level of GSH in the liver, which can trigger disassembly of **GdNPs-Gal** into small molecules; in contrast, **GdNPs-Gal** is mainly excreted out via hepatobiliary systems in mice under livre inflammation, owing to the decreased GSH concentration that reduces the ability to trigger disassembly of **GdNPs-Gal** into small molecules.


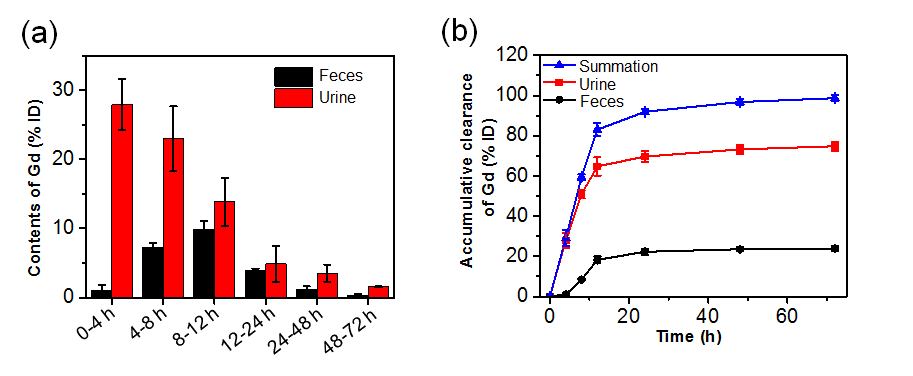


**Figure 30.** ICP-MS analysis shows the clearance of **GdNPs-Gal** in healthy mice. (a) Contents of Gd (% ID) in feces and urine of mice in 0-4, 4-8, 8-12, 12-24, 24-48 and 48-72 h following i.v. injection of **GdNPs-Gal** (0.1 mmol kg^-1^). (b) Plots of the accumulative clearance of Gd (% ID) in urine (red), feces (black) and urine together with feces (summation, blue) following i.v. injection of **GdNPs-Gal**. Values denotes mean ± SD (n = 3).

**Figure S31.** ^1^H-NMR spectra of compound **2** (DMSO-*d*_6_)


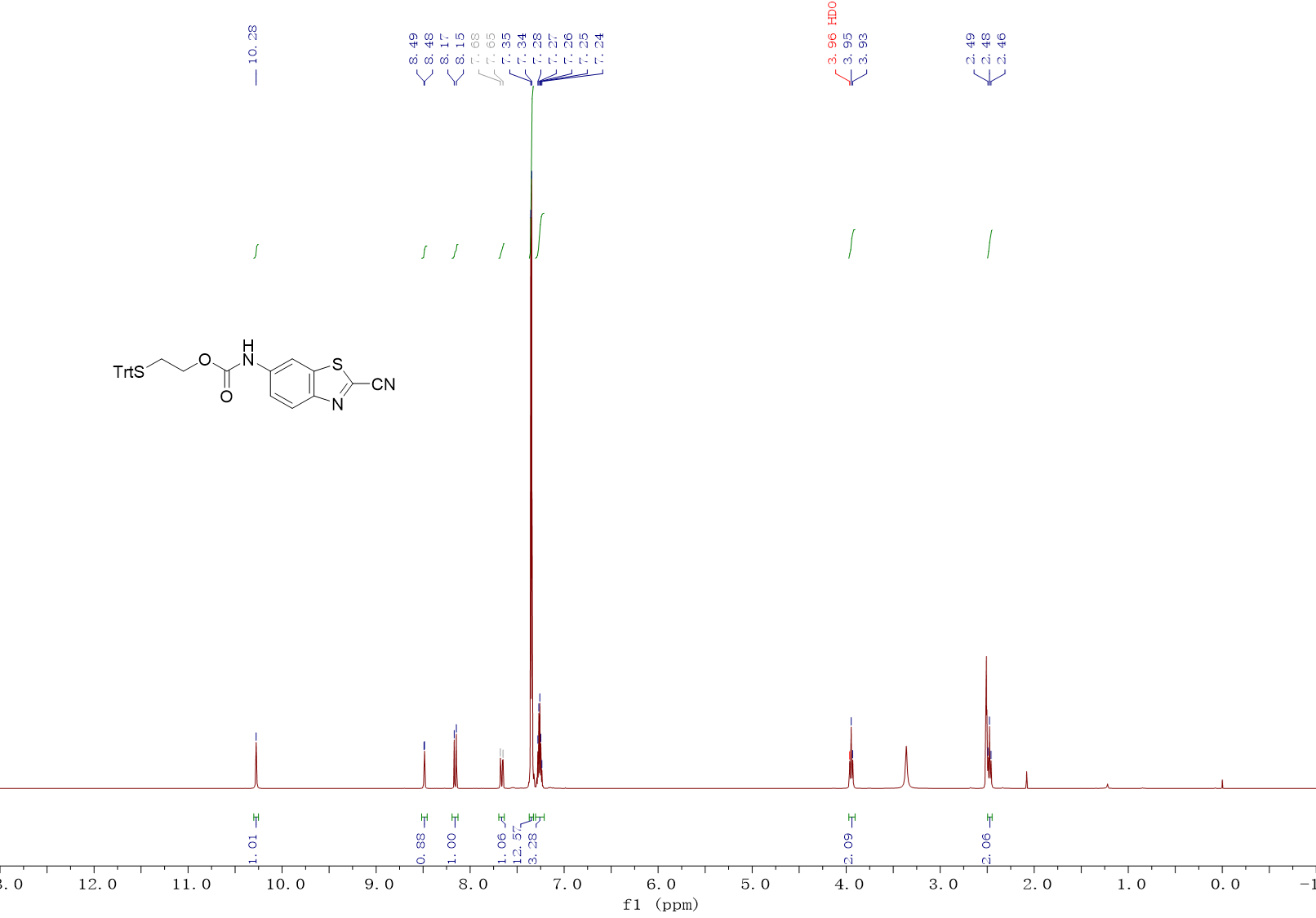


**Figure S32.** ^13^C-NMR spectra of compound **2** (DMSO-*d*_6_)


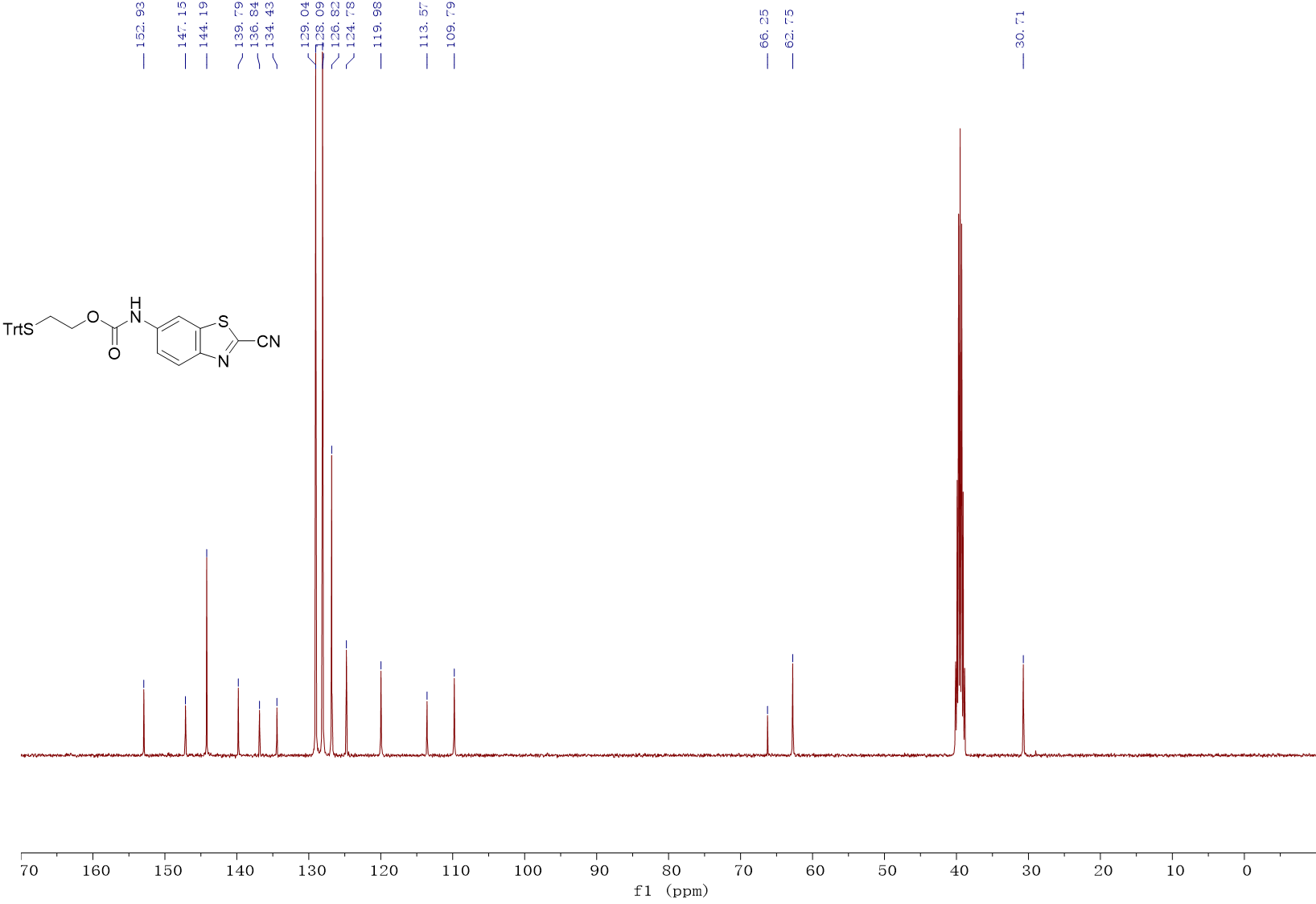


**Figure S33.** MALDI-TOF Spectrum of compound **2**


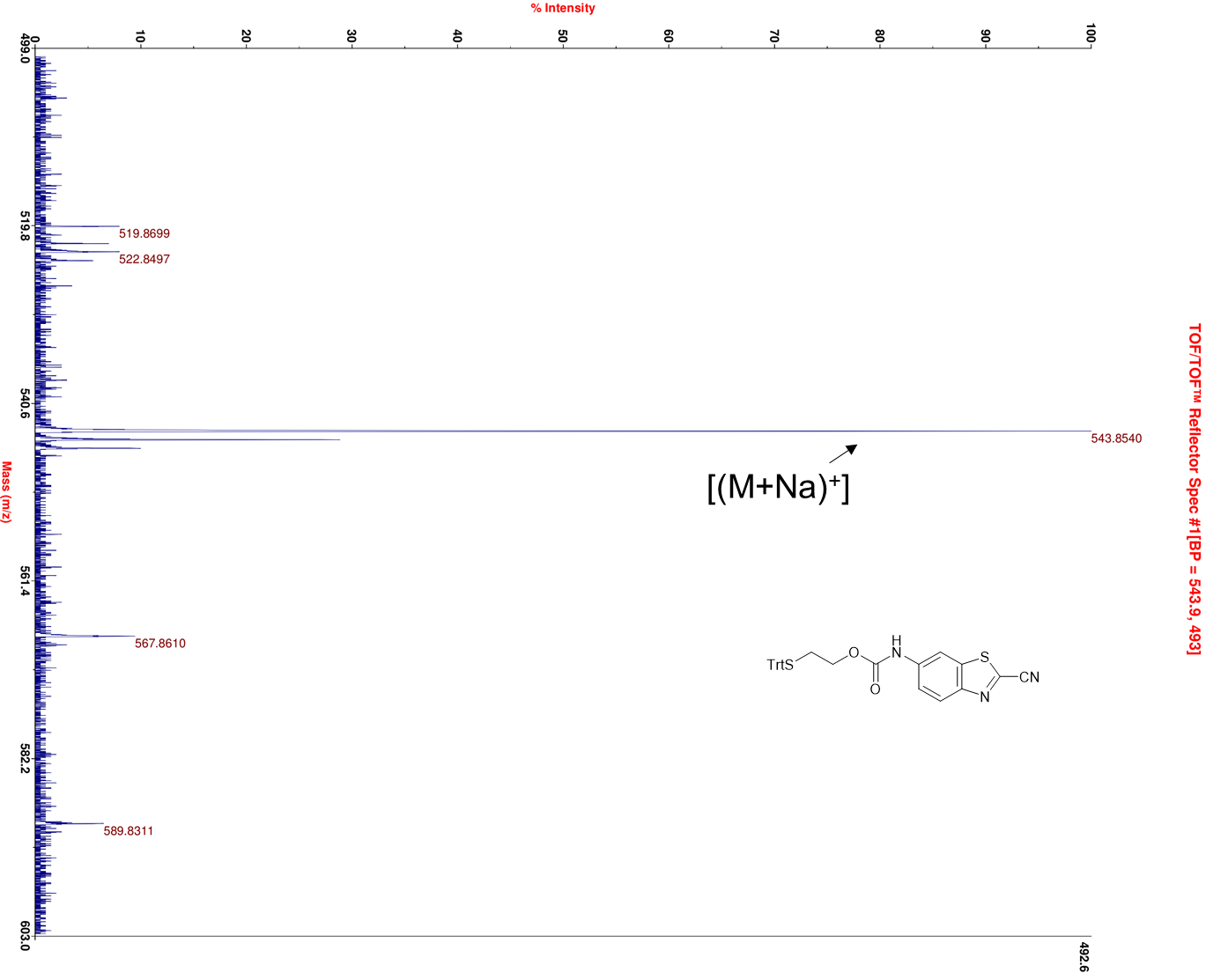


**Figure S34.**^1^H-NMR spectra of compound **3** (DMSO-*d*_6_)


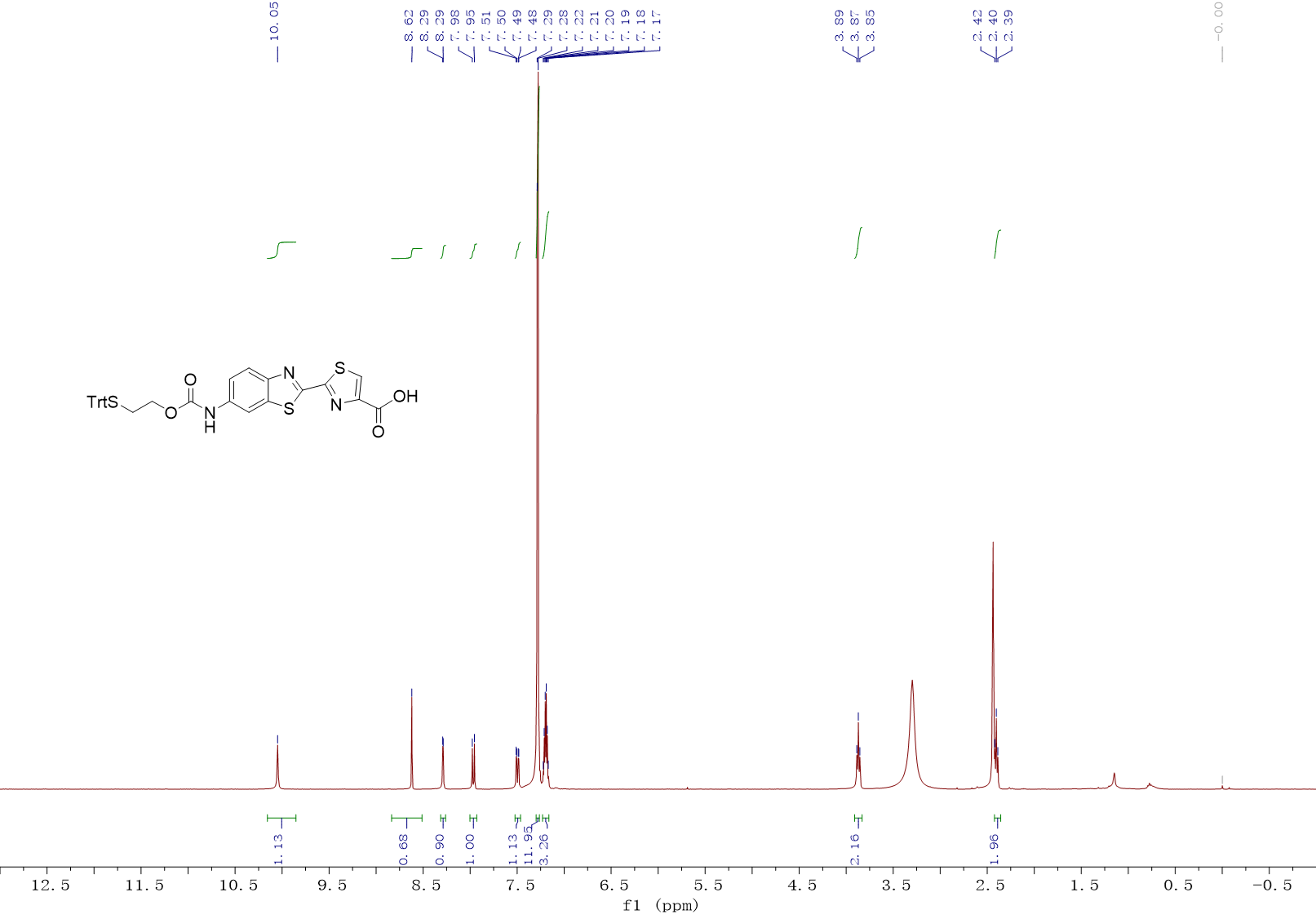


**Figure S35.**^13^C-NMR spectra of compound **3** (DMSO-*d*_6_)


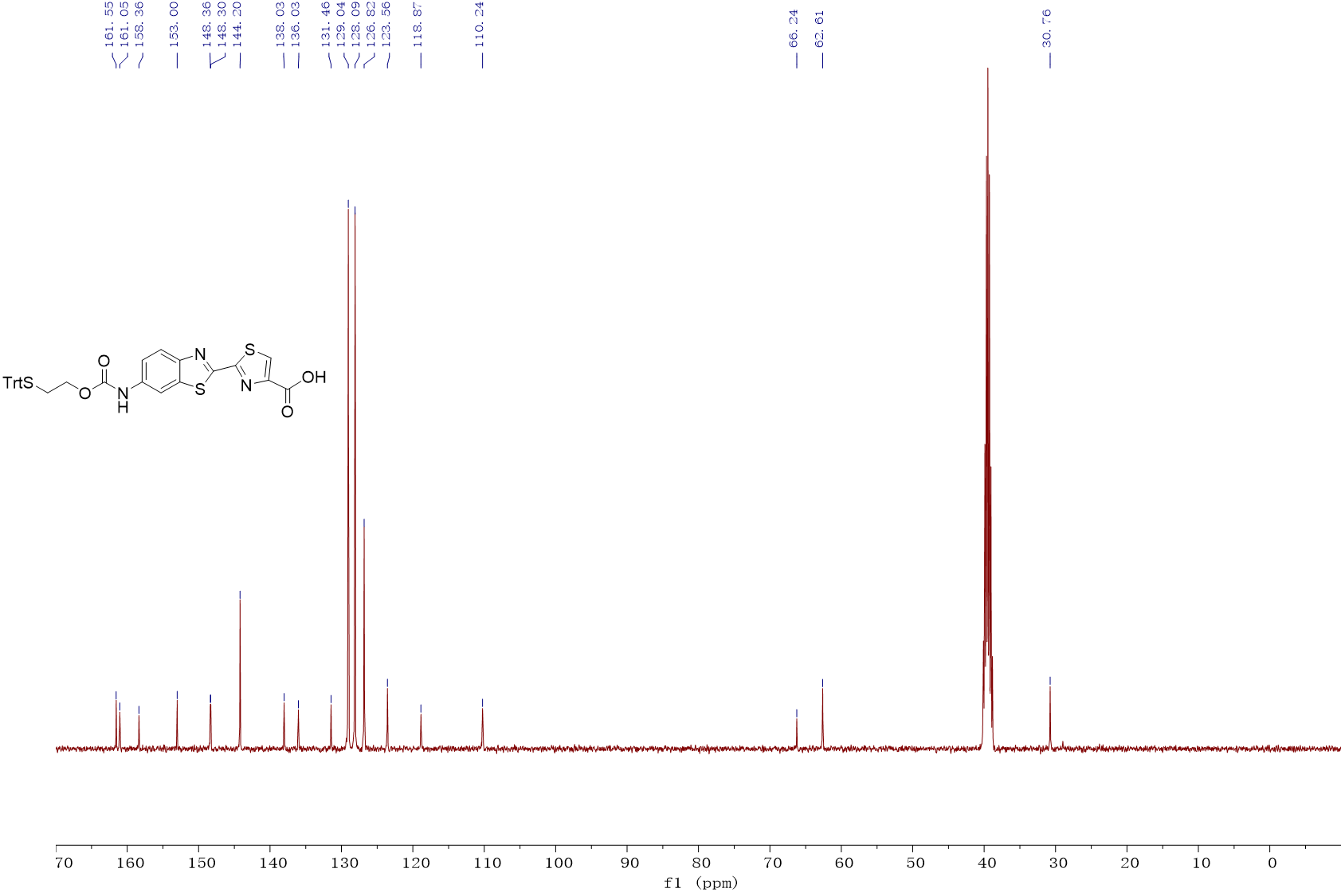


**Figure S36.** HRMS Spectrum of compound **3**


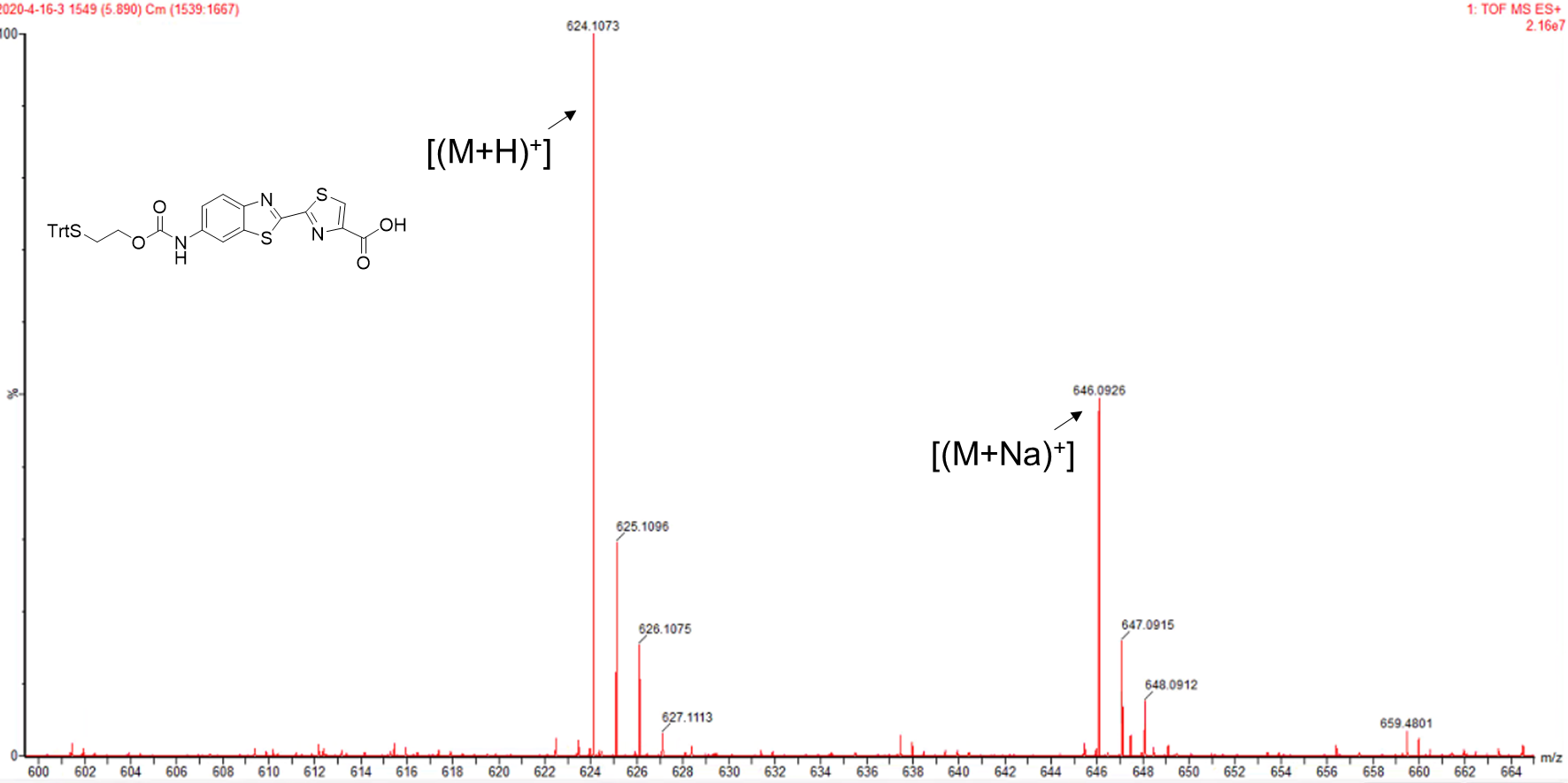


**Figure S37.** ^1^H-NMR spectra of compound **6** (DMSO-*d*_6_)


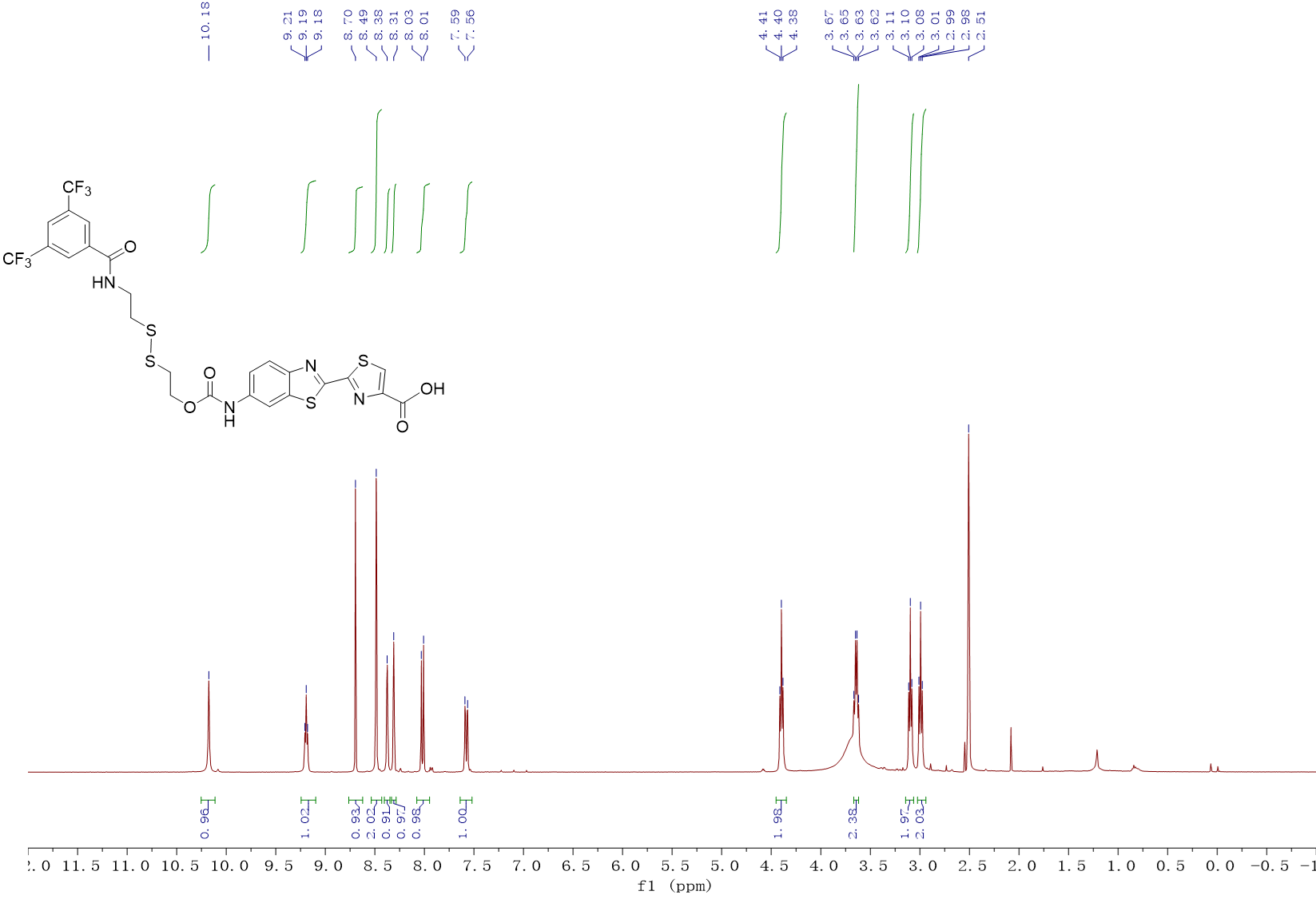


**Figure S38.** ^13^C-NMR spectra of compound **6** (DMSO-*d*_6_)


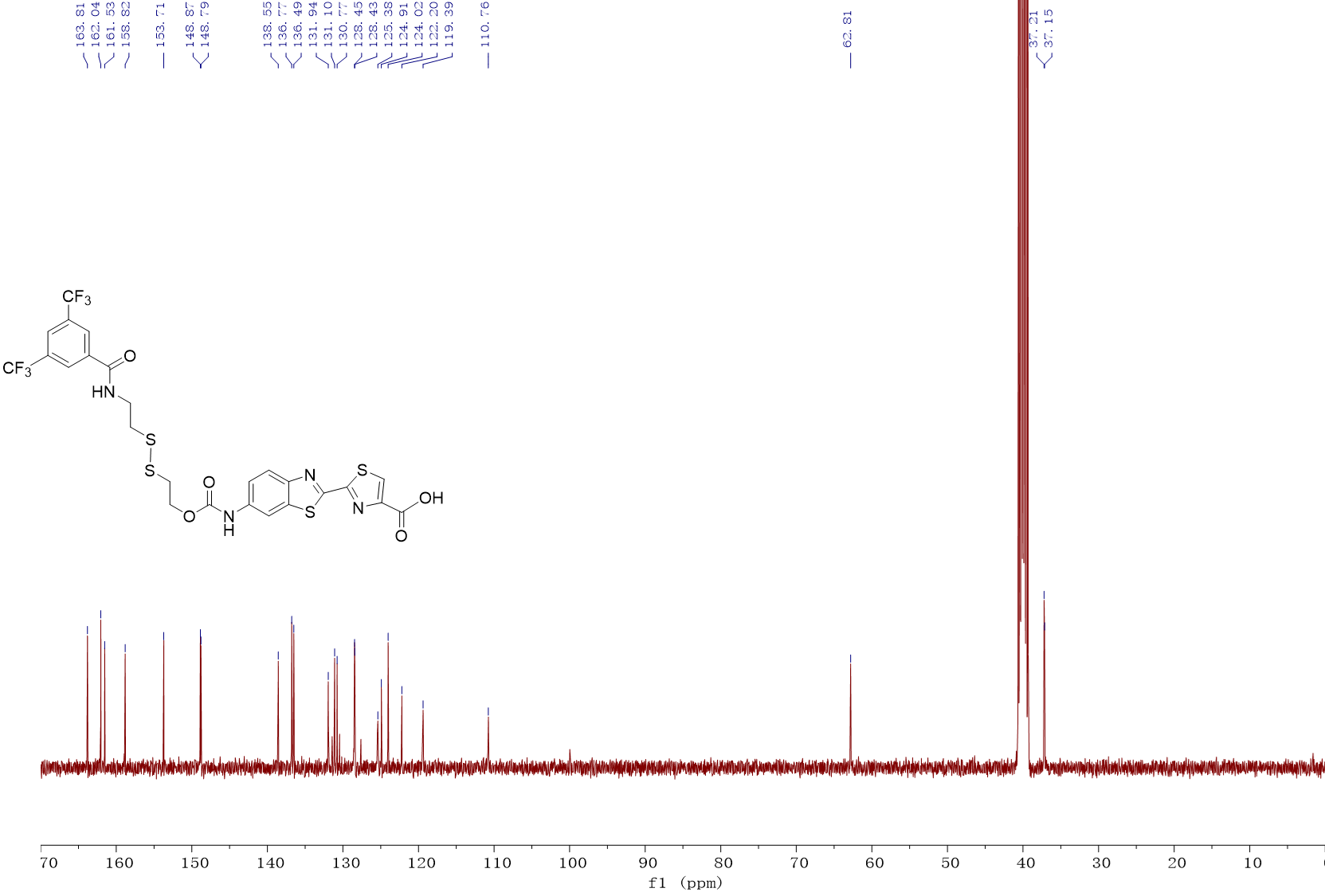


**Figure S39.** HRMS Spectrum of compound **6**


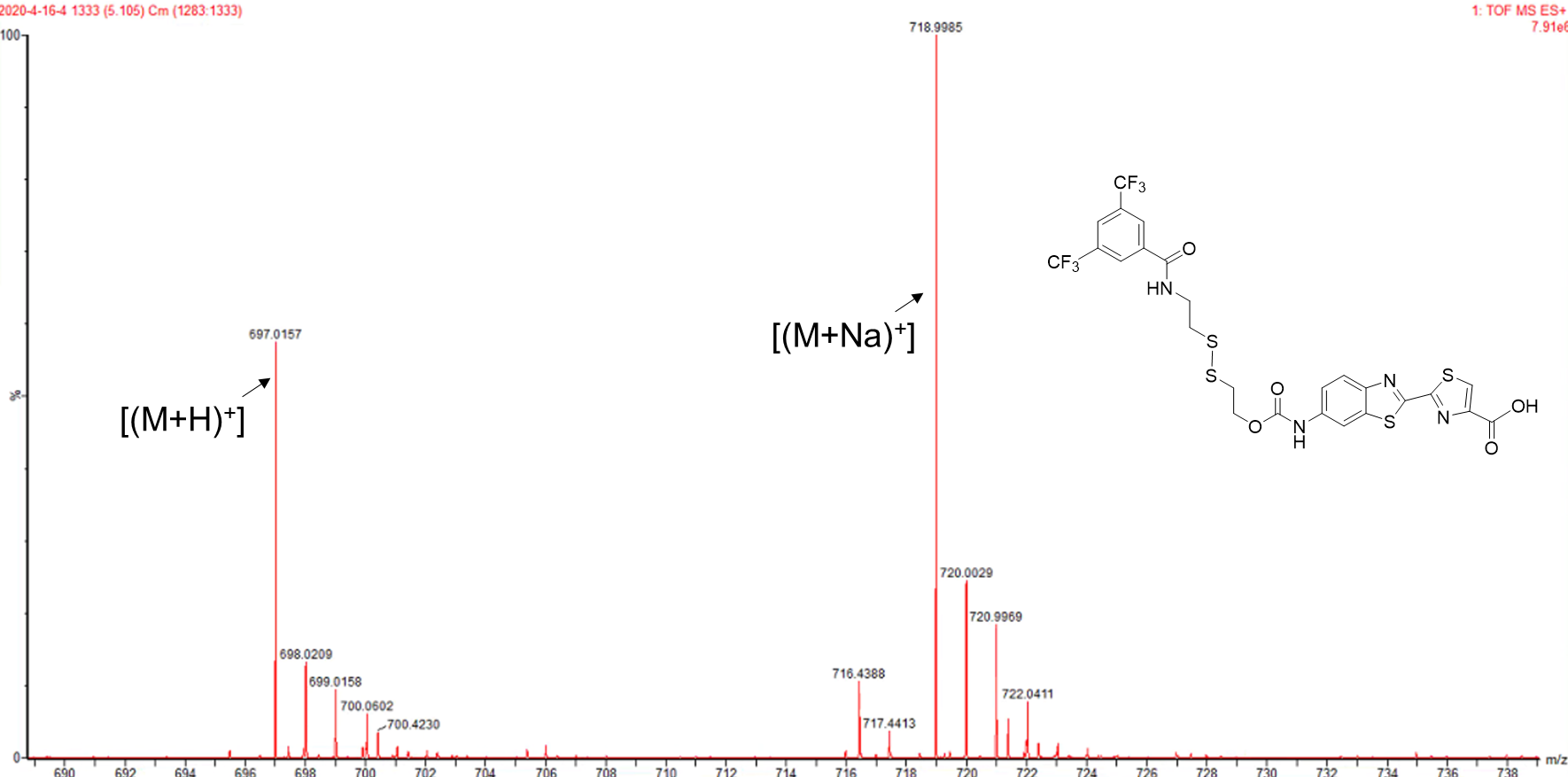


**Figure S40.** ^1^H-NMR spectra of compound **1-c** (DMSO-*d*_6_)


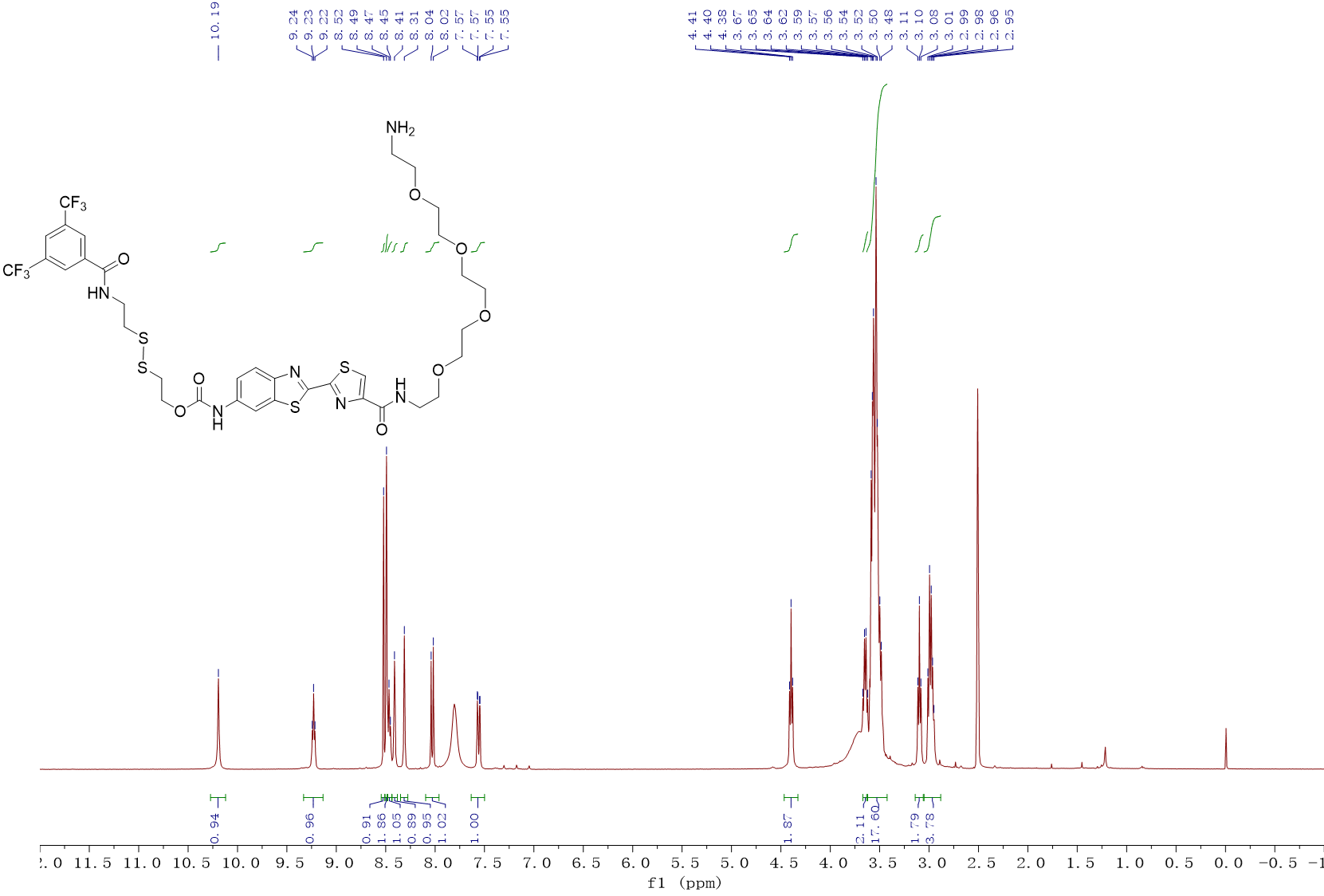


**Figure S41.** ^13^C-NMR spectra of compound **1-c** (DMSO-*d*_6_)


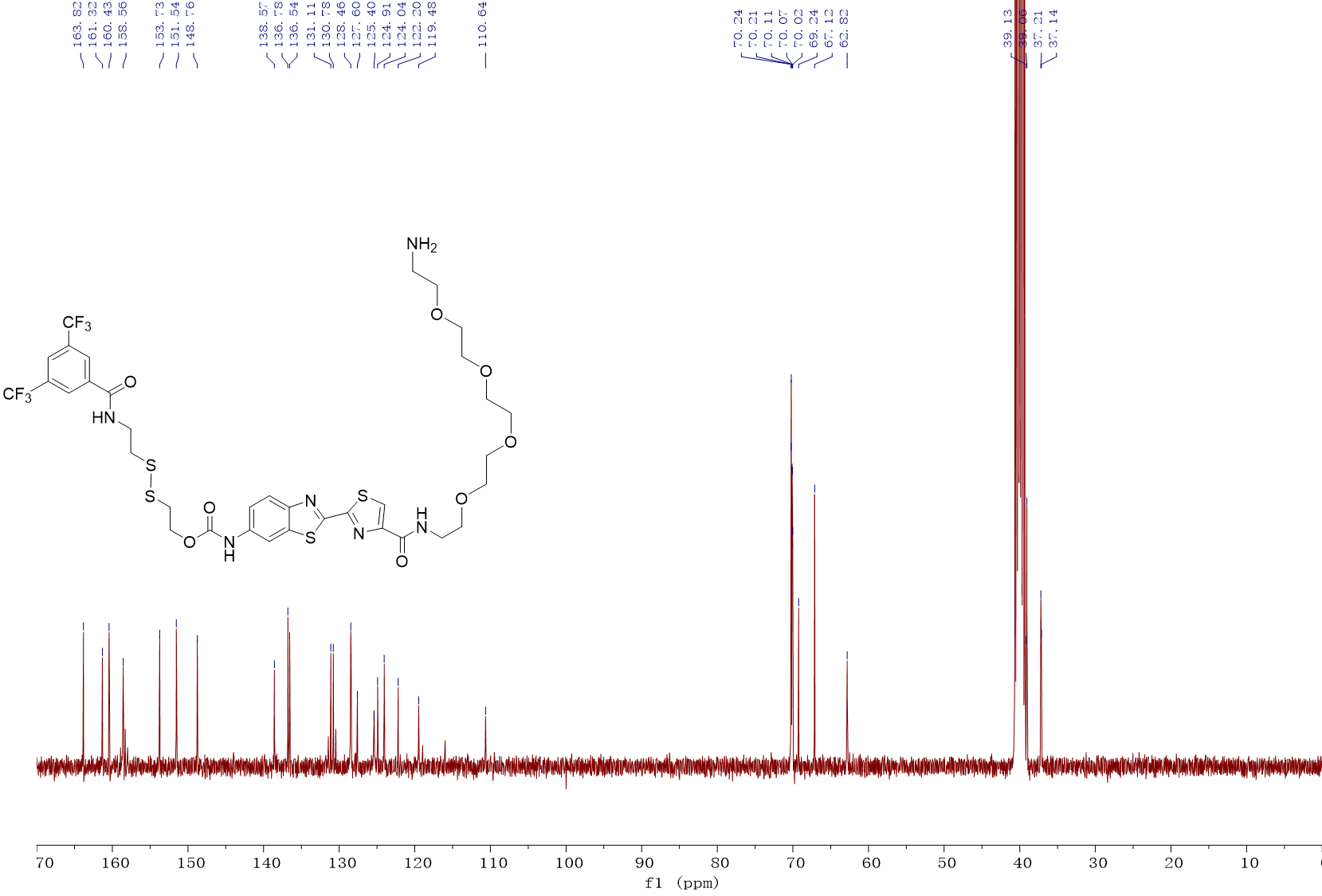


**Figure S42.** HRMS Spectrum of compound **1-c**


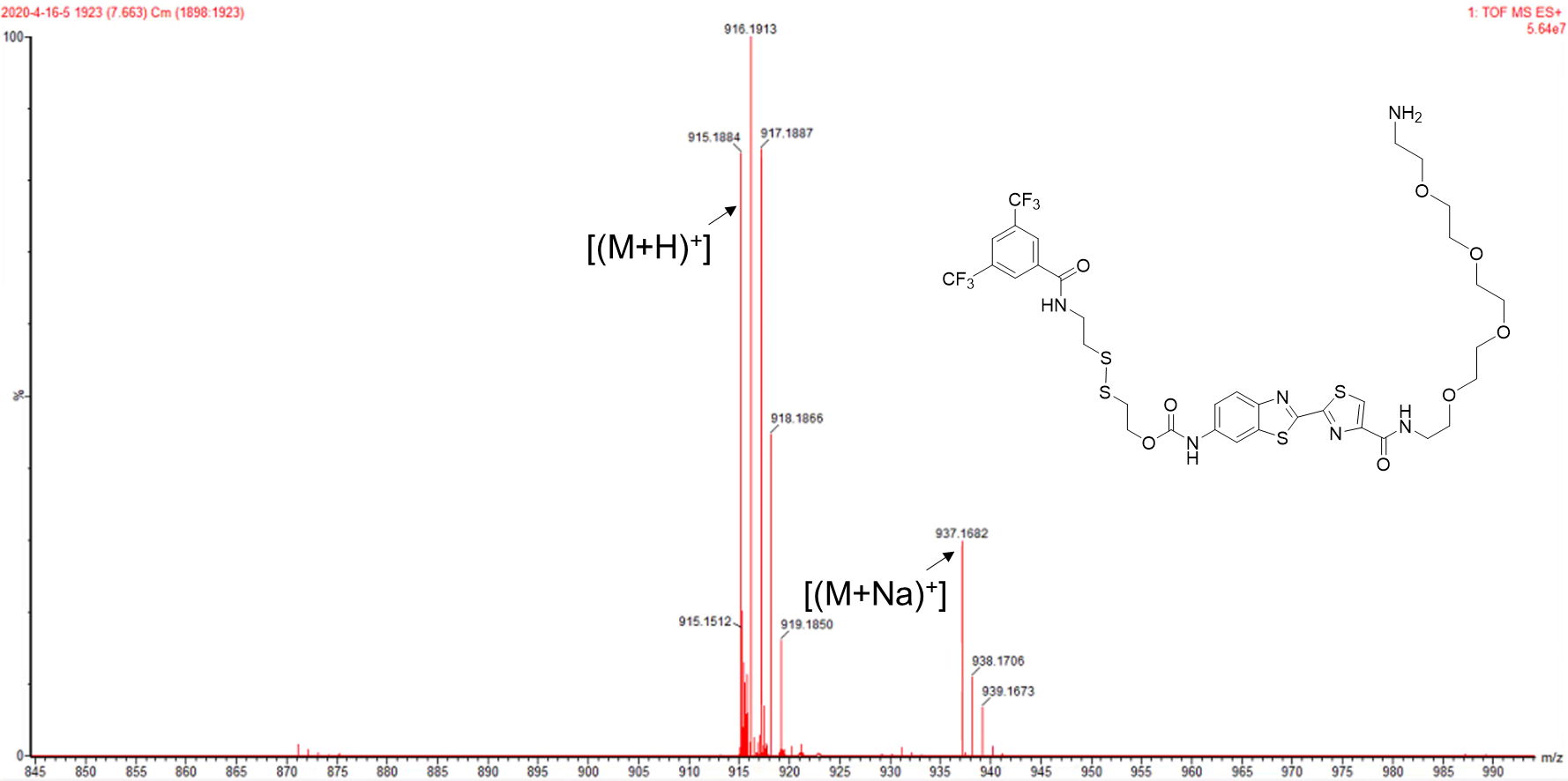


**Figure S43.** ^1^H-NMR spectra of compound **7** (DMSO-*d*_6_)


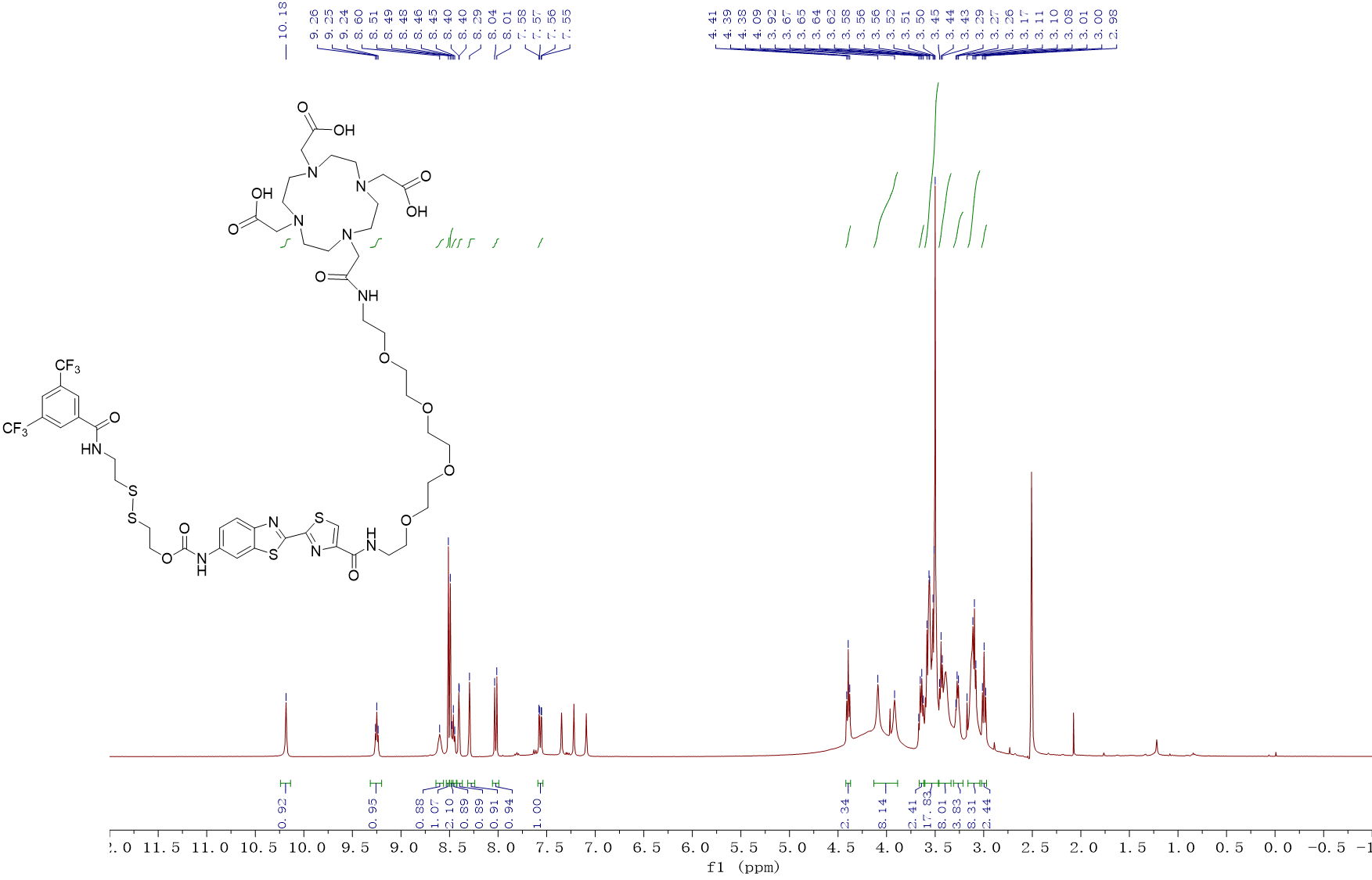


**Figure S44.** ^13^C-NMR spectra of compound **7** (DMSO-*d*_6_)


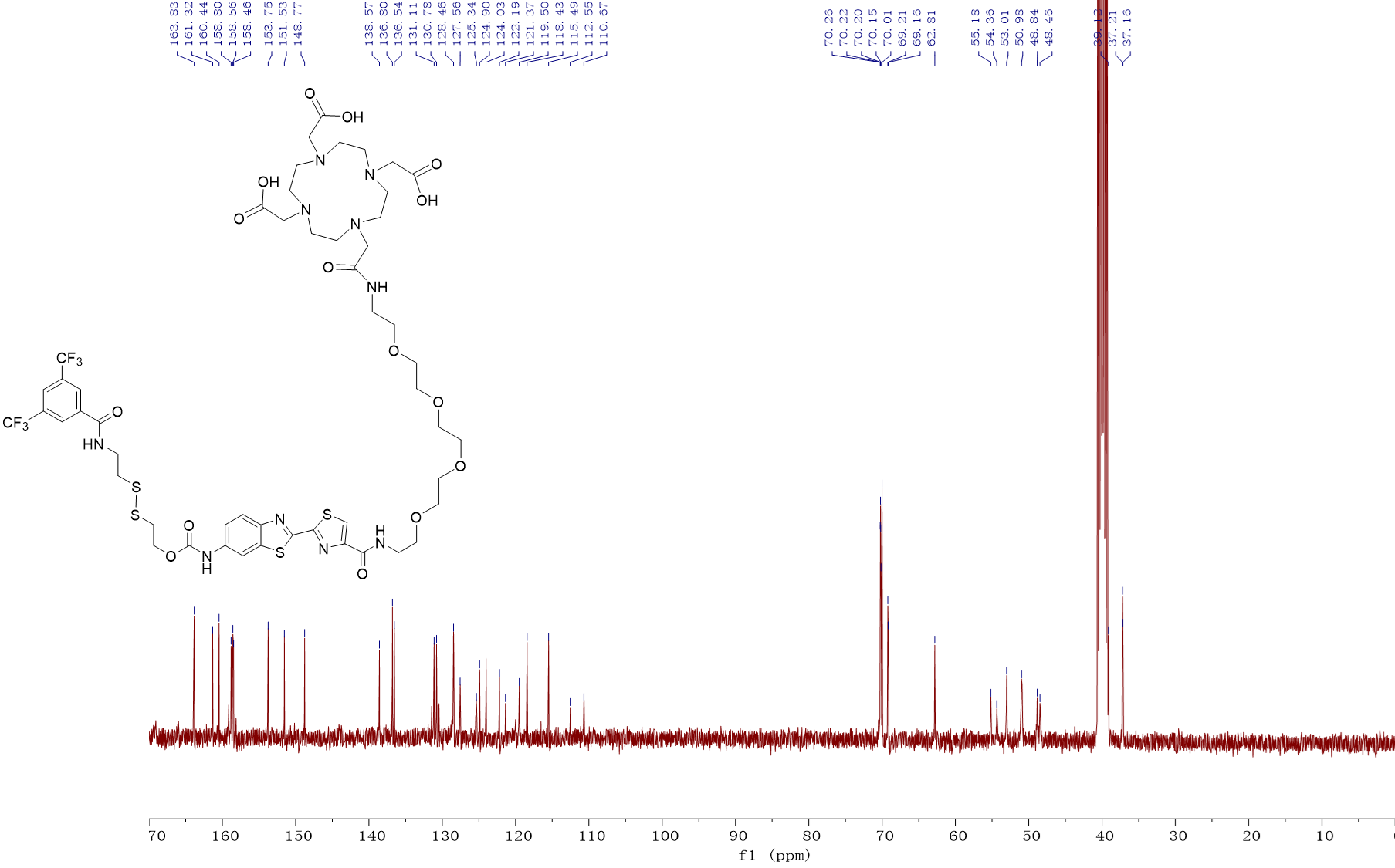


**Figure S45.** MALDI-TOF Spectrum of compound **7**


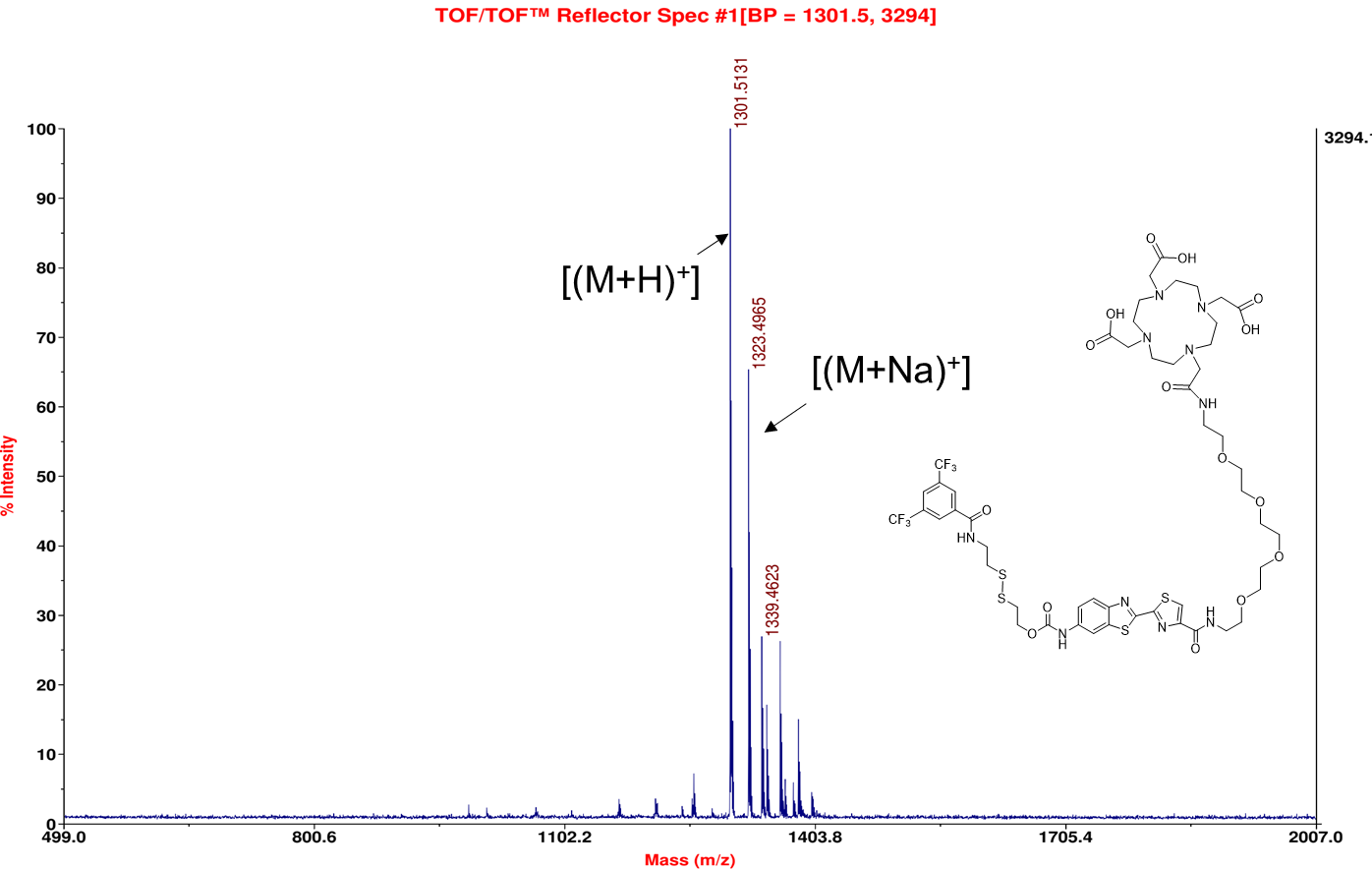


**Figure S46.** HRMS Spectrum of compound **1-Gd**


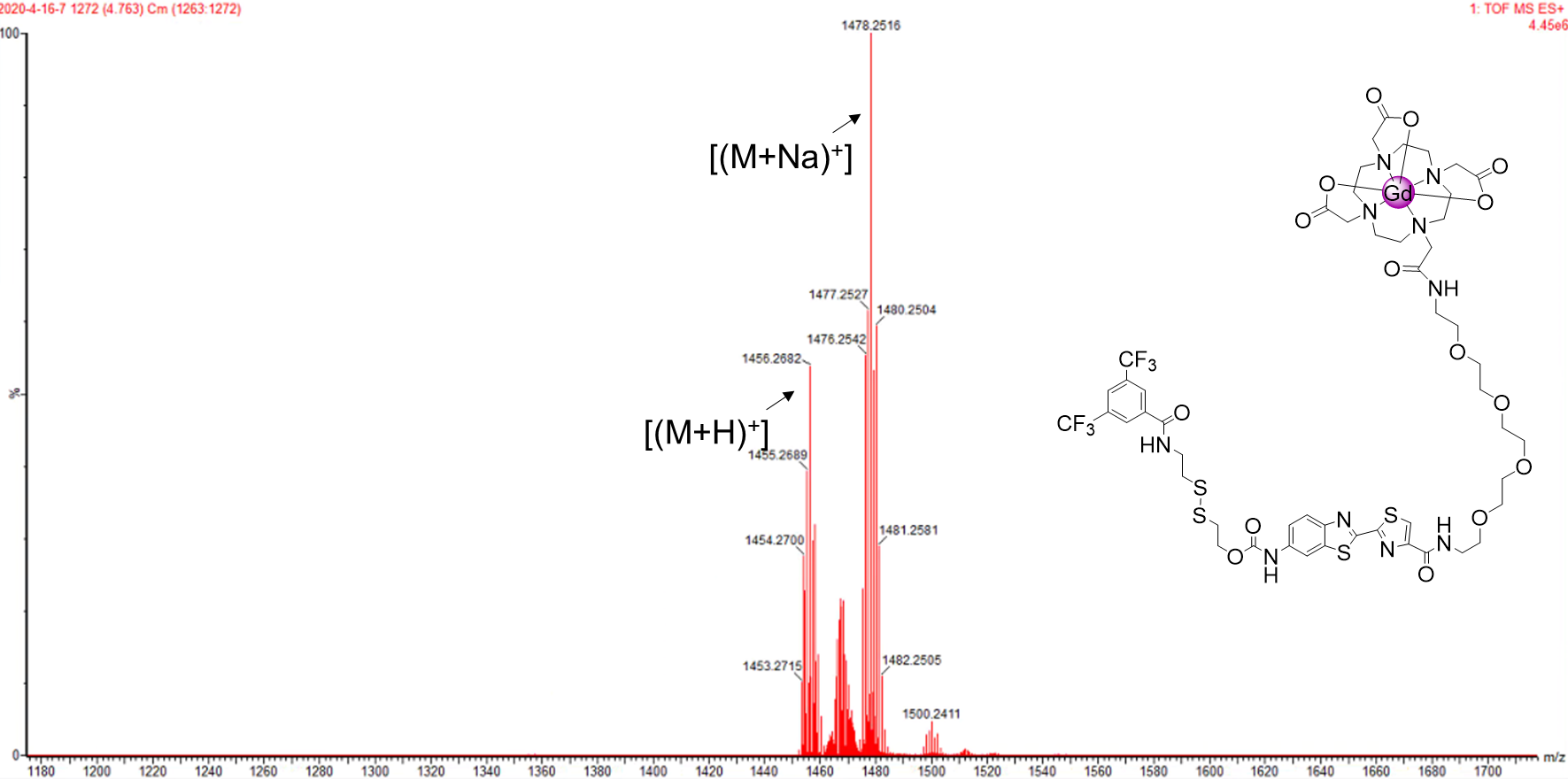


**Figure S47.** ^1^H-NMR spectra of compound **8** (DMSO-*d*_6_)


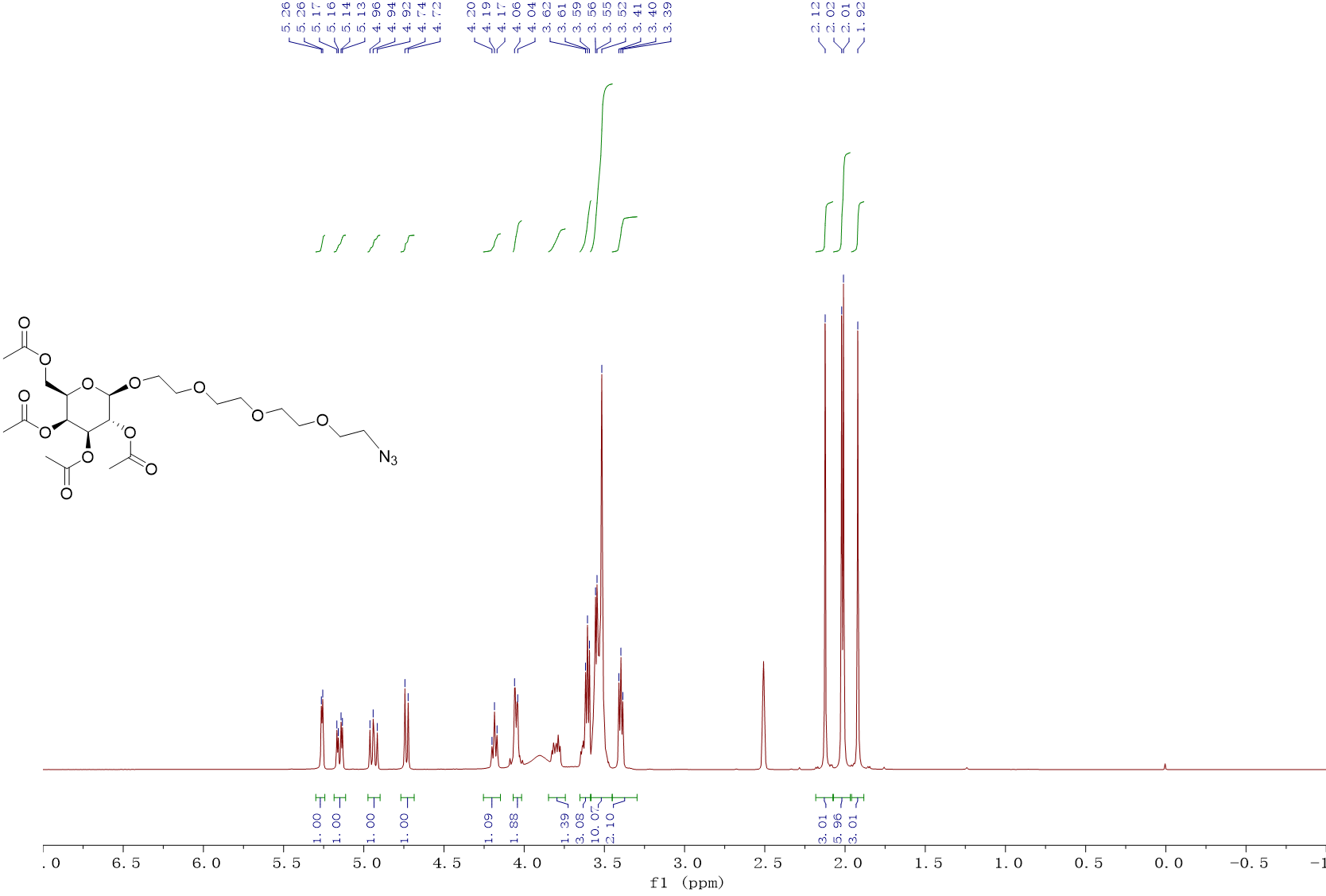


**Figure S48.** ^13^C-NMR spectra of compound **8** (DMSO-*d*_6_)


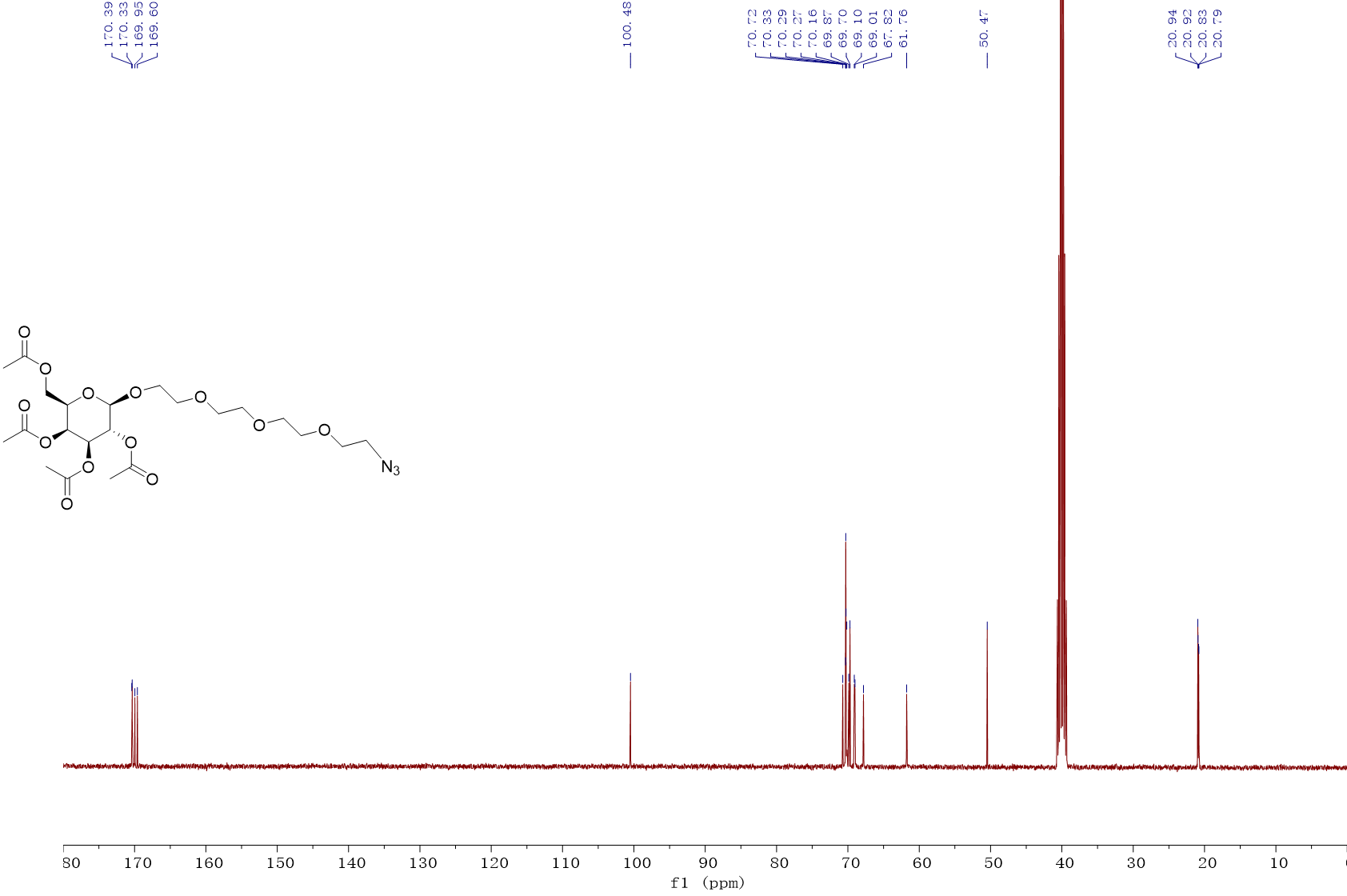


**Figure S49.** MALDI-TOF Spectrum of compound **8**


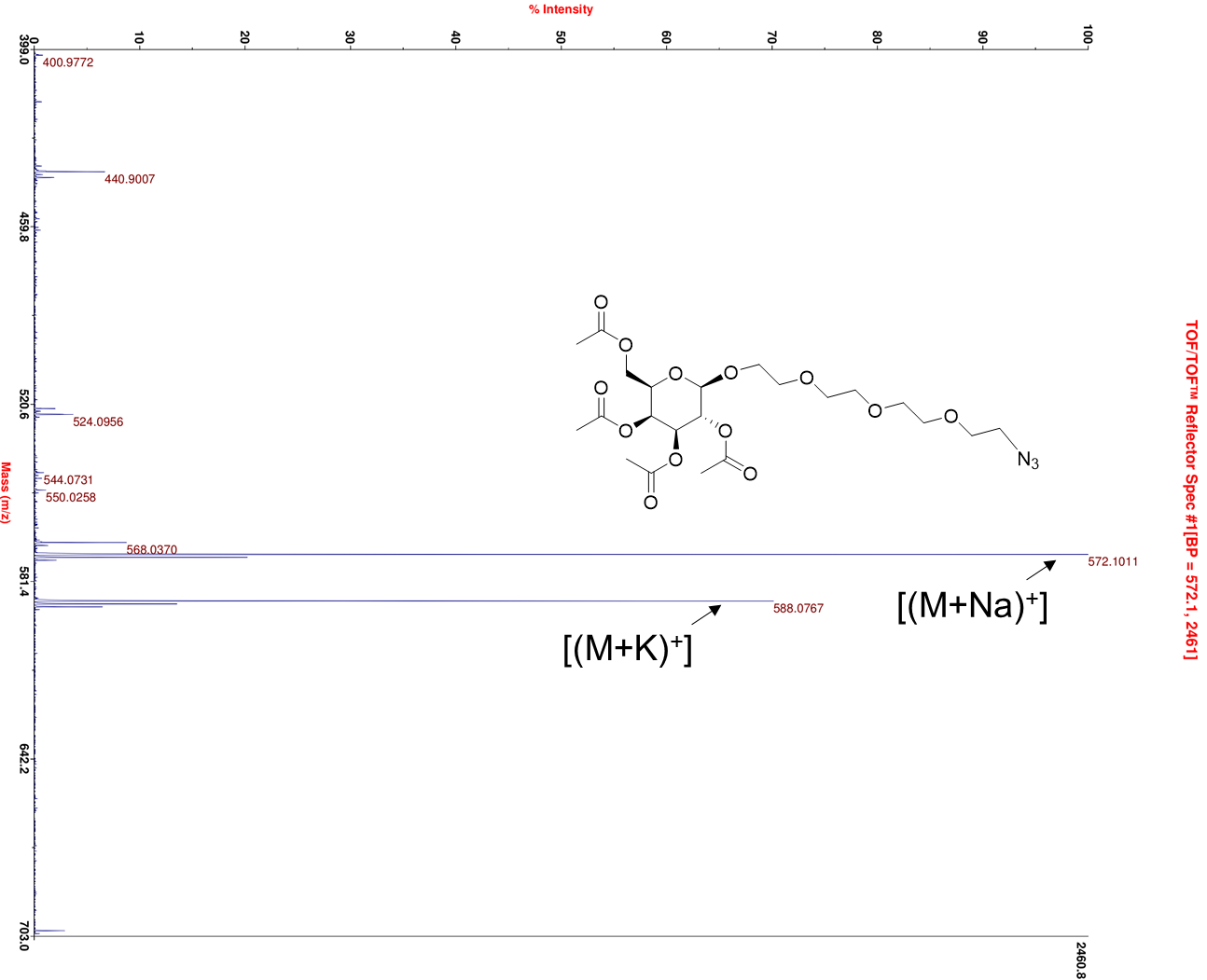


**Figure S50.** ^1^H-NMR spectra of compound **10** (DMSO-*d*_6_)


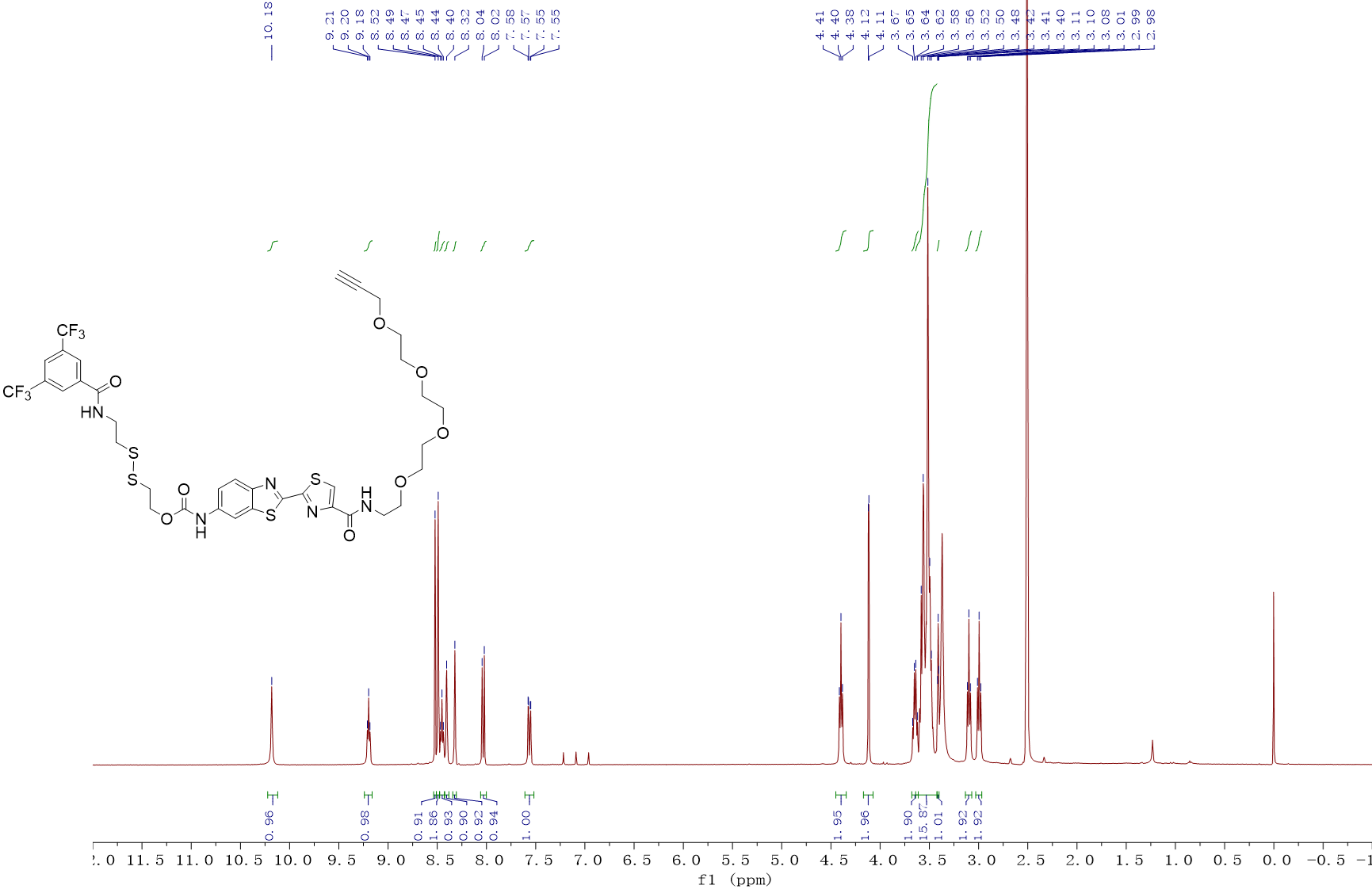


**Figure S51.** ^13^C-NMR spectra of compound **10** (DMSO-*d*_6_)


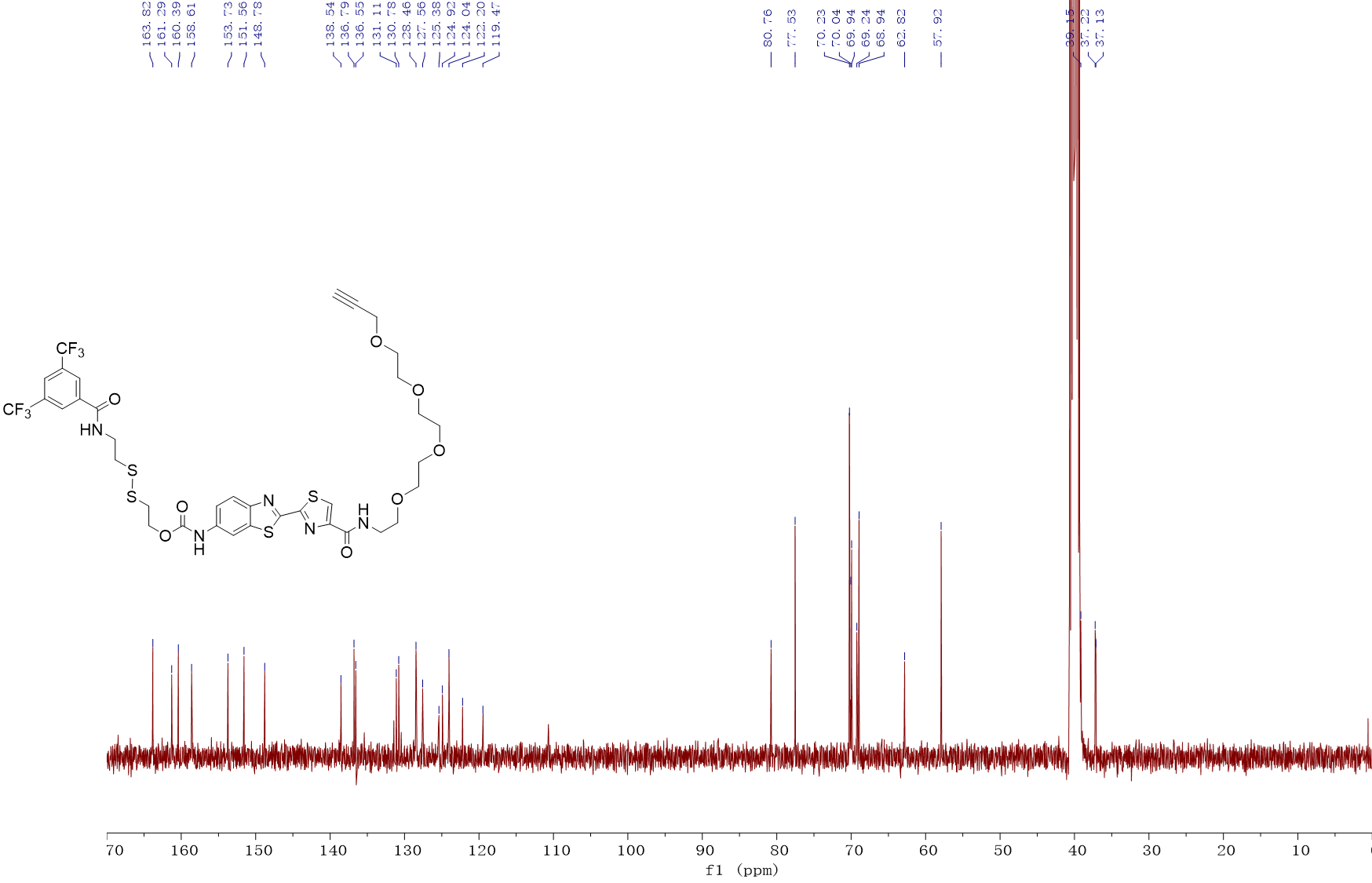


**Figure S52.** HRMS Spectrum of compound **10**


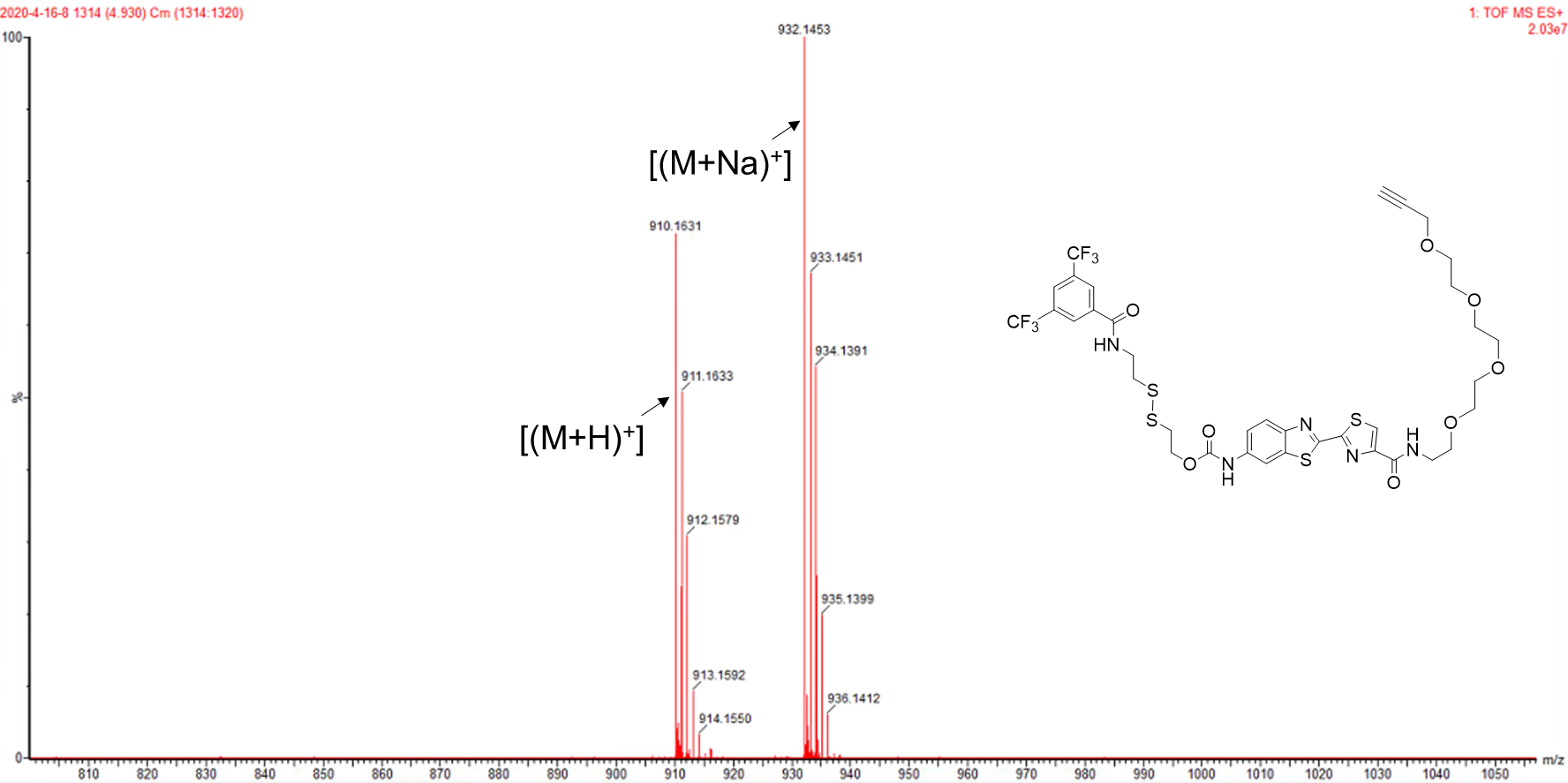


**Figure S53.** ^1^H-NMR spectra of compound **1-Gal** (DMSO-*d*_6_)


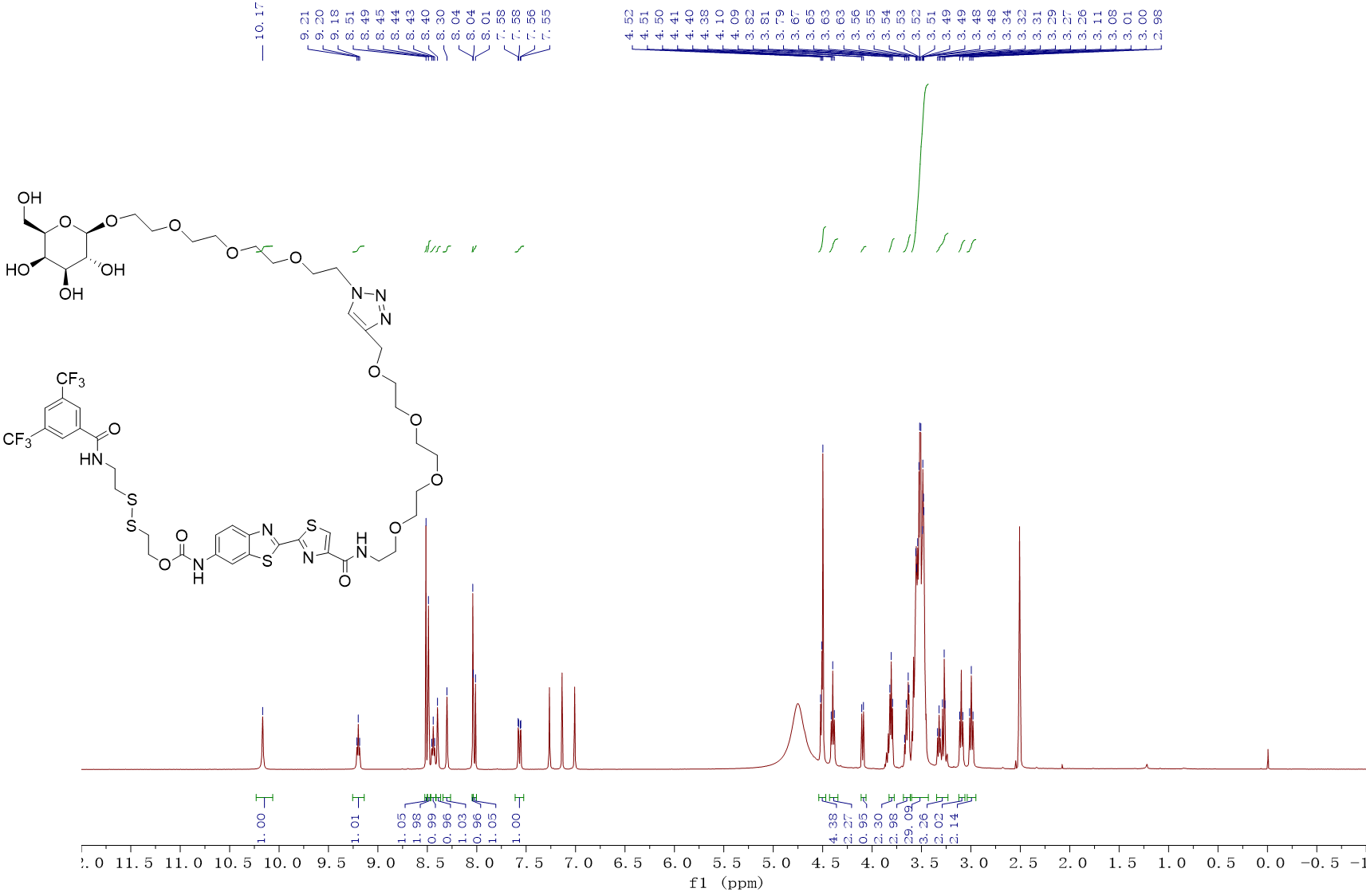


**Figure S54.** ^13^C-NMR spectra of compound **1-Gal** (DMSO-*d*_6_)


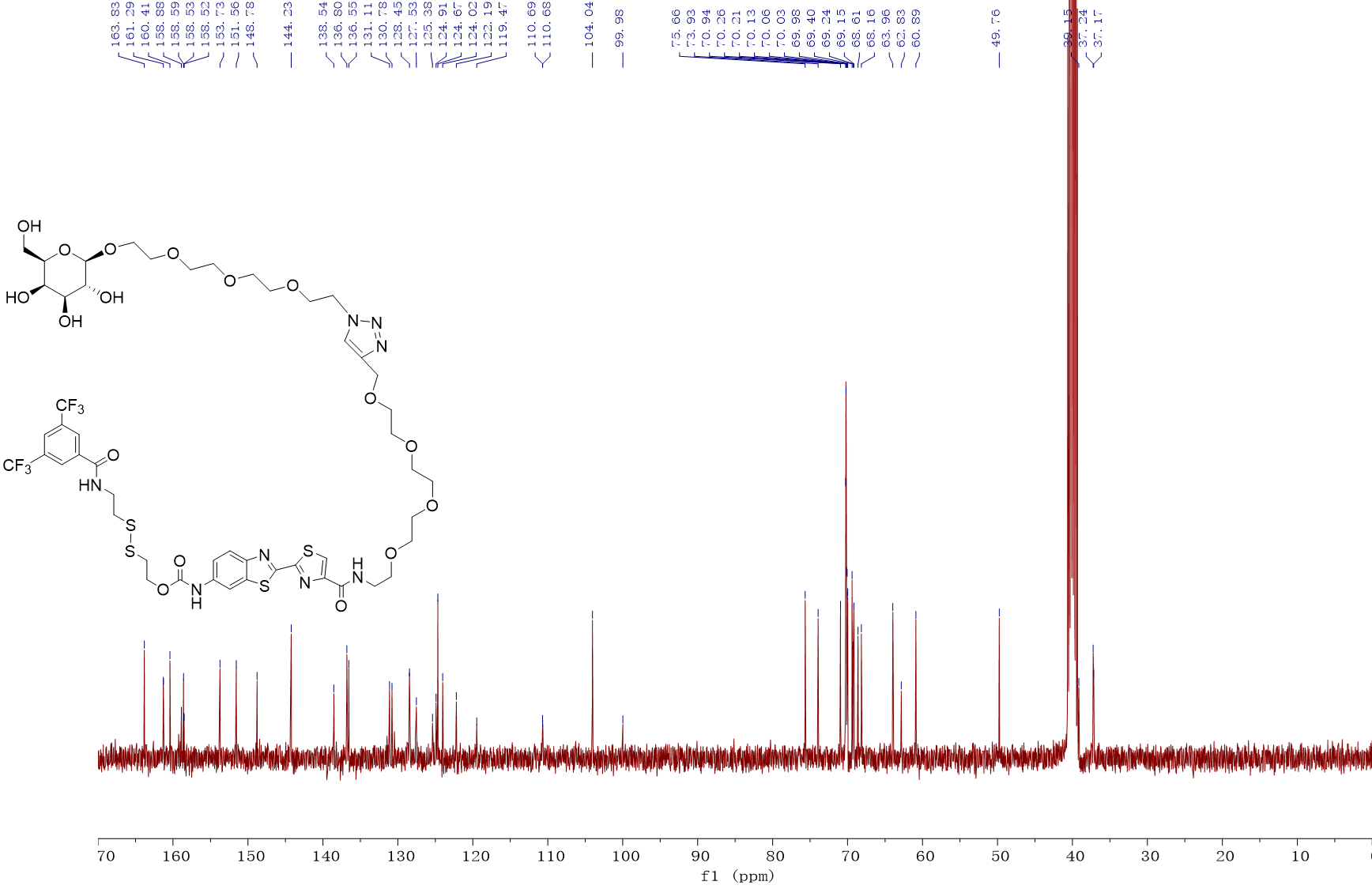


**Figure S55.** HRMS Spectrum of compound **1-Gal**


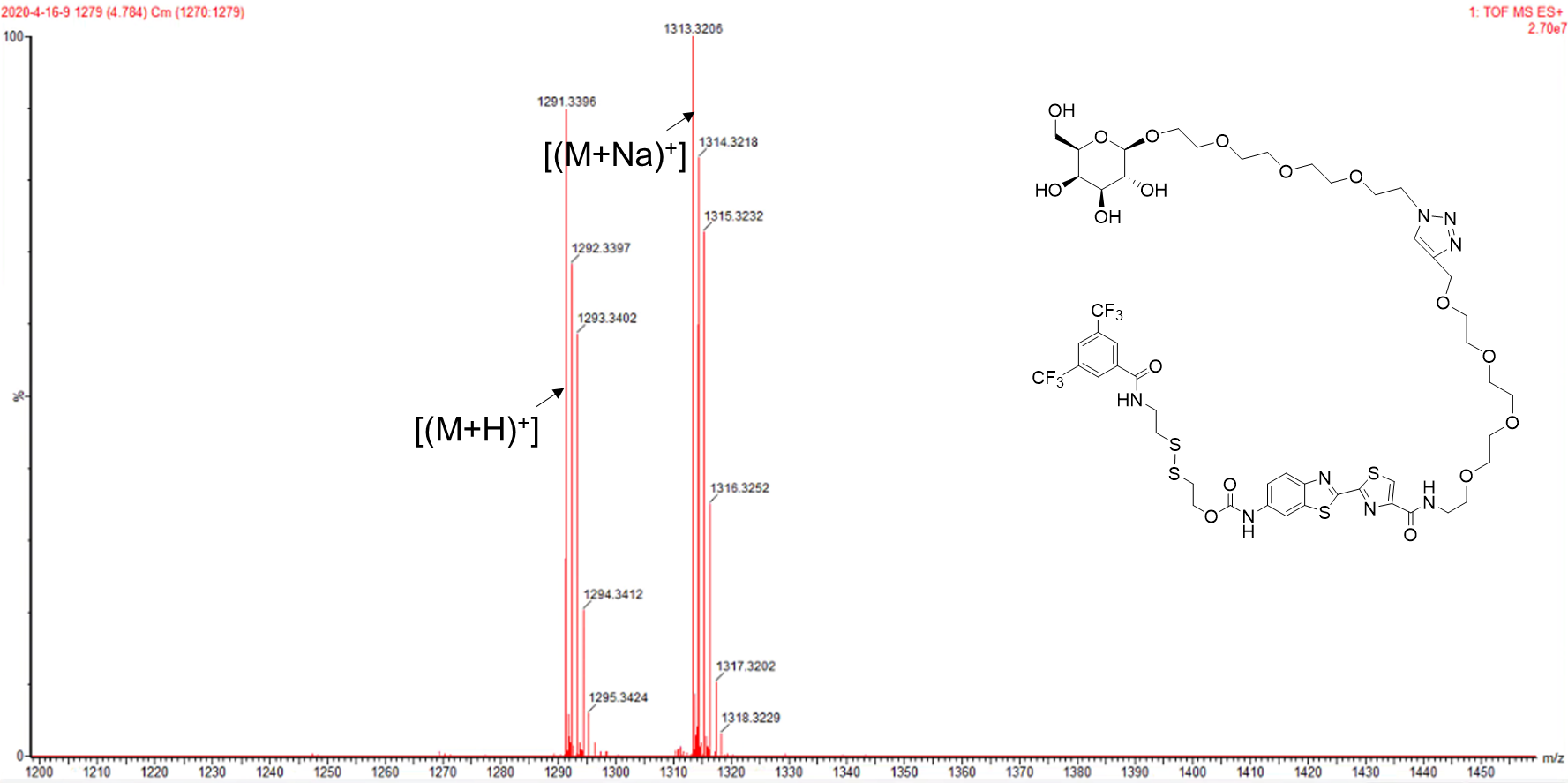


**Figure S56.** ^1^H-NMR spectra of compound **12** (DMSO-*d*_6_)


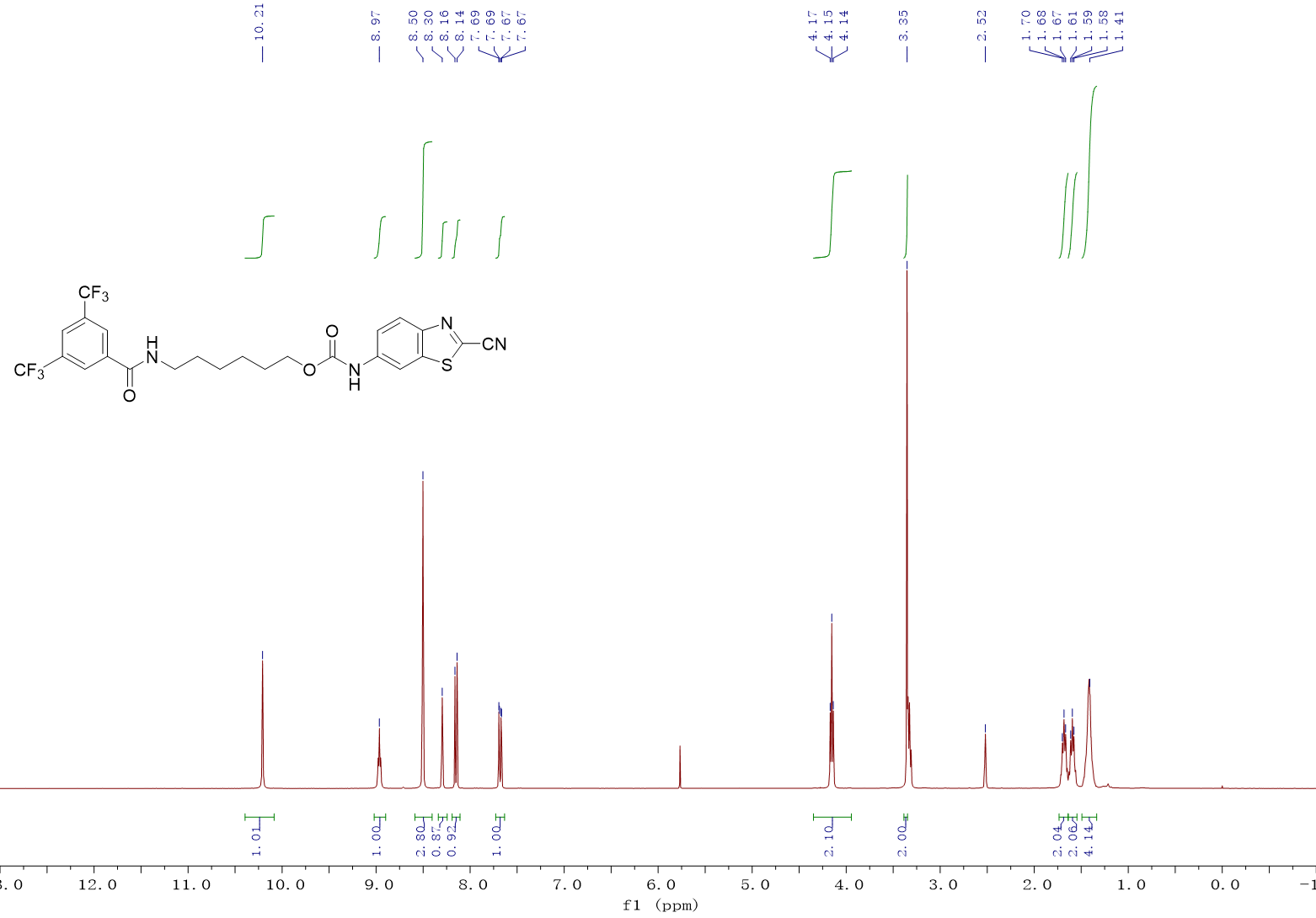


**Figure S57.** ^13^C-NMR spectra of compound **12** (DMSO-*d*_6_)


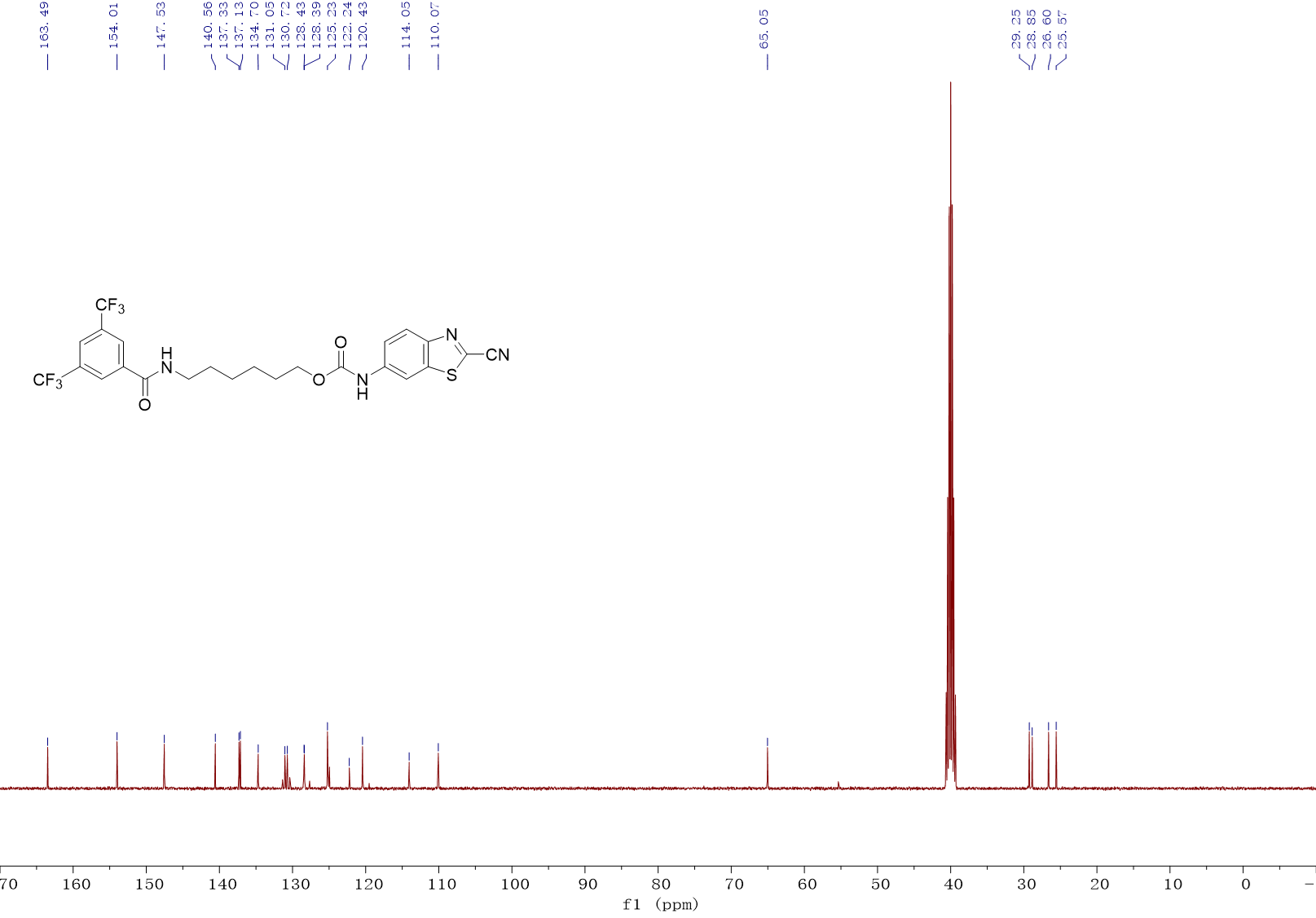


**Figure S58.** HRMS Spectrum of compound **12**


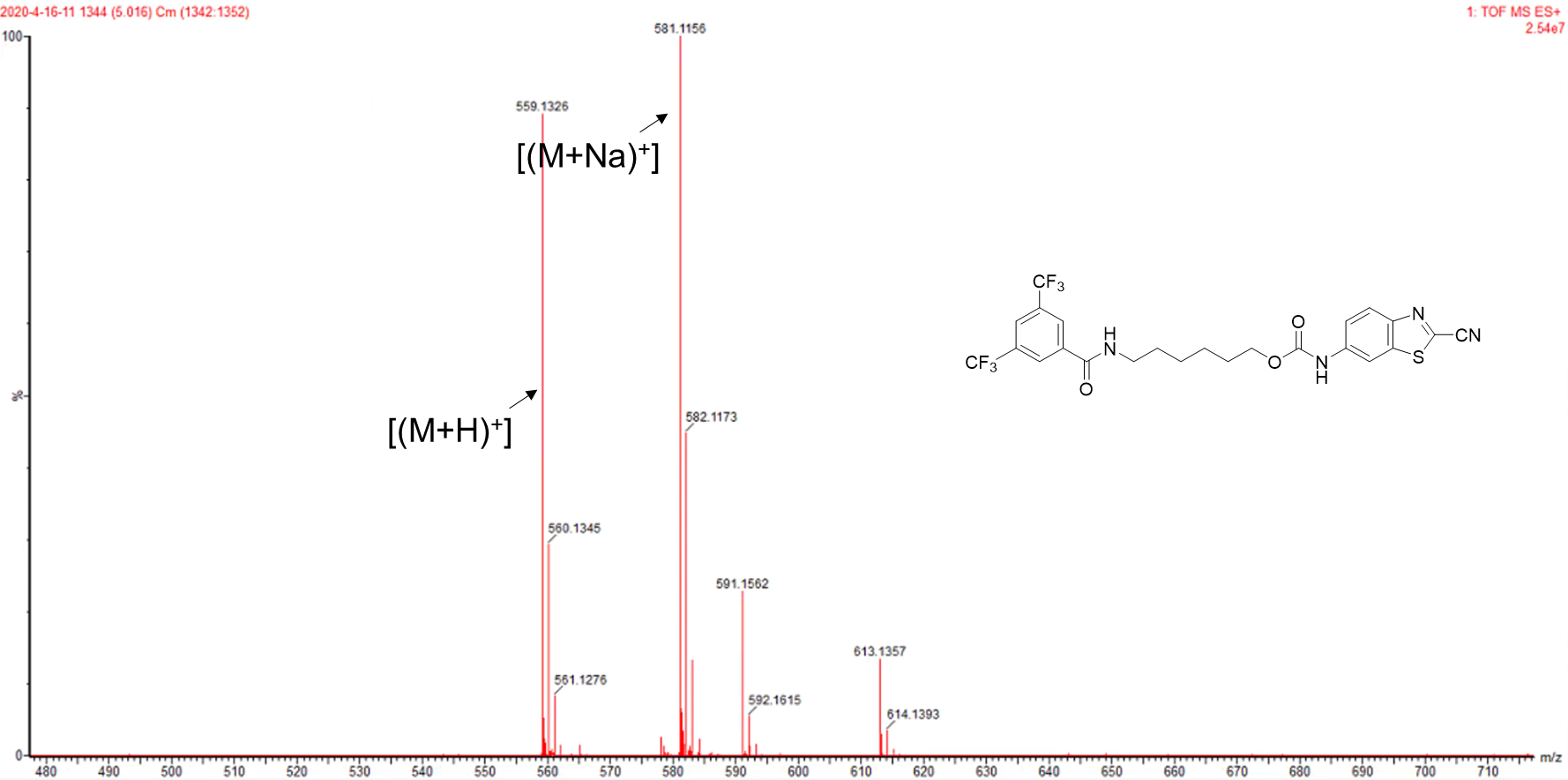


**Figure S59.** ^1^H-NMR spectra of compound **13** (DMSO-*d*_6_)


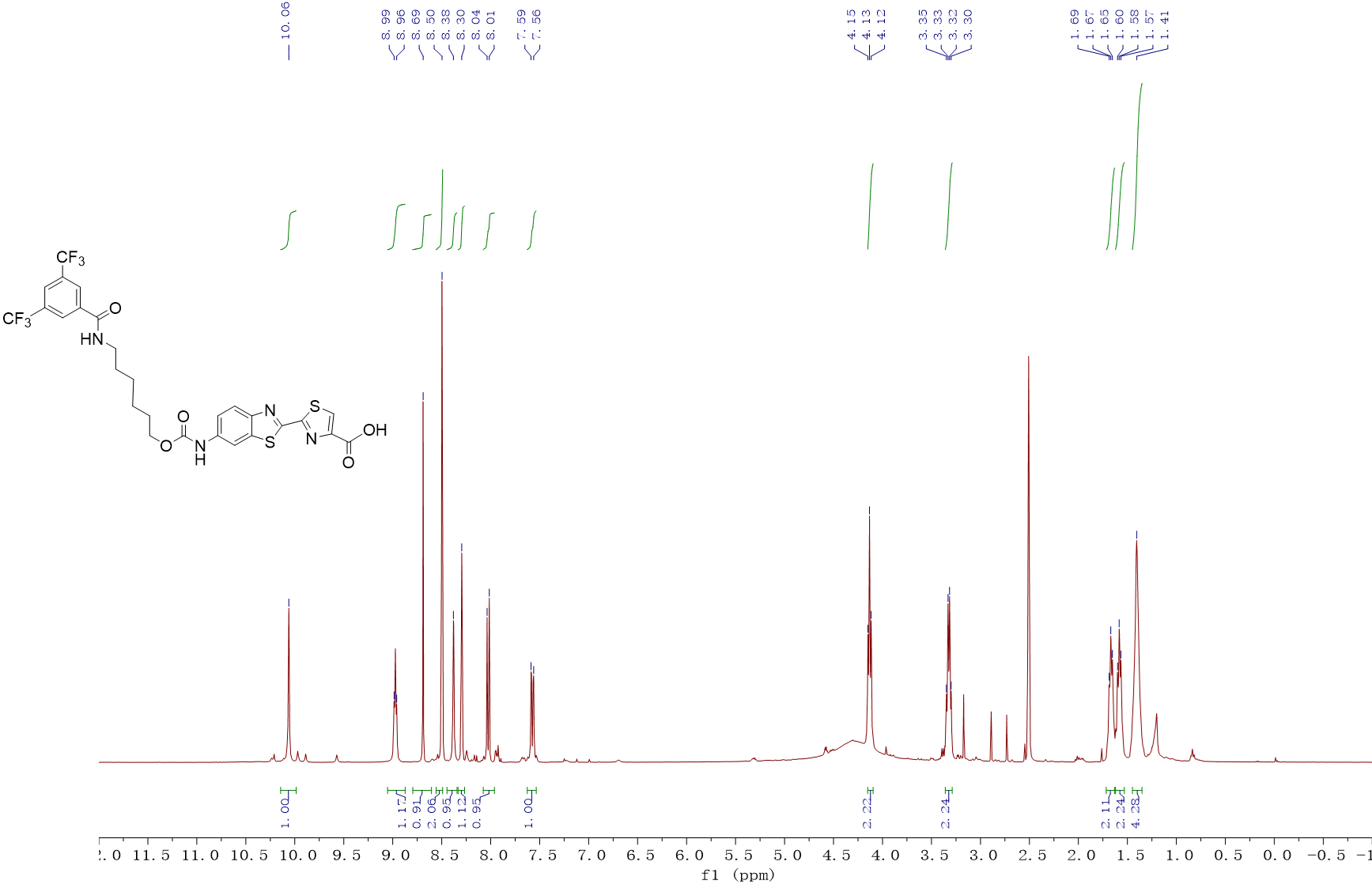


**Figure S60.** ^13^C-NMR spectra of compound **13** (DMSO-*d*_6_)


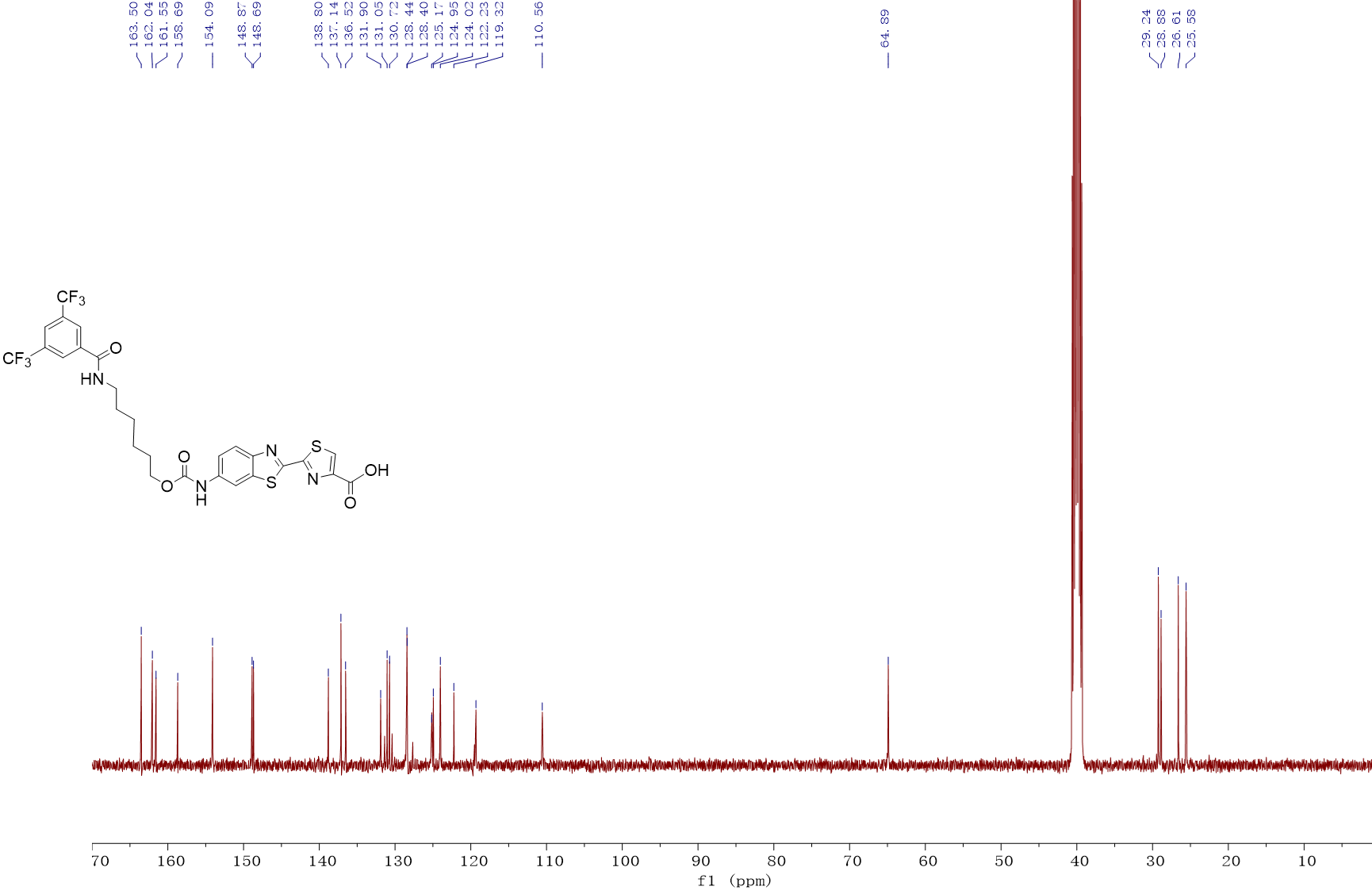


**Figure S61.** HRMS Spectrum of compound **13**


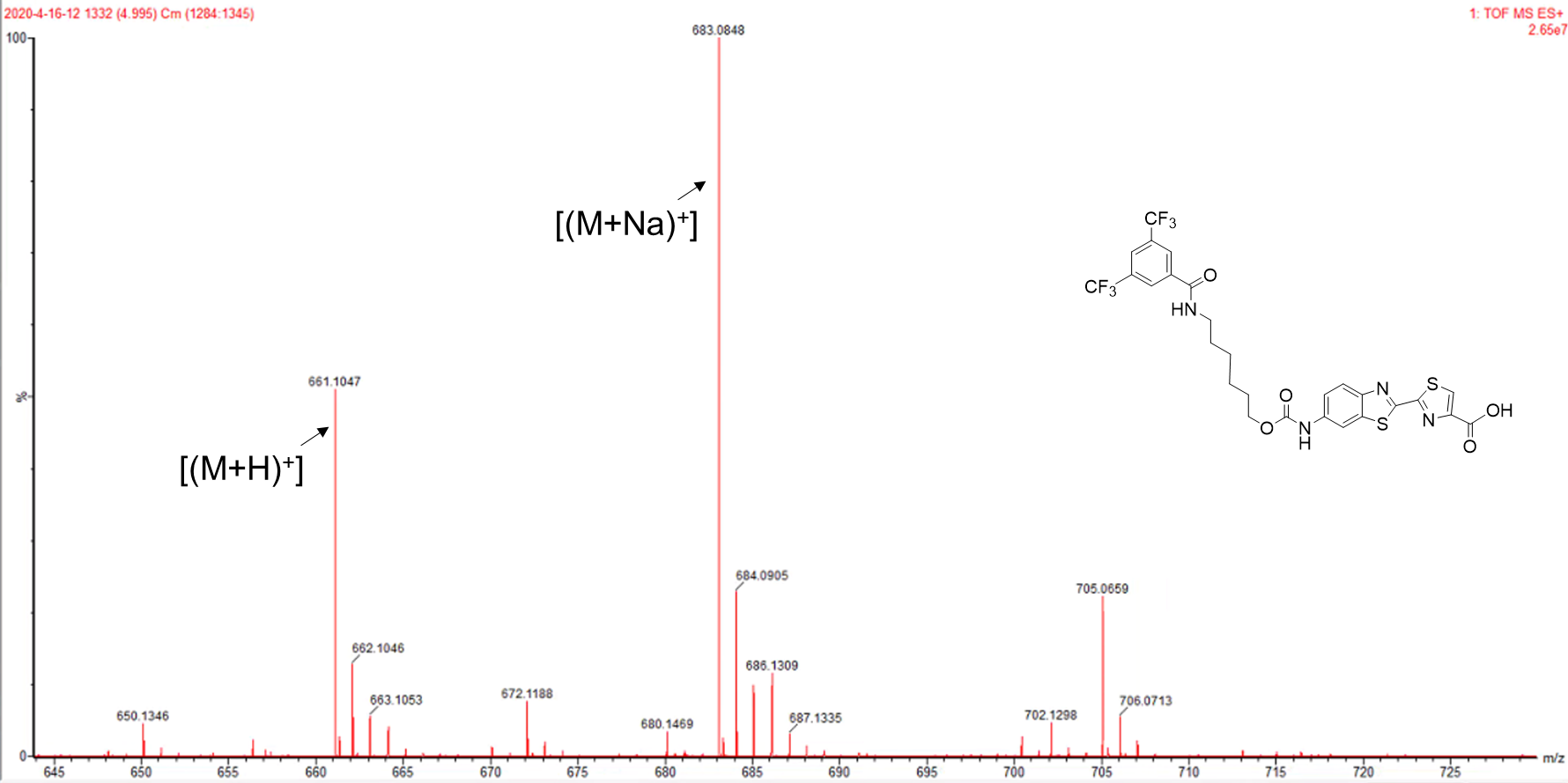


**Figure S62.** ^1^H-NMR spectra of compound **14** (DMSO-*d*_6_)


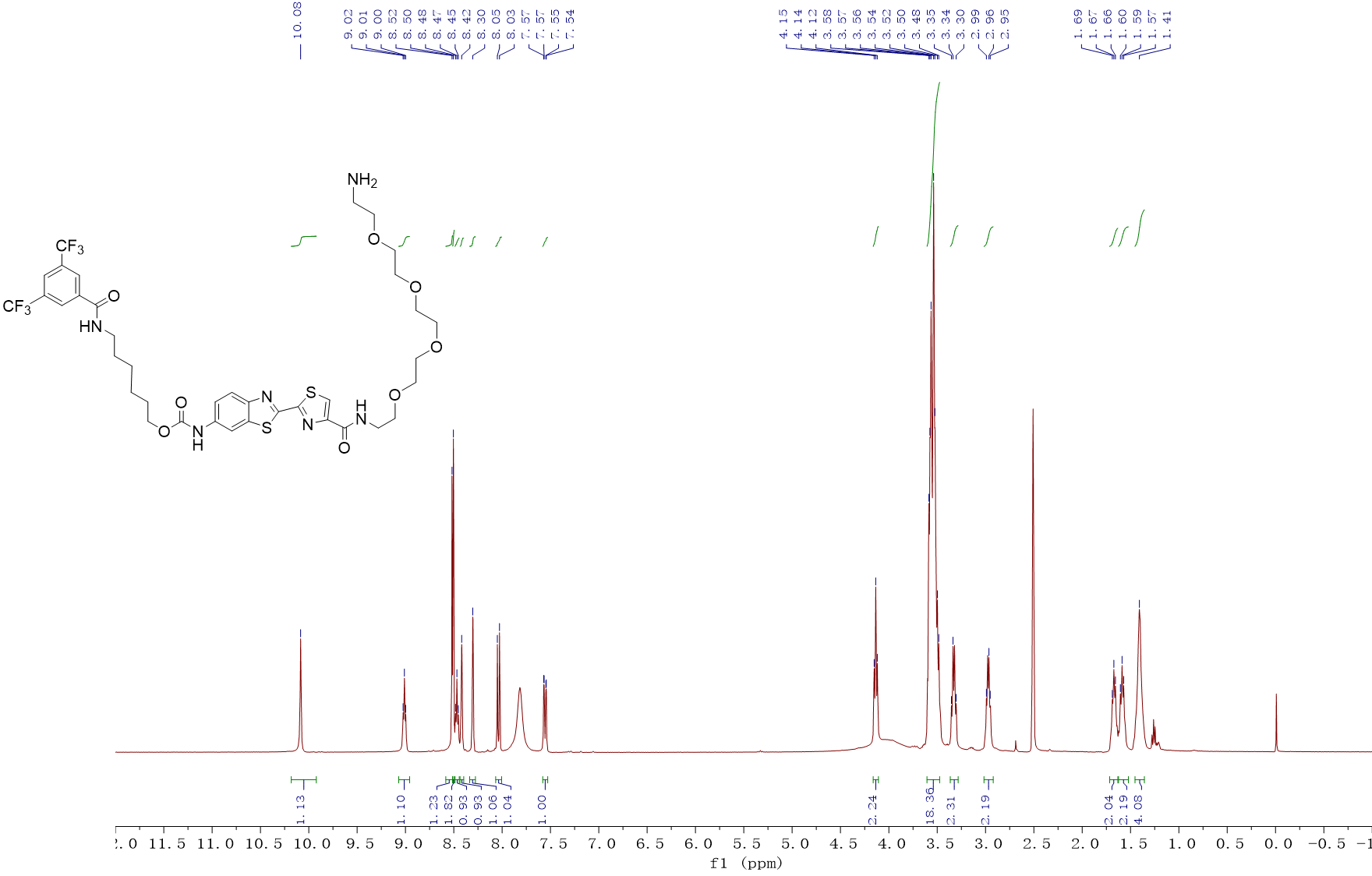


**Figure S63.** ^13^C-NMR spectra of compound **14** (DMSO-*d*_6_)


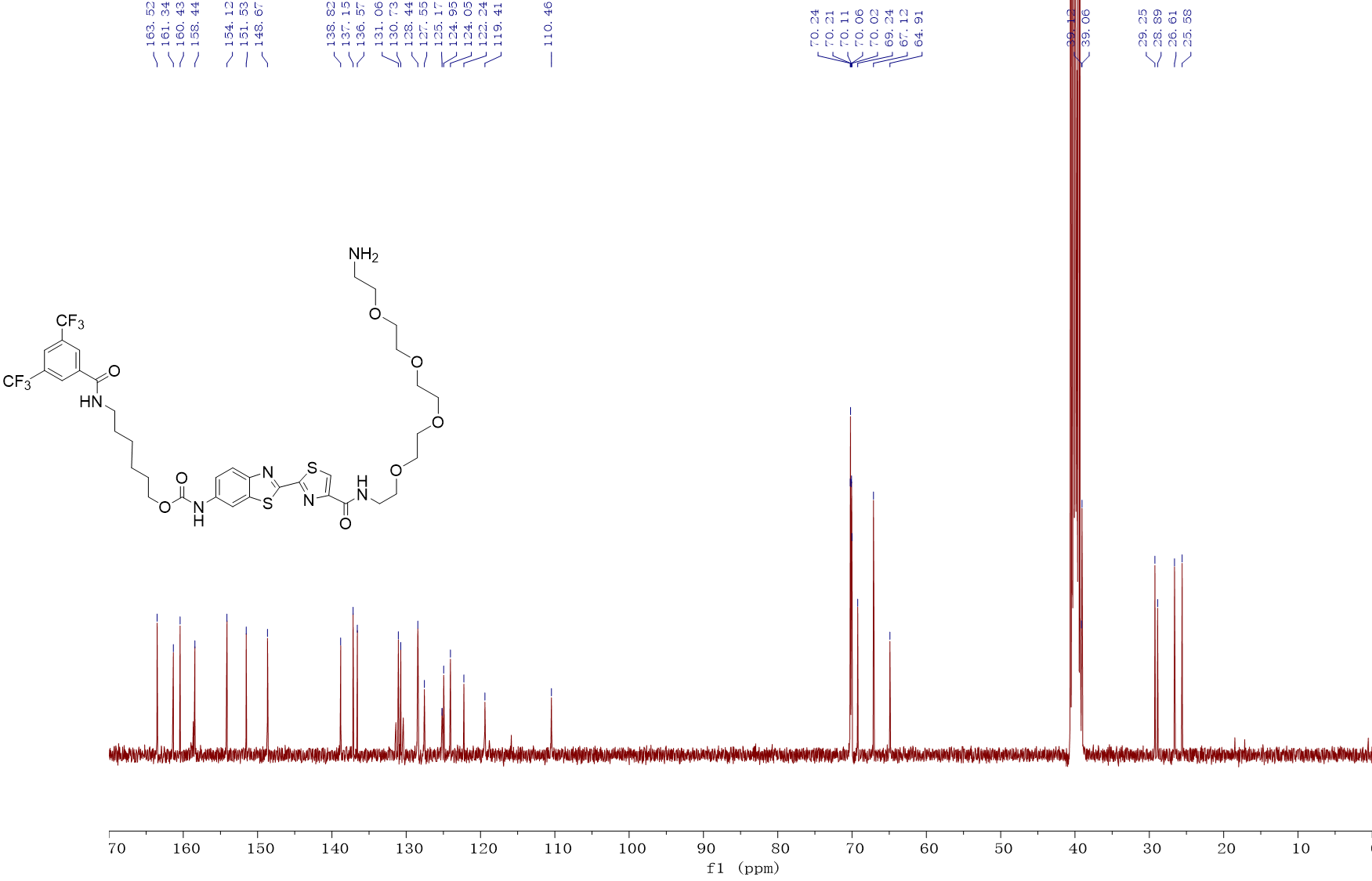


**Figure S64.** HRMS Spectrum of compound **14**


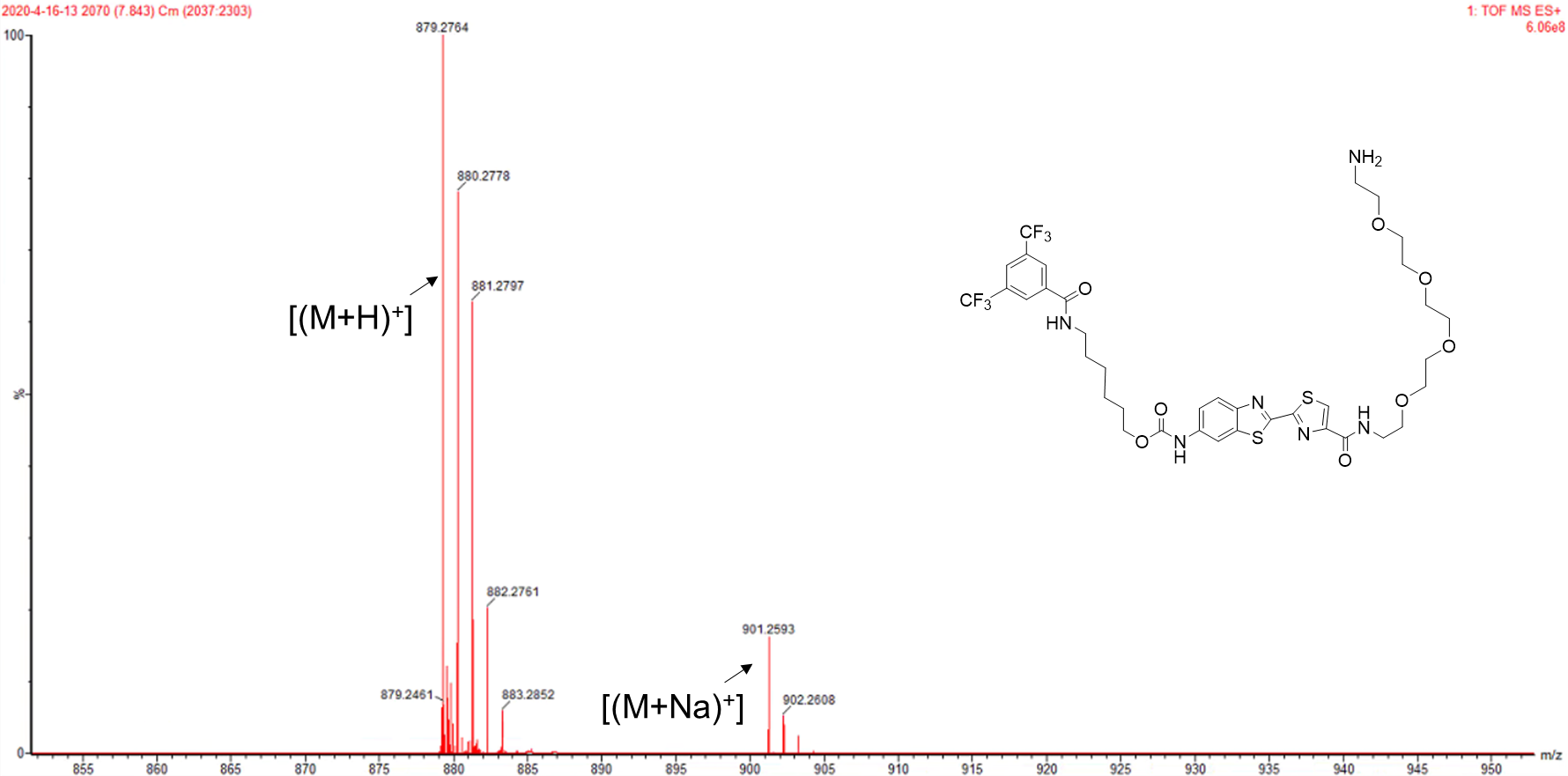


**Figure S65.** ^1^H-NMR spectra of compound **15** (DMSO-*d*_6_)


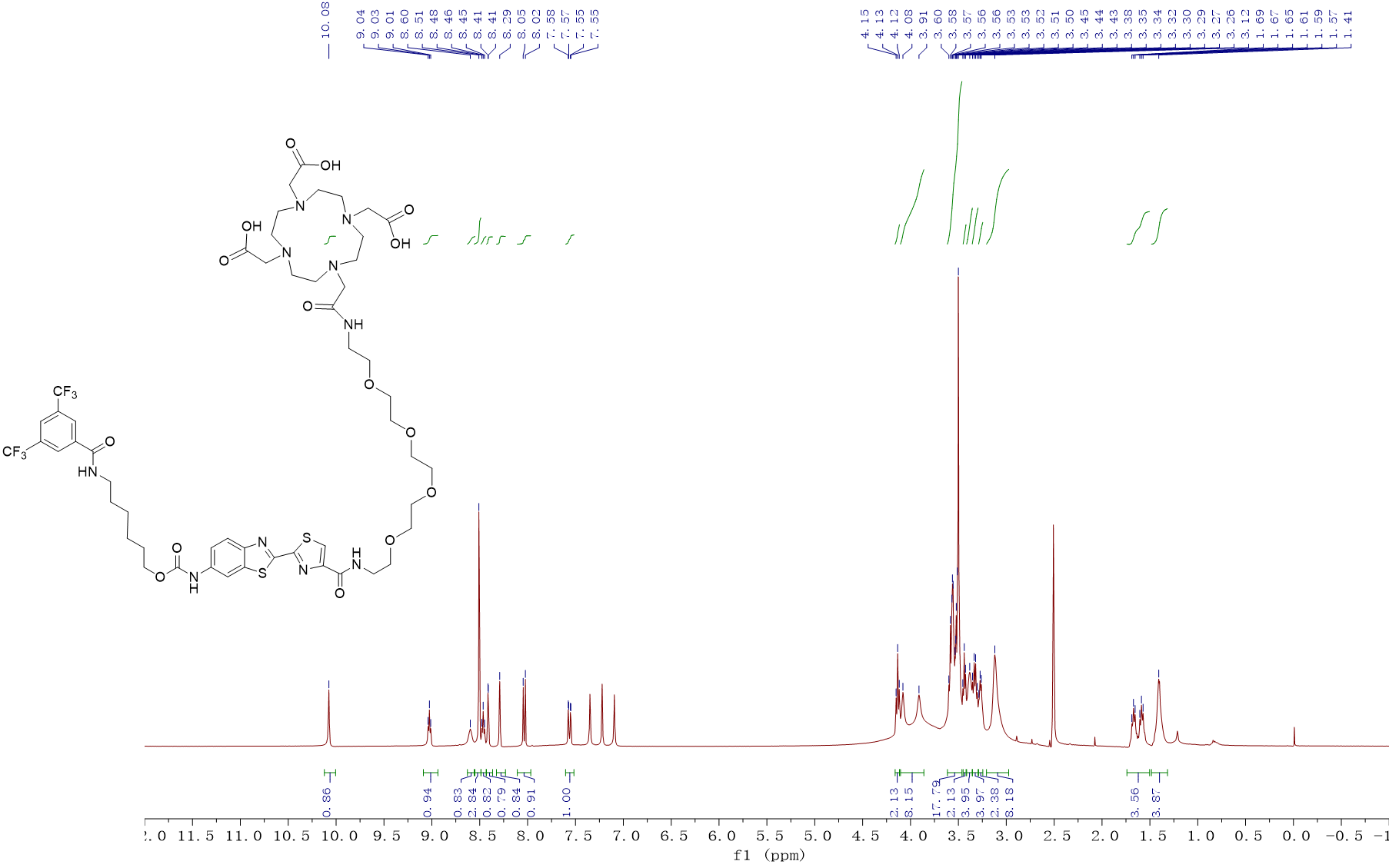


**Figure S66.** ^13^C-NMR spectra of compound **15** (DMSO-*d*_6_)


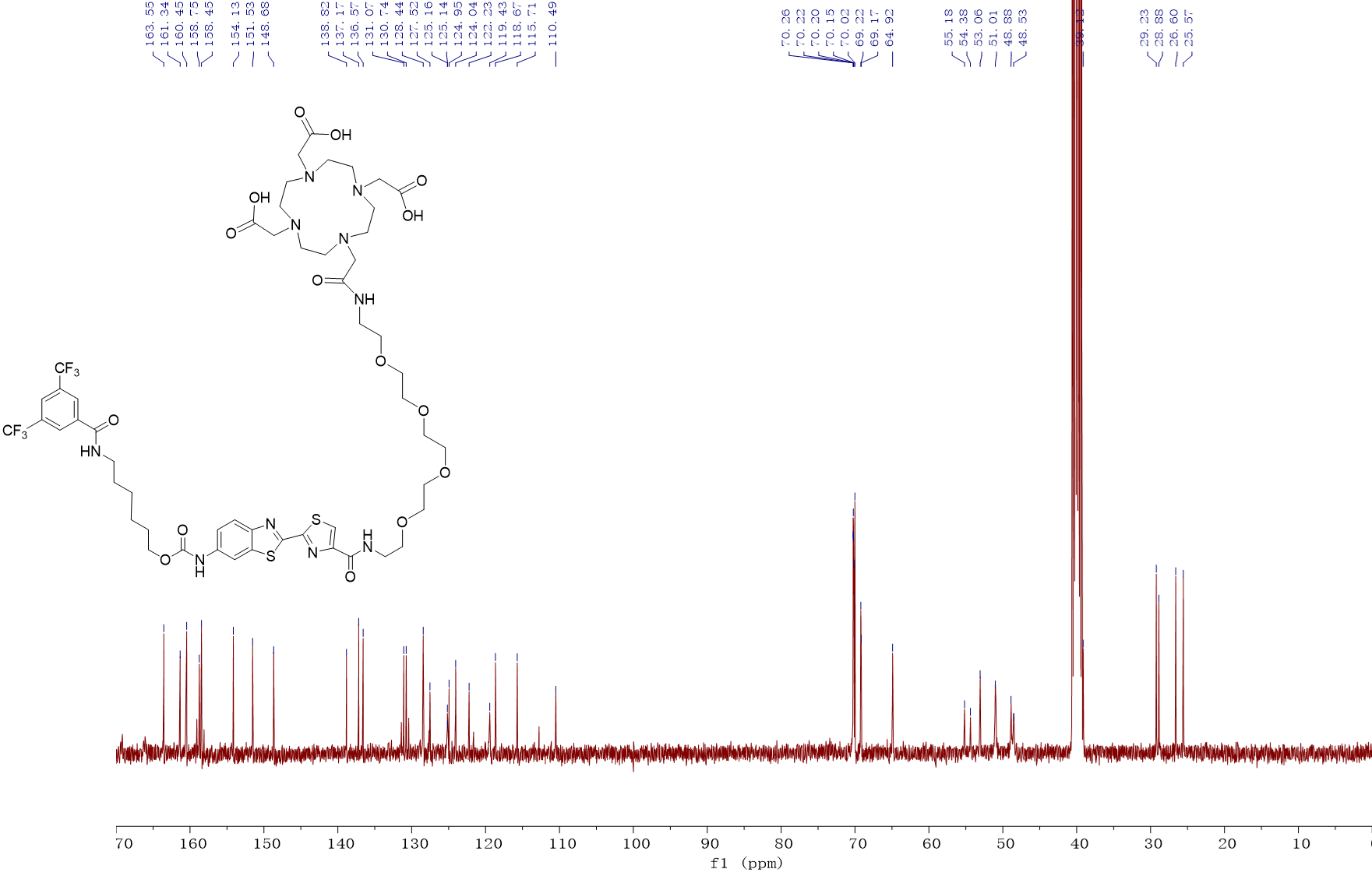


**Figure S67.** MALDI-TOF Spectrum of compound compound **15**


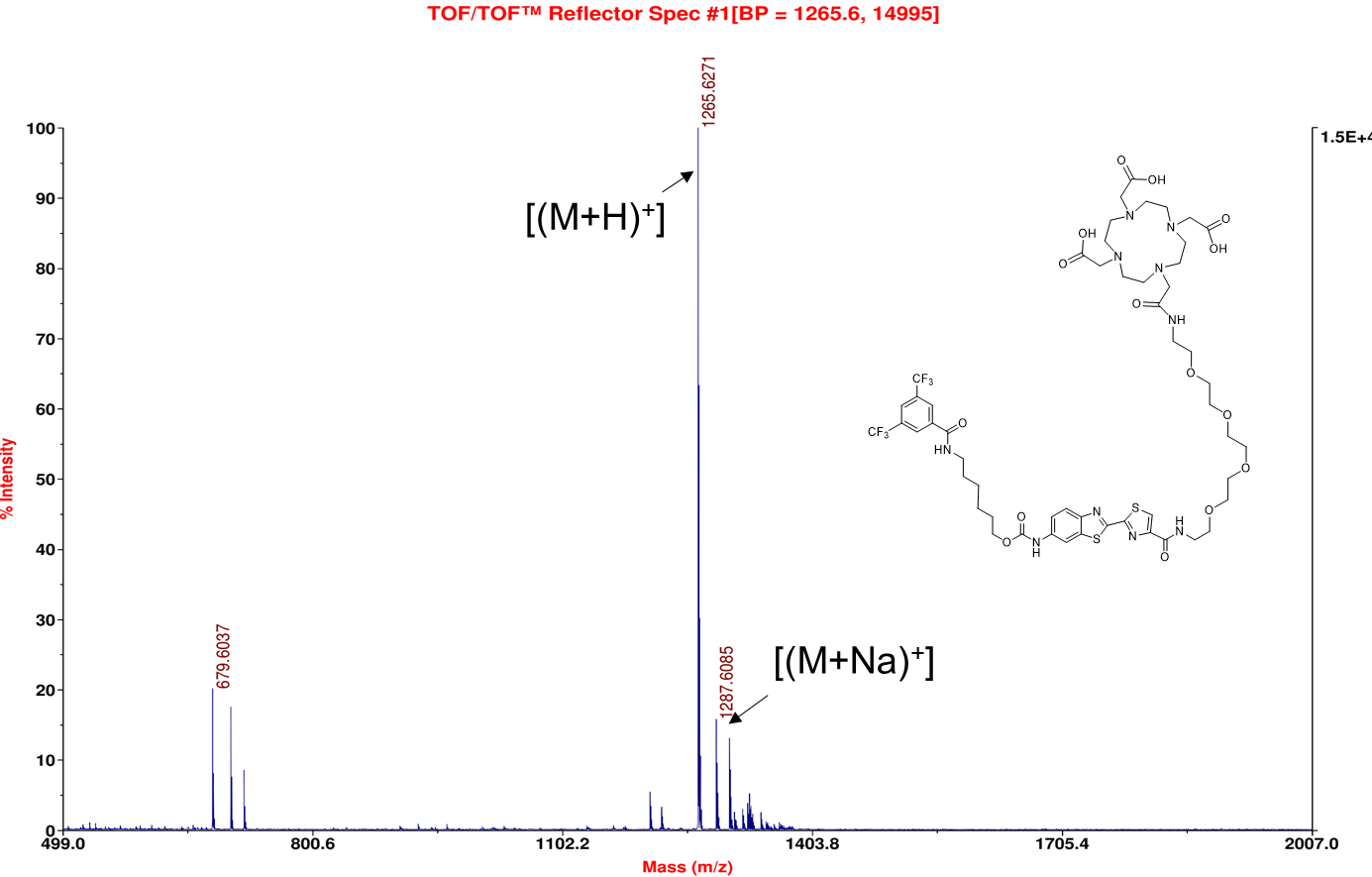


**Figure S68.** HRMS Spectrum of compound **1-Gd-ctrl**


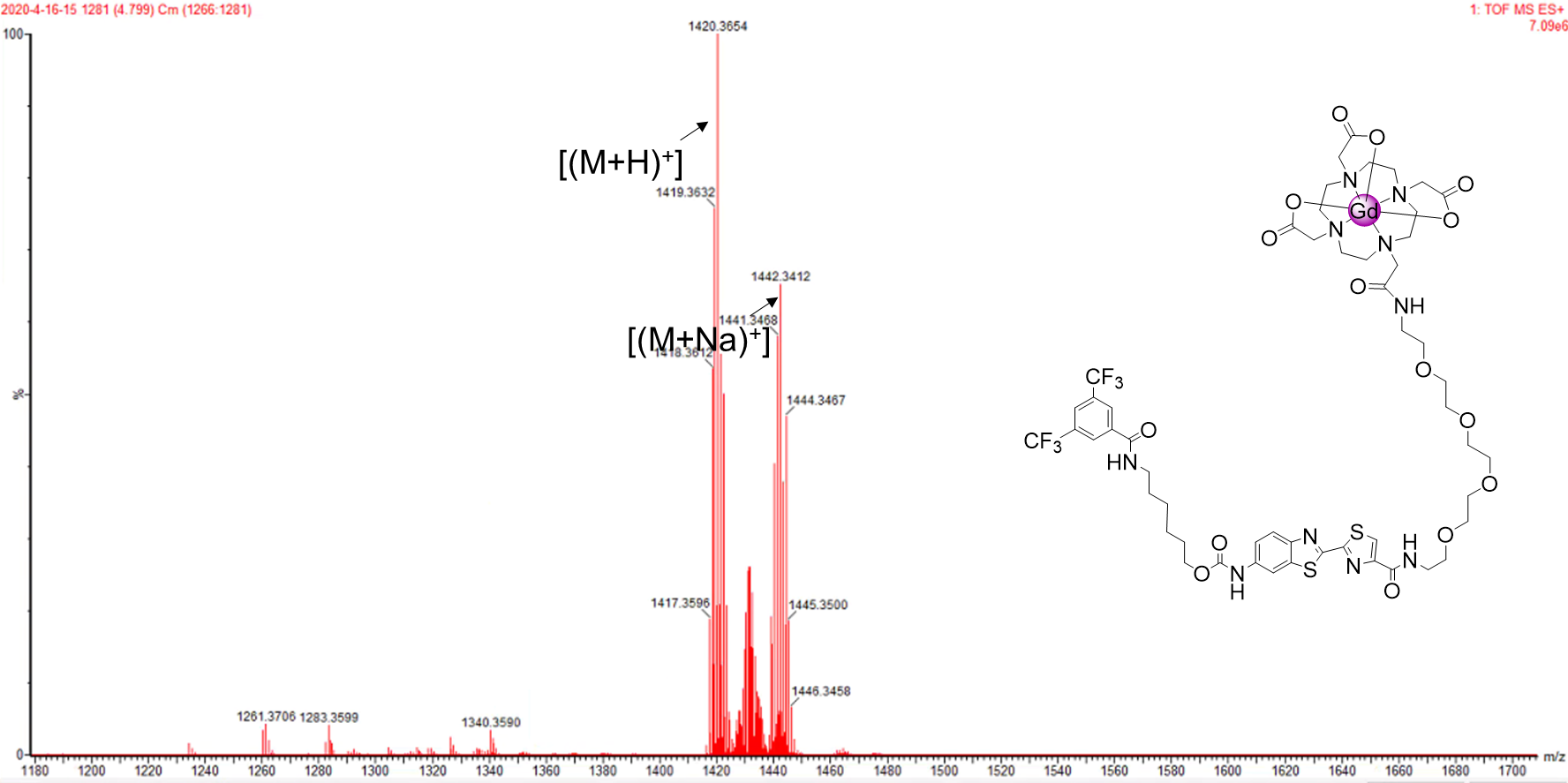


**Figure S69.** ^1^H-NMR spectra of compound **16** (DMSO-*d*_6_)


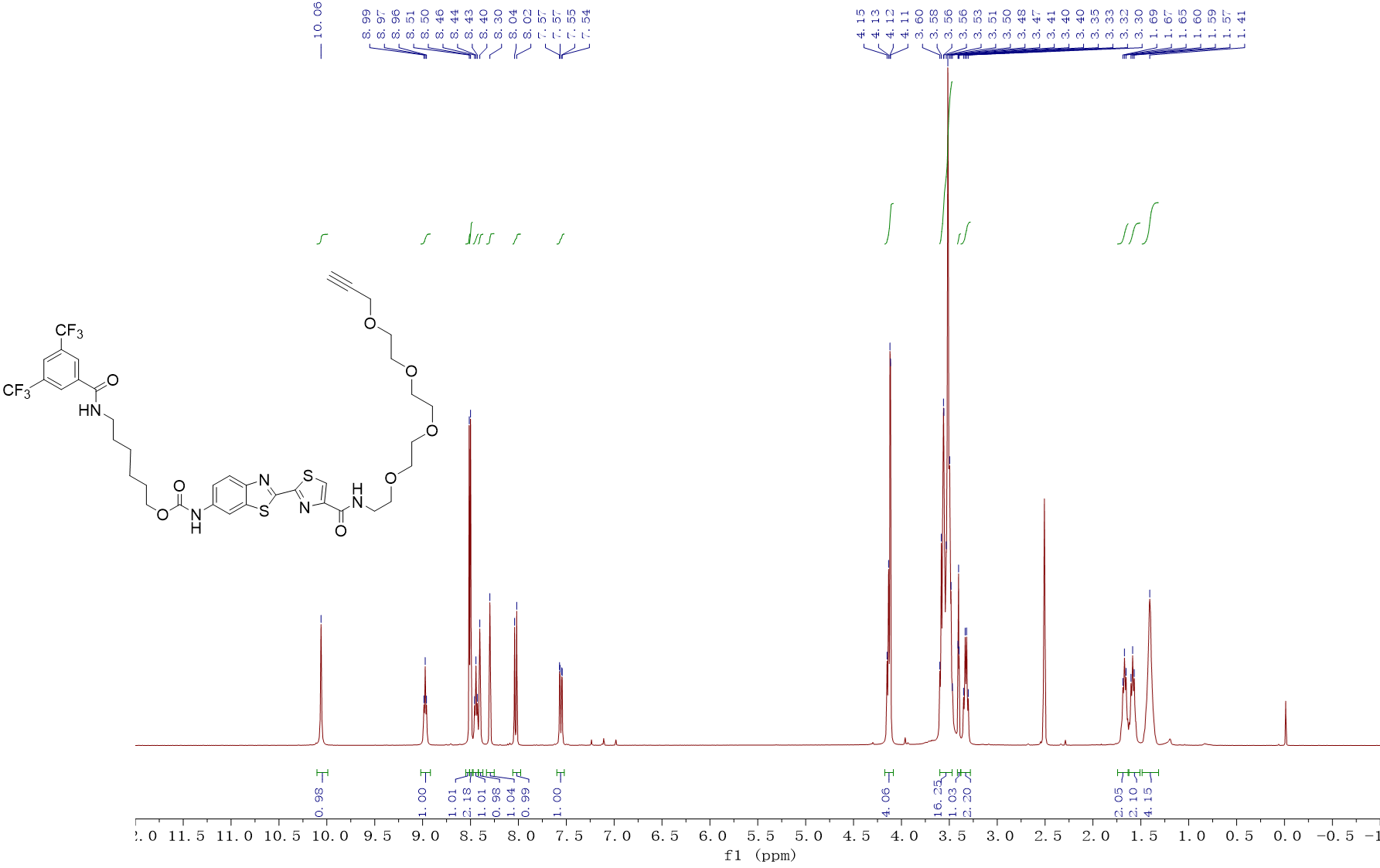


**Figure S70.** ^13^C-NMR spectra of compound **16** (DMSO-*d*_6_)


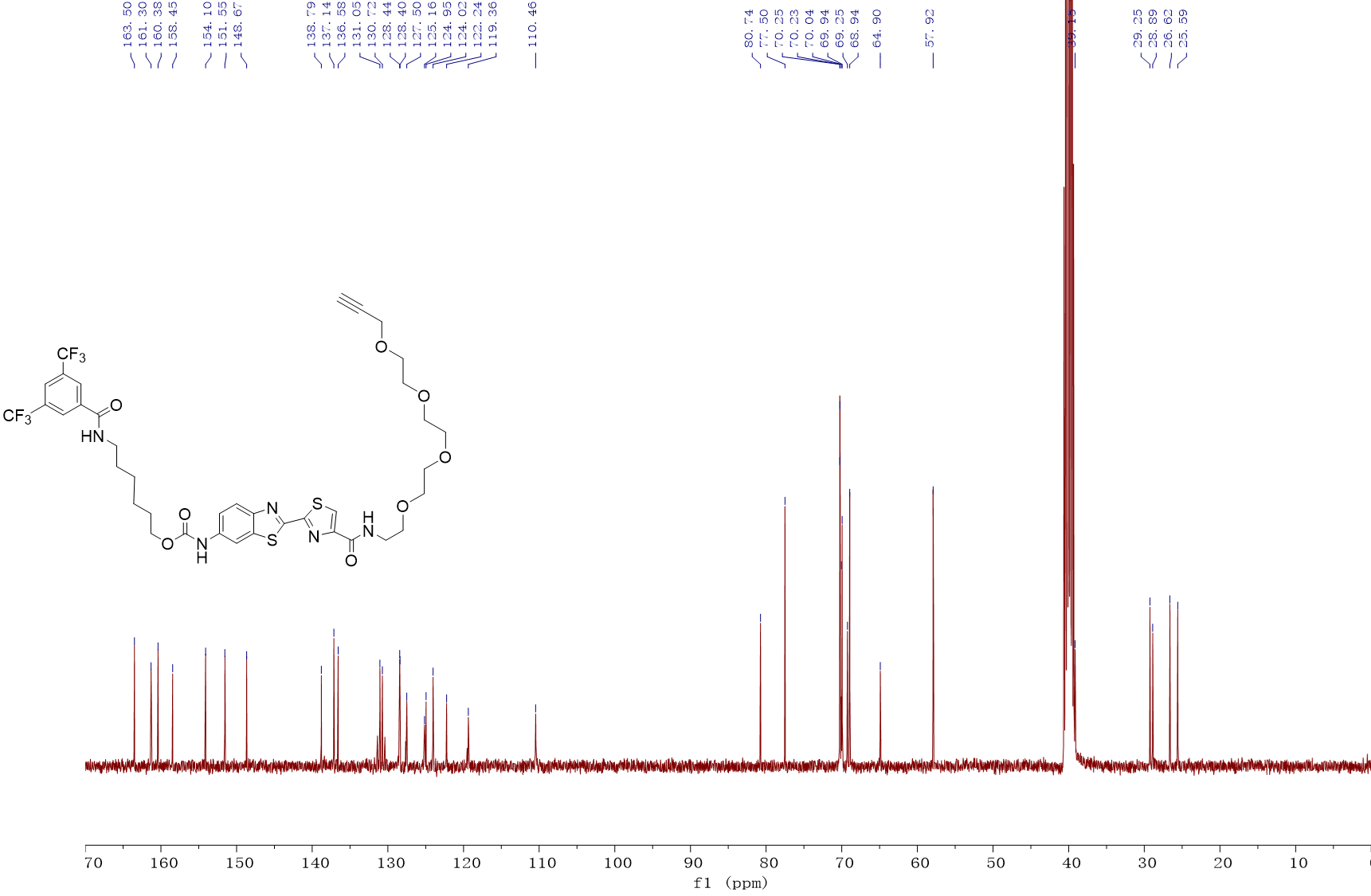


**Figure S71.** HRMS Spectrum of compound **16**


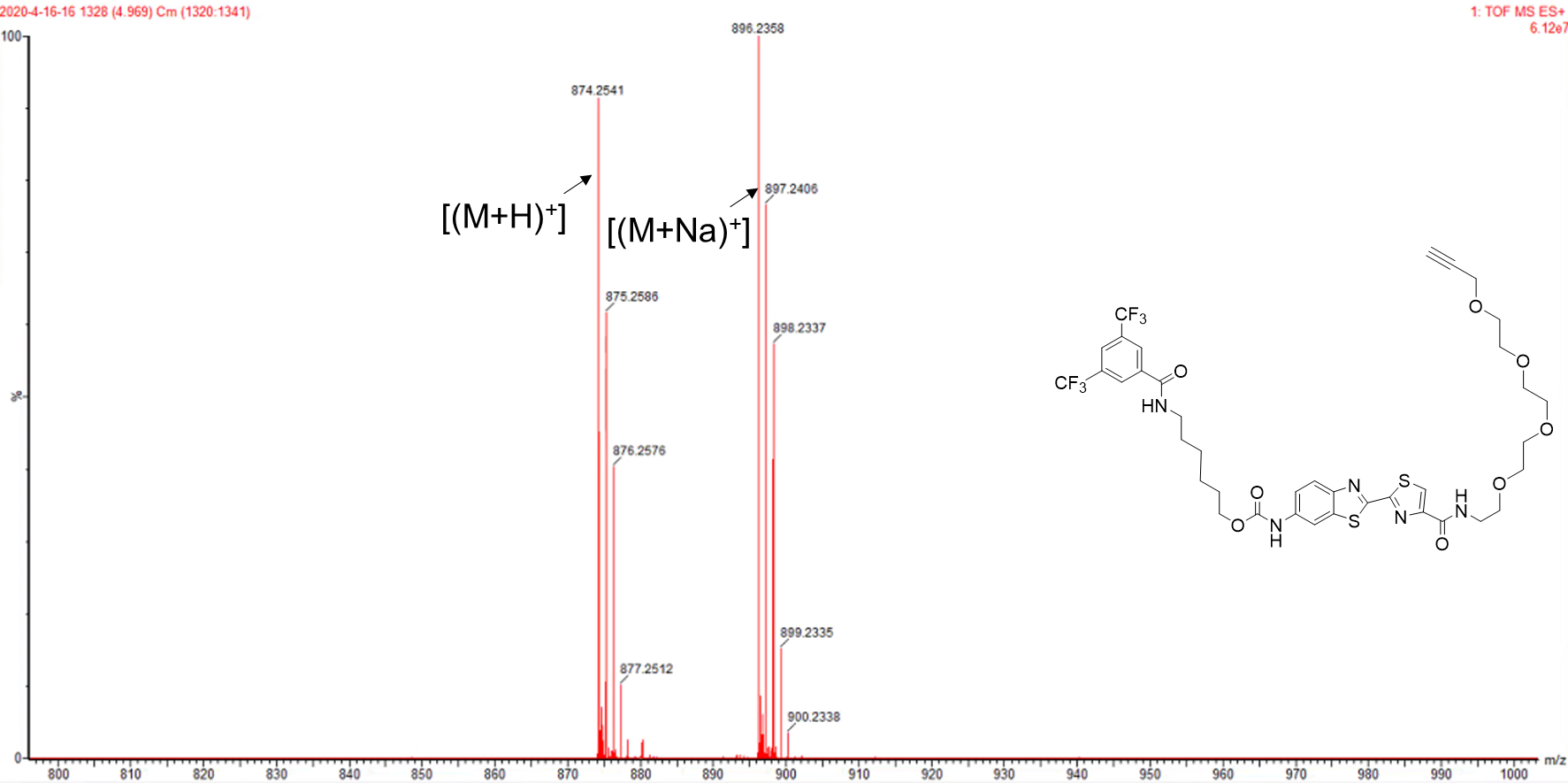


**Figure S72.** ^1^H-NMR spectra of compound **1-Gal-ctl** (DMSO-*d*_6_)


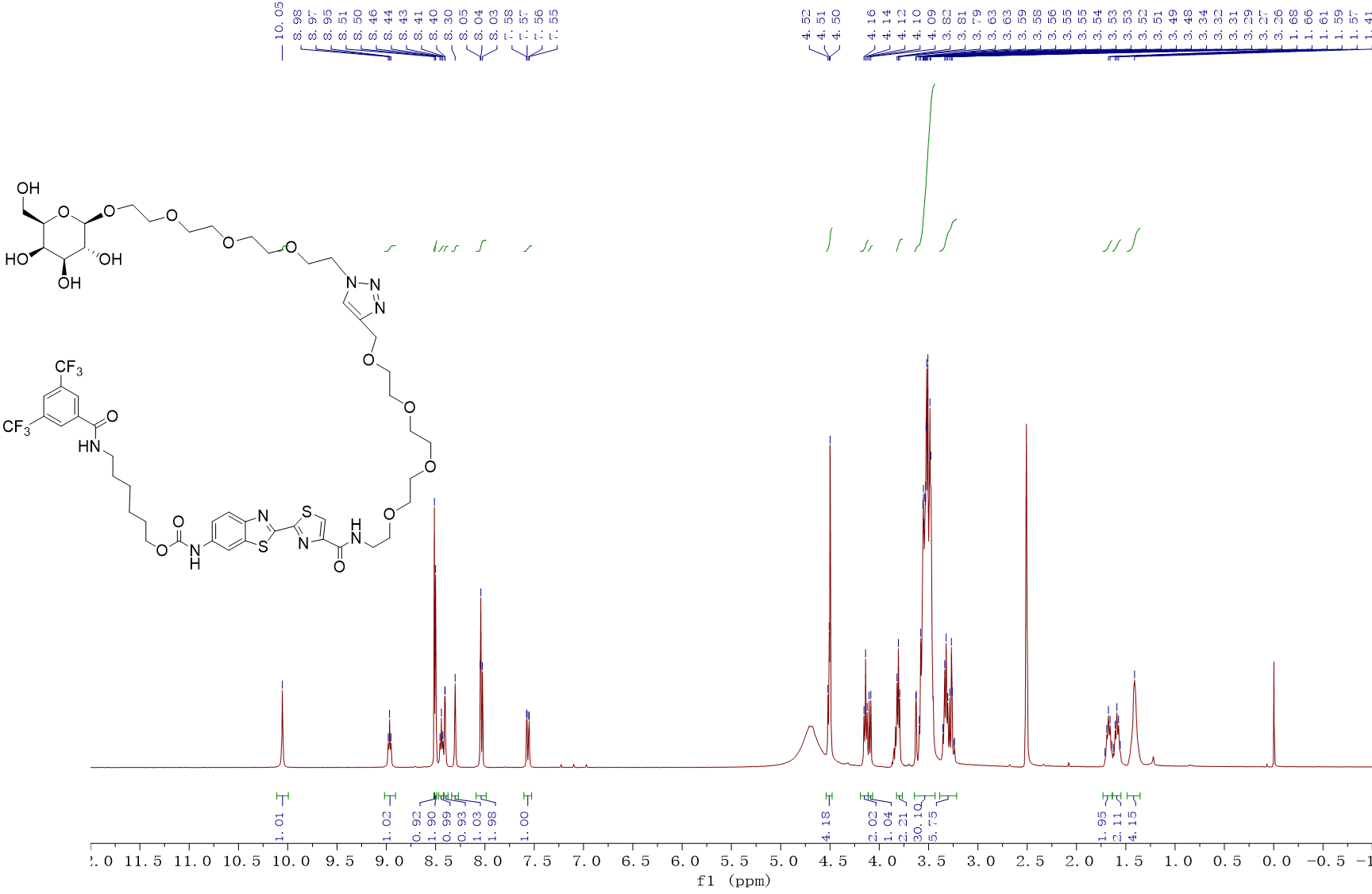


**Figure S73.** ^13^C-NMR spectra of compound **16** (DMSO-*d*_6_)


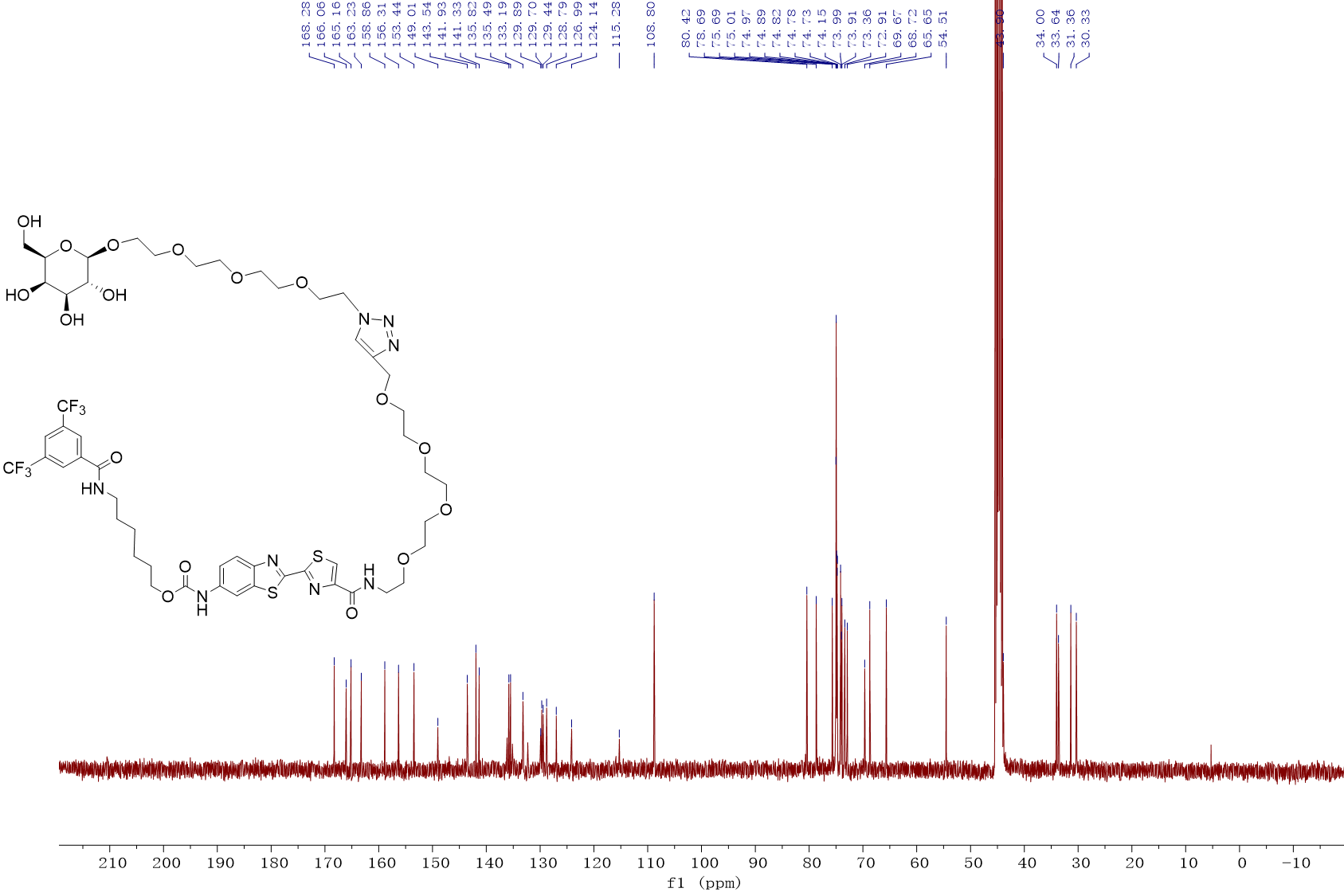


**Figure S74.** MALDI-TOF Spectrum of compound **1-Gal-ctrl**


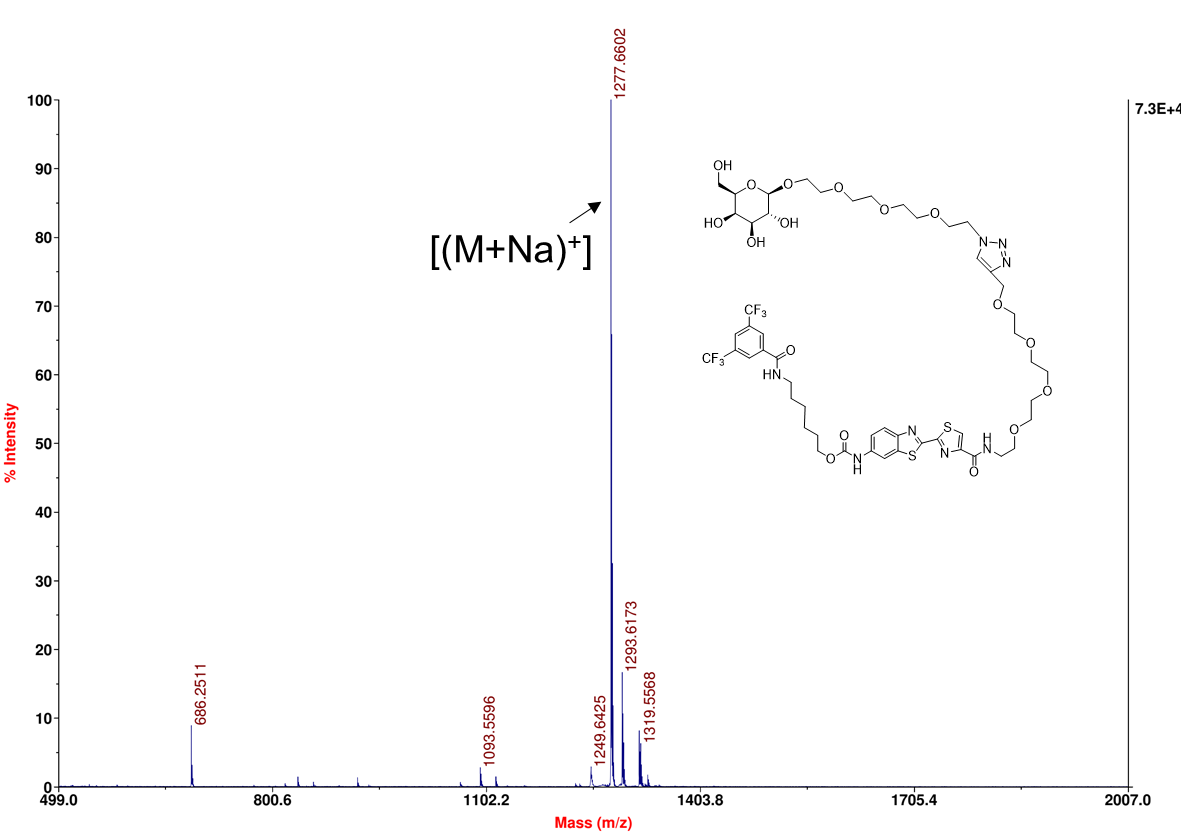

Supplement: Supplementary Materials — Experimental materials and methods, compounds synthesis, supplementary figures, NMR spectra, and MS spectra are shown as Supplementary Figure S31-74, which are available in Supplementary Materials. [file 4087069.f1.docx]
